# A Periodic Hexagon Tiling Model and Non-Hermitian Orthogonal Polynomials

**DOI:** 10.1007/s00220-020-03779-0

**Published:** 2020-05-23

**Authors:** C. Charlier, M. Duits, A. B. J. Kuijlaars, J. Lenells

**Affiliations:** 1 Department of Mathematics, Royal Institute of Technology (KTH), Stockholm, Sweden; 2 Department of Mathematics, Katholieke Universiteit Leuven, Leuven, Belgium

## Abstract

We study a one-parameter family of probability measures on lozenge tilings of large regular hexagons that interpolates between the uniform measure on all possible tilings and a particular fully frozen tiling. The description of the asymptotic behavior can be separated into two regimes: the low and the high temperature regime. Our main results are the computations of the disordered regions in both regimes and the limiting densities of the different lozenges there. For low temperatures, the disordered region consists of two disjoint ellipses. In the high temperature regime the two ellipses merge into a single simply connected region. At the transition from the low to the high temperature a tacnode appears. The key to our asymptotic study is a recent approach introduced by Duits and Kuijlaars providing a double integral representation for the correlation kernel. One of the factors in the integrand is the Christoffel–Darboux kernel associated to polynomials that satisfy non-Hermitian orthogonality relations with respect to a complex-valued weight on a contour in the complex plane. We compute the asymptotic behavior of these orthogonal polynomials and the Christoffel–Darboux kernel by means of a Riemann–Hilbert analysis. After substituting the resulting asymptotic formulas into the double integral we prove our main results by classical steepest descent arguments.

## Introduction

**Fig. 1:**
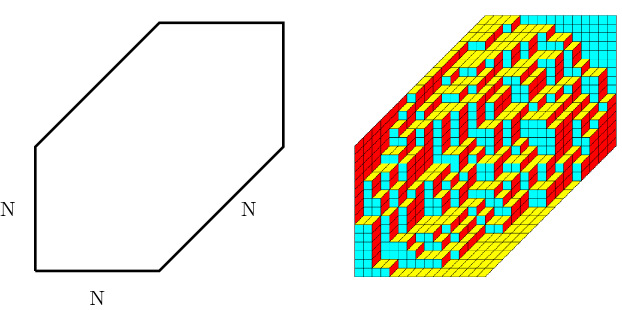
The hexagon (left) and an example of a tiling (right) of the hexagon by lozenges

We study random lozenge tilings of large regular hexagons. We place the regular hexagon so that it has corners at (0, 0), (0, *N*), (*N*, 2*N*), (2*N*, 2*N*), (2*N*, *N*) and (*N*, 0) and consider tilings of the hexagon with the following three types of lozenges 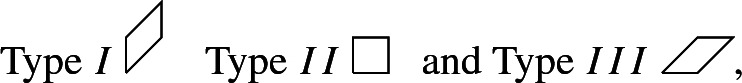 see also Fig. 1. The vertices of the lozenges are on the integer lattice and the vertical and horizontal edges have unit length. There are numerous ways of defining a probability measures on all possible tilings of the hexagon. In this paper, we will be interested in the case in which the probability of a tiling ${\mathcal {T}}$ is given by

$$\begin{aligned} {\mathbb {P}}({\mathcal {T}})= \frac{W({\mathcal {T}})}{\sum _{\widetilde{{\mathcal {T}}}} W(\widetilde{{\mathcal {T}}})}, \end{aligned}$$

where *W* is a weight function on all possible tilings defined bywith1.1for some fixed $\alpha \in (0,1].$ Note that if $\alpha =1$ all tilings occur with the same probability and the probability measure reduces to the uniform measure on all possible tilings. We exclude $\alpha =0$. In the limit $\alpha \downarrow 0$, there is only one possible tiling, see e.g. Fig. 3 below, and there is no randomness. The main results in this paper concern the asymptotic behavior of the random tilings as the size of the hexagon grows large, i.e., as $N\rightarrow \infty$, and how this asymptotic behavior depends on the parameter $\alpha$.

Random tilings of planar domains have been extensively studied in the past decades and we refer to [6, 24–26, 45, 50–52] for important early references, and to [13, 46, 48] for excellent introductions to the topic. When the domains are large, the statistical properties of the tilings are expected to be described by universal limiting processes. In various special classes, and especially in case the random measure is a determinantal point process, tools have been developed to compute the asymptotic behavior and verify the appearance of these universal processes. For instance, if the random measure is in the Schur class [62, 64], then we have a double integral representation for the correlation kernel at our disposal to analyze the fine properties of the model. Random lozenge tilings of the hexagon are however typically not in the Schur class and asymptotic studies are often more complicated.

Although not being in the Schur class, the large *N* behavior of random lozenge tilings of the hexagon with the uniform measure (corresponding to $\alpha =1$ in our setup) has also been intensively studied by various authors. Based on a representation in terms of Hahn polynomials as found in [44] (see also [43]), the authors of [6] managed to perform a steepest descent analysis of the discrete Riemann–Hilbert (RH) problem for the Hahn polynomials and, consequently, describe the limiting disordered regions and the local universality laws. In [43] the local universality was obtained using methods developed in [16]. In a more general context, uniform lozenge tilings of more complicated domains were studied by means of double integral formulas [3, 35–37, 66, 67].

An important part of the recent literature on random tilings is concerned with proving the universality of the global fluctuations and the emergence of the Gaussian Free Field. For the uniform measure on all possible tilings of the hexagon there are now various techniques in the literature that prove this claim. In [67] the convergence of the global height fluctuations to the Gaussian Free Field was established using double integral formulas for the kernel. An alternative proof based on the recurrence coefficients of the Hahn polynomials was given in [33] extending the results on the fluctuations along vertical sections in [18]. Discrete loop equations can also be used [14] to compute the fluctuations along vertical sections. In [19, 20], another approach is introduced using the notion of a Schur generating function. Each of these methods apply to their own general class of models and contain the uniform measure as a special case.

Measures on tilings of the (finite) hexagon that are not uniform are known to be difficult to analyze asymptotically and much less results are known. For instance, in [15] the authors introduced elliptic weights on the lozenge tilings, but a full asymptotic study of these models is still open. The situation $0<\alpha < 1$, which is the topic of this paper, is a rather gentle way to break the uniform measure. Still, the above mentioned techniques do not apply. To study our model we will use a recently developed new approach [34] for studying determinantal point processes that are defined via products of minors of (scalar or block) Toeplitz minors. Although the original motivation of [34] was to analyze the so-called 2-periodic Aztec diamond (see also [7, 22]), the methods apply to a much wider range of (tiling) models. The approach mainly consists of combining two important methods for asymptotic analysis: the classical steepest descent method for integrals and the Deift/Zhou steepest descent method for RH problems [27, 29]. This opens up new possibilities for analyzing models that were thus far out of reach and the model studied in this paper is one such example.

It is possible to take the limit of our model in which the vertical sides of the hexagon tend to infinity (see, for example, [11] for an explanation that starts from the same setting as in the present paper). In that limit, our model is the same as a 2-periodic weighting of plane partitions against a linearly shaped back wall, as studied in [60] (see also [5] for a generalization to the setting of Macdonald processes). This model is then in the Schur class and thus double integral representations are available for asymptotic studies. It is important to note that the case of a finite hexagon does not only lead to technical challenges, but also more complicated phenomena occur. For instance, in our model a tacnode appears for $\alpha = 1/9$.

**Fig. 2:**
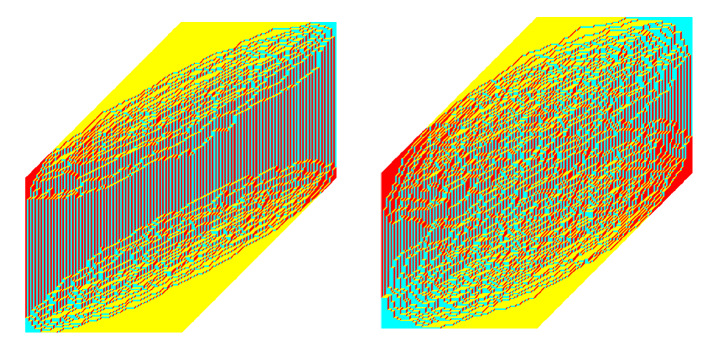
Two sample tilings corresponding to the low temperature (left) and high temperature (right) regimes, respectively

In Fig. 2 we have plotted two sample tilings for large hexagons, one with $0< \alpha <\frac{1}{9}$ and the other with $\frac{1}{9}< \alpha <1$. We see that for $0< \alpha <\frac{1}{9}$ there appear two clouds in which the tiling shows randomness, while it is frozen outside. In the figure with $\frac{1}{9}< \alpha <1$, these two clouds seem to have merged. To understand why this phenomenon is happening, it is useful to view $\alpha$ as a temperature parameter. Indeed, after defining the energy of a tiling aswe can write the weight of a tiling ${\mathcal {T}}$ as $W({\mathcal {T}})= e^{(\log \alpha ) {\mathcal {E}}({\mathcal {T}})}$, and its probability as

$$\begin{aligned} {\mathbb {P}}(\mathcal T) = \frac{1}{Z} e^{-\beta {\mathcal {E}}({\mathcal {T}})}, \qquad \beta = - \log \alpha \end{aligned}$$

which is a Gibbs measure with inverse temperature $\beta$. Thus, $T= -\frac{1}{\log \alpha }$ may (and will) be viewed as the temperature parameter. The low temperature limit $T \downarrow 0$ corresponds to $\alpha \downarrow 0$ and the high temperature limit $T \rightarrow \infty$ to $\alpha \uparrow 1$.

**Fig. 3:**
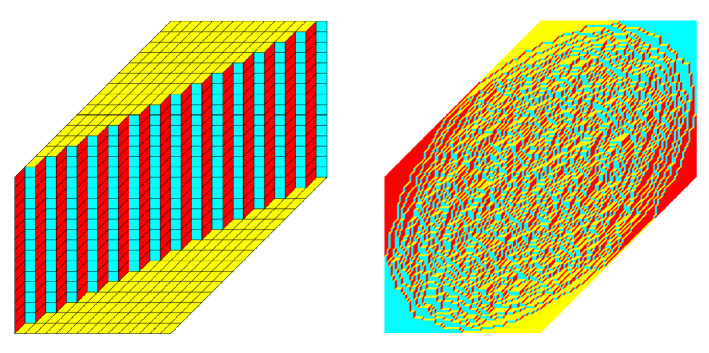
The two extreme cases: $\alpha =1$ leading to the uniform measure (right) and $\alpha =0$ for which there is only one possible tiling (left)

For low temperatures, the number ${\mathcal {E}}({\mathcal {T}})$ is expected to be small. In fact, for $T\downarrow 0$ the randomness disappears and the lozenge configurations freeze to the unique tiling with ${\mathcal {E}}({\mathcal {T}})=0$. This is the tiling that is shown in the left half of Fig. 3. It can be thought of as a staircase shaped wall where the floor and the ceiling only have tiles of type III. As the temperature increases, randomness starts appearing near the interfaces where the wall meets the ceiling and the floor. For *T* positive but small, we expect to observe two separate clouds that are far away from each other. When *T* increases further, the clouds meet and form one cloud. Eventually, as $T\rightarrow \infty$, the model becomes the uniform measure on tilings and the cloud becomes the ellipse that is inscribed in the hexagon, as in the right part of Fig. 3.

In other words, we expect that there is a critical point in the low to high temperature transition at which the topology of the disordered regime changes from being disconnected to being connected. As we will see, this transition indeed happens at $\alpha =\frac{1}{9}$. We will therefore speak of $0<\alpha <\frac{1}{9}$ as the *low temperature regime* and of $\frac{1}{9} < \alpha \le 1$ as the *high temperature regime.*

Our analysis follows a recent work [34]. The backbone of the approach in [34] is a connection to polynomials that satisfy an orthogonality relation (that could be matrix valued) on a contour in the complex plane. In the present paper we will be dealing with scalar orthogonality on a closed contour $\gamma$ going once around the origin with counterclockwise orientation. Let $p_n$ be the monic polynomial of degree *n* such that

1.2 $$\begin{aligned} \frac{1}{2\pi i} \oint _\gamma p_n(z) z^j \frac{(z+1)^N (z+\alpha )^N}{z^{2N}}\ dz= 0, \qquad j=0,1,\ldots , n-1. \end{aligned}$$

It is important to note that (1.2) is an orthogonality condition with respect to a non-Hermitian bilinear form. It is therefore not evident that the polynomials $p_n$ are well-defined. We will prove that they are, provided that $n \le 2N$, see Proposition 5.1. The orthogonality (1.2) also changes with *N*, the size of the hexagon.

It turns out that the random tilings naturally define a determinantal point process with a correlation kernel that can be expressed in terms of the polynomials $p_n$. For the exact statement, we need to introduce a well-known correspondence between tilings of the hexagon and non-intersecting paths. For more background on determinantal point processes, random tilings and non-intersecting paths, we refer to [46].

We draw lines on two of the three types of lozenges as follows: 
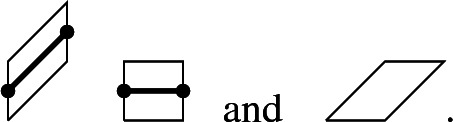
 The paths form a collection of non-intersecting paths $\pi _j: \{0,\ldots , 2N\} \rightarrow {\mathbb {Z}}+\tfrac{1}{2}$ with initial points $\pi _j(0)=j+\tfrac{1}{2}$ and endpoints $\pi _j(2N)=N+\tfrac{1}{2}+j$ for $j=0, \ldots ,N-1$. It is well-known and easy to see that there is a one-to-one correspondence between tilings of the hexagon and non-intersecting up-right paths with these initial and end configurations. The probability measure on the tilings defined in (1.1) induces a probability measure on such collections of non-intersecting paths. The Lindström–Gessel–Viennot lemma [41, 55] tells us that the probability measure is proportional to

1.3 $$\begin{aligned} \prod _{m=0}^{2N-1} \det \left[ T_m\left( \pi _j(m)-\tfrac{1}{2},\pi _k(m+1)-\tfrac{1}{2}\right) \right] _{j,k=1}^{N}, \end{aligned}$$

where the $T_m$ are ${\mathbb {Z}} \times {\mathbb {Z}}$ matrices given by

1.4 $$\begin{aligned} T_m(x,y)= {\left\{ \begin{array}{ll} \alpha ,&{} \text { if } y=x,\\ 1, &{} \text { if } y=x+1, \\ 0, &{} \text { otherwise}, \end{array}\right. } \end{aligned}$$

if *m* is even, and

1.5 $$\begin{aligned} T_m(x,y)= {\left\{ \begin{array}{ll} 1,&{} \text { if } y=x \text { or } y = x+1,\\ 0, &{} \text { otherwise}, \end{array}\right. } \end{aligned}$$

if *m* is odd. The probability (1.3) is a determinantal point process with a correlation kernel given by the Eynard–Metha formula [38].

In case the ${\mathbb {Z}} \times {\mathbb {Z}}$ matrices $T_m$ in (1.3) are (scalar or block) Toeplitz matrices, the paper [34] gives a double contour integral formula for the correlation kernel, which involves the (scalar or block) symbols of the Toeplitz matrices as well as a reproducing kernel for (scalar or matrix-valued) orthogonal polynomials, see also [8].

The matrices (1.4) and (1.5) are infinite Toeplitz matrices with only two non-zero diagonals. Their respective symbols are $z+\alpha$ and $z+1$. Both Toeplitz matrices appear *N* times in the product (1.3) and this accounts for the orthogonality measure in (1.2). Then the general formula in [34] reduces to the following in the special situation of this paper.

### Proposition 1.1

Let $\alpha \in (0,1]$ and let $k \ge 1$ be an integer. Then for integers $x_1, \ldots , x_k$, $y_1, \ldots , y_k$, with $(x_i,y_i) \ne (x_j,y_j)$ if $i \ne j$, we have

1.6 $$\begin{aligned} {\mathbb {P}}\left[ \begin{array}{l} \text {paths go through each of the points } \\ (x_1,y_1+\tfrac{1}{2}), \ldots , (x_k,y_k+\tfrac{1}{2}) \end{array} \right] =\det \left[ K_N(x_i,y_i,x_j,y_j)\right] _{i,j=1}^k, \end{aligned}$$

where the kernel $K_N$ is given by

1.7 $$\begin{aligned} K_N(x_1,y_1,x_2,y_2)= & {} -\frac{\chi _{x_1>x_2}}{2 \pi i} \oint _\gamma (z+1)^{\lfloor \frac{x_1}{2} \rfloor -\lfloor \frac{x_2}{2} \rfloor } (z+\alpha )^{\lfloor \frac{x_1+1}{2} \rfloor -\lfloor \frac{x _2+1}{2}\rfloor } \frac{dz}{z^{y_1-y_2+1}} \nonumber \\&+ \frac{1}{(2\pi i)^2} \oint _\gamma \oint _\gamma R_N(w,z)\nonumber \\&\frac{(w+1)^N(w+\alpha )^N }{w^{2N}} \frac{(z+1)^{\lfloor \frac{x_1}{2}\rfloor }(z+\alpha )^{\lfloor \frac{x_1+1}{2}\rfloor }}{(w+1)^{\lfloor \frac{x_2}{2} \rfloor }(w+\alpha )^{\lfloor \frac{x_2+1}{2}\rfloor }} \frac{w^{y_2}}{z^{y_1+1}} dz dw,\nonumber \\ \end{aligned}$$

for $y_1,y_2 \in {\mathbb {Z}}$ and $x_1,x_2 \in \{1,\ldots ,2N-1\}$. Here $\lfloor x\rfloor$ denotes the largest integer $\le x$ as usual, $\chi _{x_1 > x_2} = 1$ if $x_1>x_2$ and 0 otherwise, $\gamma$ is a closed contour that goes once around 0 in counterclockwise direction, and $R_N(w,z)$ is the *N*th Christoffel–Darboux kernel for the orthogonal polynomials $p_n$ defined by

1.8 $$\begin{aligned} R_N(w,z)&= \sum _{n=0}^{N-1} \frac{p_n(w)p_n(z)}{\kappa _n} \nonumber \\&= \kappa _{N-1}^{-1}\frac{p_N(z) p_{N-1}(w)-p_N(w)p_{N-1}(z)}{z-w} \end{aligned}$$

and

1.9 $$\begin{aligned} \kappa _n = \frac{1}{2 \pi i} \oint _\gamma ( p_n(z))^2 \frac{(z+1)^N(z+\alpha )^N}{z^{2N}} \ dz, \end{aligned}$$

is the squared ‘norm’ of $p_n$.

### Proof

This is a special case of [34, Theorem 4.7], but for convenience of the reader we give more details on how to make the identification in the Appendix. $\quad \square$

The above proposition is the starting point of our analysis. Clearly, to analyze the limiting behavior of the probabilities (2.27) it suffices to compute the asymptotic behavior of the kernel $K_{N}$ in (1.7) as $N\rightarrow \infty$. To this end, we first compute the asymptotic behavior of the Christoffel–Daroux kernel $R_N$ corresponding to the orthogonal polynomials using Riemann–Hilbert techniques. After inserting the resulting asymptotics of $R_N$ into (1.7), we compute the asymptotic behavior of $K_{N}$ by a saddle point analysis. It should not come as a surprise to the experienced reader that there many possible fallpits and one may view the fact that this approach can indeed be carried out as the main result of our paper. With this approach one can, in principle, compute all fine asymptotic properties of the model. In an effort to limit the length of the paper, we restrict our main results to the description of the disordered region and the densities of the different types of lozenge there. We will though briefly comment on possible other limiting results that are within reach.

## Statement of Results

In this section we state our main results. The proofs are postponed to later sections.

### Preliminaries

Our main result concerns the limiting densities of the lozenges as the size of the hexagon goes to infinity. We introduce the scaled variables $(\xi ,\eta )$ in the large *N* limit by

2.1 $$\begin{aligned} {\left\{ \begin{array}{ll} \frac{x}{N}\rightarrow 1+\xi ,\\ \frac{y}{N}\rightarrow 1+ \eta , \end{array}\right. } \end{aligned}$$

where the point $(\xi ,\eta )$ belongs to the hexagon

$$\begin{aligned} {\mathcal {H}}= \left\{ (\xi ,\eta ) \mid -1\le \xi \le 1, \ -1 \le \eta \le 1,\ -1\le \eta -\xi \le 1 \right\} . \end{aligned}$$

We will study the following probabilities2.2Here (*x*, *y*) is the coordinate for the black dot. From simple geometric considerations, we note that these probabilities add up to 1. Our main result, Theorem 2.5 below, gives the limits of the probabilities (2.2) under the scaling (2.1) provided that $(\xi ,\eta )$ belongs to the liquid region. The result is stated in terms of a saddle point for the double contour integral in (1.7). The saddle points turn out to be solutions of an algebraic equation

2.3 $$\begin{aligned} \left( \frac{\xi }{2} \left( \frac{1}{z+1} + \frac{1}{z+\alpha } \right) - \frac{\eta }{z} \right) ^2 = Q_{\alpha }(z) \end{aligned}$$

with a rational function $Q_{\alpha }$ that we describe next. The liquid region ${\mathcal {L}}_{\alpha }$ is characterized by the property that (2.3) has a solution $z= s(\xi ,\eta ;\alpha )$ in the upper half plane.

### The rational function $Q_{\alpha }$

The rational function $Q_{\alpha }$ will arise from the equilibrium problem associated with the varying weight $\frac{(z+1)^N(z+\alpha )^N}{z^{2N}}$ that we will analyze in Sect. 4 below. Here we state the formulas that come out of this analysis and we refer to Sect. 4 for motivation why indeed $Q_{\alpha }$ is relevant to our problem. The definition of $Q_{\alpha }$ is different for the two cases $\alpha \le \frac{1}{9}$ and $\alpha \ge \frac{1}{9}$ and this reflects the phase transition at $\alpha = \frac{1}{9}$.

#### Definition 2.1

For each $0 \le \alpha \le 1$, we define two complex numbers $z_\pm (\alpha )$ and a rational function $Q_\alpha$ as follows:

- For $\frac{1}{9} \le \alpha \le 1$, we let
  2.4 $$\begin{aligned} z_{\pm }(\alpha ) = -\frac{3 - 2 \sqrt{\alpha } + 3\alpha }{8} \pm \frac{3i \left( 1+\sqrt{\alpha }\right) }{8} \sqrt{\left( 1- \tfrac{\sqrt{\alpha }}{3}\right) \left( 3 \sqrt{\alpha }-1\right) } \end{aligned}$$
  and
  2.5 $$\begin{aligned} Q_{\alpha }(z) = \frac{\left( z+\sqrt{\alpha }\right) ^2 (z-z_+(\alpha ))(z-z_-(\alpha ))}{z^2(z+1)^2(z+\alpha )^2}. \end{aligned}$$
- For $0 \le \alpha \le \frac{1}{9}$, we let
  2.6 $$\begin{aligned} z_{\pm }(\alpha ) = - \frac{1+3\alpha }{4} \pm \frac{1}{4} \sqrt{(1-\alpha )(1-9\alpha )} \end{aligned}$$
  and
  2.7 $$\begin{aligned} Q_{\alpha }(z) = \frac{(z-z_+(\alpha ))^2 (z-z_-(\alpha ))^2}{z^2(z+1)^2(z+\alpha )^2}. \end{aligned}$$

Let us comment on how $Q_\alpha$ depends on $\alpha$ and the transition at $\alpha = \frac{1}{9}$. For $\frac{1}{9} \le \alpha \le 1$, it can be checked from (2.4) that $|z_{\pm }(\alpha )| = \sqrt{\alpha }$ and

2.8 $$\begin{aligned} z_{\pm }(\alpha ) = \sqrt{\alpha } e^{\pm i \theta _{\alpha }} \end{aligned}$$

for some angle $\theta _{\alpha }$ which increases from $\frac{2\pi }{3}$ to $\pi$ as $\alpha$ decreases from 1 to $\frac{1}{9}$. For $0 \le \alpha \le \frac{1}{9}$, the numbers $z_{\pm }(\alpha )$ are real and satisfy

$$\begin{aligned} -\frac{1}{2}< z_-(\alpha )< -\sqrt{\alpha }< z_+(\alpha )< -\alpha \quad \text {for } 0< \alpha < \frac{1}{9} \end{aligned}$$

with $$z_-(\alpha ) z_+(\alpha ) = \alpha $$.

For $\frac{1}{9}< \alpha < 1$, the function $Q_{\alpha }$ in (2.5) has one double zero and two simple zeros, whereas for $0< \alpha < \frac{1}{9}$ it has two double zeros on the real line by (2.7). For $\alpha = \frac{1}{9}$ both (2.4) and (2.6) yield $z_+(\alpha ) = z_-(\alpha ) = - \frac{1}{3}$, and both (2.5) and (2.7) yield

$$\begin{aligned} Q_{\alpha }(z) = \frac{(z+ \frac{1}{3})^4}{z^2(z+1)^2(z+\frac{1}{9})^2} \qquad \text { for } \alpha = \frac{1}{9}, \end{aligned}$$

which has a fourth order zero at $-\frac{1}{3}$. For $\alpha = 1$, the formulas (2.4) and (2.5) reduce to

2.9 $$\begin{aligned} Q_{\alpha }(z) = \frac{z^2 + z+1}{z^2(z+1)^2} \qquad \text { for } \alpha = 1, \end{aligned}$$

and $$z_{\pm }(1) = -\frac{1}{2} \pm \frac{\sqrt{3}}{2} i = e^{\pm \frac{2\pi i}{3}}$$.

The function $Q_\alpha$ plays an important role in the asymptotic study of the orthogonal polynomials. The *g*-function that is used in the normalization of the RH problem for the orthogonal polynomials will be constructed in terms of $Q_\alpha$ as

2.10 $$\begin{aligned} g(z) = \frac{1}{\pi i} \int _{\Sigma _0} \log (z-s) Q_{\alpha }^{1/2}(s) ds \end{aligned}$$

with $\Sigma _0 = \{ \sqrt{\alpha } e^{it} \mid -\theta _{\alpha } \le t \le \theta _{\alpha } \}$ and $\theta _{\alpha } = \arg z_+(\alpha ) \in [\frac{2\pi }{3},\pi ]$. See Definition 4.2 below for the precise definition of the branches of the logarithm and the square root in (2.10).

The following definition is central for the saddle point analysis of the double integral in (1.7).

#### Definition 2.2

For each $0< \alpha \le 1$ and $(\xi , \eta ) \in {\mathcal {H}}$, we define $\Xi _{\alpha }(z) = \Xi _{\alpha }(z;\xi ,\eta )$ as any solution of the equation

2.11 $$\begin{aligned} \left( \Xi _{\alpha }(z)- \frac{\xi }{2} \left( \frac{1}{z+1}+ \frac{1 }{z+\alpha }\right) +\frac{\eta }{z} \right) ^2= Q_\alpha (z). \end{aligned}$$

**Fig. 4:**
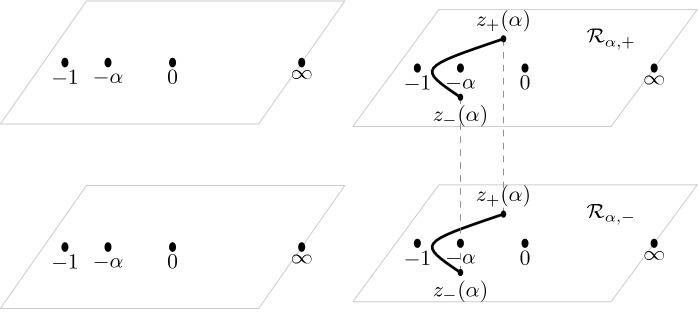
On the right, the two-sheeted Riemann surface for the high temperature case $\frac{1}{9} < \alpha \le 1$ is displayed. The function $\Xi _{\alpha }$ is meromorphic on the Riemann surface with simple poles at the indicated points $-\,1$, $-\,\alpha$, 0 on both sheets and a simple zero at both points at $\infty$. In the low temperature case $0< \alpha < \frac{1}{9}$, the cuts from $z_+(\alpha )$ to $z_-(\alpha )$ disappear and the surface decouples, resulting in the picture that is displayed at the left

In the low temperature regime $0< \alpha < \frac{1}{9}$, we see from (2.7) that $Q_{\alpha }$ is the square of a rational function. This means that (2.11) factorizes and $\Xi _{\alpha }$ decouples into two rational functions with poles at $-1,-\alpha ,0$ and a zero at $\infty$. This in turn implies that we obtain two well-defined rational functions $\Xi _{\alpha ,\pm }$ from (2.11):

2.12 $$\begin{aligned} \begin{aligned} \Xi _{\alpha ,\pm }(z)&= \pm \left( Q_\alpha (z)\right) ^{\frac{1}{2}}+\frac{\xi }{2}\left( \frac{1 }{z+1}+\frac{1 }{z+\alpha }\right) -\frac{\eta }{z} \\&= \pm \frac{(z-z_+(\alpha ))(z-z_-(\alpha ))}{z(z+1)(z+\alpha )} + \frac{\xi }{2}\left( \frac{1 }{z+1}+\frac{1 }{z+\alpha }\right) -\frac{\eta }{z}. \end{aligned} \end{aligned}$$

$\Xi _{\alpha }$ then is a meromorphic function defined on the Riemann surface ${\mathcal {R}}_{\alpha }$ associated with the equation $w^2=(z-z_+(\alpha ))(z-z_-(\alpha ))$. It has two sheets ${\mathcal {R}}_{\alpha ,\pm }$, that are connected by a cut from $z_+(\alpha )$ to $z_-(\alpha )$ that we choose as

$$\begin{aligned} {\mathcal {C}} = \{(w,z) \in {\mathcal {R}}_{\alpha } \mid |z| = \sqrt{\alpha }, \, \theta _{\alpha } \le |\arg z| \le \pi \}, \end{aligned}$$

where we recall from (2.8) that $\theta _{\alpha } = \arg z_+(\alpha ) = - \arg z_-(\alpha )$. We take $w = ((z-z_+)(z-z_-))^{1/2}$ with the branch of the square root that behaves like *z* as $z \rightarrow \infty$ on the first sheet ${\mathcal {R}}_{\alpha ,+}$ and that behaves like $-z$ as $z \rightarrow \infty$ on the second sheet.

Accordingly we have two branches of $\Xi _{\alpha }$,

2.13 $$\begin{aligned} \Xi _{\alpha ,\pm }(z)&= \pm Q_\alpha (z)^{1/2} + \frac{\xi }{2} \left( \frac{1}{z+1}+\frac{1}{z+\alpha }\right) -\frac{\eta }{z},\nonumber \\&= \frac{(z+\sqrt{\alpha })w}{z(z+1)(z+\alpha )} + \frac{\xi }{2} \left( \frac{1}{z+1}+\frac{1}{z+\alpha }\right) -\frac{\eta }{z}, \quad (w, z) \in {\mathcal {R}}_{\alpha , \pm }, \end{aligned}$$

see also Fig. 4. The function $\Xi _{\alpha }$ is meromorphic on the Riemann surface with simple poles at $-1$, $-\alpha$, 0 on both sheets and a simple zero at both points at $\infty$. The four remaining zeros will be the saddle points for the double contour integral.

### Saddle points and the liquid region

We next describe the liquid region for general $0< \alpha \le 1$. A reader acquainted with the asymptotic analysis of similar models for which the kernel can be represented in terms of double integral formulas, will recall that the liquid region in such cases is defined in terms of the saddle points of a phase function occurring in the integrand (see for example [12, 32, 63, 66]). In the present situation, the function $\Xi _{\alpha }$ from (2.12), (2.13) plays the role of the derivative of the phase function, which now turns out to be multivalued. The saddle points are the zeros of $\Xi _{\alpha }$. As was the case in previous works, we are interested in the particular saddle with strictly positive imaginary part (if it exists).

#### Proposition 2.3

Let $0< \alpha \le 1$ and $(\xi , \eta ) \in {\mathcal {H}}$. Then there exists at most one solution $z=s(\xi , \eta ; \alpha )$ to $\Xi _{\alpha }(z;\xi ,\eta )=0$ in ${\mathbb {C}}^+=\{z \in {\mathbb {C}} \mid \mathop {\mathrm {Im}}z>0\}$.

The proof of Proposition 2.3 will be given in Sect. 3. With this result at hand, we define the map $(\xi ,\eta ) \mapsto s(\xi ,\eta ;\alpha )$.

#### Definition 2.4

Let $0 < \alpha \le 1$. We define the **liquid** region ${\mathcal {L}}_\alpha \subset {\mathcal {H}}$ by

$$\begin{aligned} {\mathcal {L}}_\alpha = \left\{ (\xi ,\eta ) \in \mathcal {H} \mid \exists z = s(\xi , \eta ; \alpha ) \in \mathbb {C}^+ : \Xi _{\alpha }(z;\xi ,\eta )=0 \right\} \end{aligned}$$

and the map $s: {\mathcal {L}}_\alpha \rightarrow \mathbb {C}^+$ by $(\xi ,\eta ) \mapsto s(\xi ,\eta ; \alpha )$.

### Main result

**Fig. 5:**
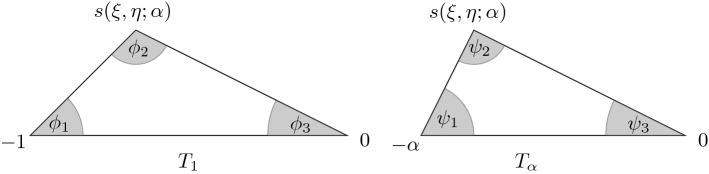
The triangles $T_1$ and $T_\alpha$

For a given $(\xi ,\eta ) \in {\mathcal {L}}_{\alpha }$ with $s = s(\xi ,\eta ;\alpha )$, let $T_1$ and $T_\alpha$ denote the triangles in $\mathbb {C}$ with vertex sets $\{-1,0, s\}$ and $\{-\alpha , 0,s\}$, respectively. As indicated in Fig. 5, the angles of $T_1$ and $T_\alpha$ are denoted by $\{\phi _1,\phi _2, \phi _3\}$ and $\{\psi _1,\psi _2, \psi _3\}$, respectively. Note that $\phi _3=\psi _3$ for any $\alpha$, but $\phi _j=\psi _j$ for $j = 1,2$ if and only if $\alpha =1$. The following is the main result of the paper.

#### Theorem 2.5

Let $\alpha \in (0, 1]$. Let $x,y\in {\mathbb {N}}$ be varying with *N* such that (2.1) holds with $(\xi ,\eta ) \in {\mathcal {L}}_\alpha$. Let $\phi _j = \phi _j(\xi ,\eta ;\alpha )$, $\psi _j = \psi _{j}(\xi ,\eta ;\alpha )$ for $j=1,2,3$ denote the angles of the triangles as shown in Fig. 5. Then2.142.15and2.16

Theorem 2.5 follows from Proposition 7.7 below, and the proof of this proposition will be given in Sect. 7.

Theorem 2.5 describes the situation in the liquid region $\mathcal {L}_{\alpha }$, but it also explains the behavior at the boundary of $\mathcal {L}_{\alpha }$. For each $(\xi ,\eta ) \in {\mathcal {L}}_\alpha$, both $s(\xi ,\eta ;\alpha )$ and $\overline{s(\xi ,\eta ;\alpha )}$ are simple zeros of $\Xi _{\alpha }$. When the point $(\xi ,\eta )$ approaches the boundary of ${\mathcal {L}}_{\alpha }$, the saddle $s(\xi ,\eta ;\alpha )$ approaches the real line. Thus, at the boundary $\partial {\mathcal {L}}_\alpha$, two zeros of $\Xi _{\alpha }$ collide to form a double zero. Note also that when $s(\xi ,\eta ;\alpha )$ approaches the real line, the triangles $T_1$ and $T_\alpha$ collapse with two of the angles approaching 0 and the third approaching $\pi$. In view of Theorem 2.5, this means that the tiling is frozen at the boundary of $\mathcal {L}_{\alpha }$.

### Structure in the low temperature regime

Let us now discuss the low temperature regime in more detail.

In the low temperature regime, each zero of $\Xi _{\alpha }$ is a zero of one of the functions $\Xi _{\alpha ,+}$ or $\Xi _{\alpha ,-}$ from (2.12). These zeros are easy to find since each of the functions $\Xi _{\alpha ,\pm }$ is as a rational function with a quadratic numerator. Setting the numerators equal to zero leads to the equations

2.17 $$\begin{aligned} (s-z_+)(s-z_-) = \pm \left[ \eta (s+1)(s+\alpha ) - \xi s(s+ \tfrac{1+\alpha }{2}) \right] . \end{aligned}$$

with $z_{\pm } = z_{\pm }(\alpha )$. The equations (2.17) are quadratic in *s* with discriminants $D_{\pm } = D_{\pm }(\xi ,\eta )$ that depend on the coordinates $\xi$ and $\eta$:

2.18 $$\begin{aligned} \begin{aligned} D_+(\xi ,\eta )&= \left( \tfrac{1+3\alpha }{2} - (1+\alpha )(\eta - \tfrac{\xi }{2})\right) ^2 - 4 \alpha (1-\eta )(1+\xi -\eta ), \\ D_-(\xi ,\eta )&= \left( \tfrac{1+3\alpha }{2} + (1+\alpha )(\eta - \tfrac{\xi }{2})\right) ^2 - 4 \alpha (1+\eta )(1-\xi +\eta ) \\&= D_+(-\xi ,-\eta ). \end{aligned} \end{aligned}$$

The equations $D_+(\xi ,\eta ) = 0$, $D_-(\xi ,\eta ) = 0$ represent two ellipses in the $(\xi ,\eta )$-plane. The ellipses are inside the hexagon and each one of them is tangent to the boundary of the hexagon in four points. The two ellipses are disjoint for $0< \alpha < \frac{1}{9}$, and they become tangent at the origin for $\alpha = \frac{1}{9}$.

Since a quadratic equation has two complex conjugate roots if and only if the discriminant is negative, we readily obtain the following proposition

#### Proposition 2.6

For each $0< \alpha < \frac{1}{9}$, the liquid region $\mathcal {L}_\alpha$ is the disjoint union of the two open ellipses ${\mathcal {L}}_\alpha ^{\pm }$ defined by

$$\begin{aligned} {\mathcal {L}}_\alpha ^{\pm }&= \left\{ (\xi ,\eta ) \mid D_{\pm }(\xi ,\eta ) <0\right\} , \end{aligned}$$

with $D_{\pm } = D_{\pm }(\xi ,\eta )$ given by (2.18). Moreover, the restrictions of $(\xi ,\eta ) \mapsto s(\xi ,\eta ;\alpha )$ to ${\mathcal {L}}_\alpha ^{\pm }$ are diffeomorphisms onto ${\mathbb {C}}^+$.

See Sect. 3 for the proof, in particular of the statement about the diffeomorphisms.

**Fig. 6:**
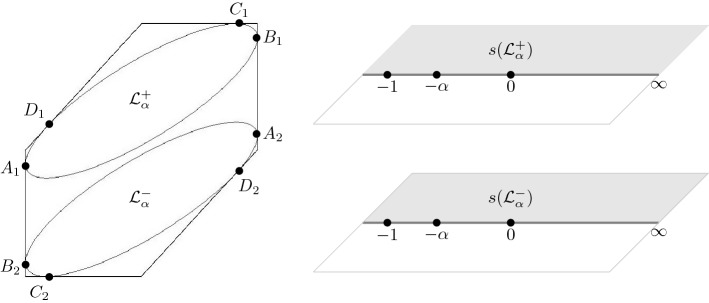
The liquid region (left) and the two disconnected sheets of $\mathcal {R}_{\alpha }$ (right) in the low temperature regime. The diffeomorphism $(\xi ,\eta ) \mapsto s(\xi ,\eta ;\alpha )$ maps the points $A_j$, $B_j$, $C_j$, $D_j$ to $-\,1$, $-\,\alpha$, 0 and $\infty$, respectively

Let us now discuss the behavior of the ellipses near the boundary of the hexagon. The three poles $z=0$, $z=-\alpha$, $z=-1$ of $\Xi _{\alpha ,\pm }(z)$ together with the point at infinity correspond under the map *s* precisely to the points $(\xi ,\eta )$ where the ellipses touch the hexagon, see Fig. 6. A computation gives the following explicit expressions for the points of tangency:

$$\begin{aligned} A_{1,2}&= \pm (-1, -\tfrac{\alpha }{1-\alpha }),&B_{1,2}&= \pm (1, \tfrac{1-2\alpha }{1-\alpha }), \\ C_{1,2}&= \pm (\tfrac{1-\alpha }{1+\alpha } , 1),&D_{1,2}&=\pm (-\tfrac{1-\alpha }{1+\alpha }, \tfrac{2\alpha }{1+\alpha }), \end{aligned}$$

where the $+$ and − signs correspond to the subscripts 1 and 2, respectively.

Given two points *P*, *Q* on one of the ellipses $\partial \mathcal {L}_\alpha ^\pm$, we use the notation $\gamma _{PQ} \subset \partial \mathcal {L}_\alpha ^\pm$ to denote the counterclockwise subarc of the ellipse which starts at *P* and ends at *Q*. As $(\xi , \eta ) \in \mathcal {L}_\alpha$ approaches a point in $\gamma _{B_1C_1} \cup \gamma _{B_2C_2}$, the saddle point $s(\xi ,\eta ;\alpha )$ approaches a point in the interval $(-\alpha ,0)$. Thus, in view of Theorem 2.5, we see that2.19where *x*, *y* and are such that (2.1) holds with $(\xi , \eta ) \in \gamma _{B_1C_1} \cup \gamma _{B_2C_2}$. This behavior extends into the frozen corners near $(\pm 1,\pm 1)$ where only lozenges of this type are present. Similarly, for $(\xi , \eta ) \in \gamma _{C_1D_1} \cup \gamma _{C_2D_2}$,2.20and, for $(\xi , \eta ) \in \gamma _{D_1A_1} \cup \gamma _{D_2A_2}$,2.21The situation is more interesting on the arcs $\gamma _{A_1B_1}$ and $\gamma _{A_2B_2}$. As $(\xi , \eta ) \in \mathcal {L}_\alpha$ approaches one of these arcs, $s(\xi ,\eta ;\alpha )$ approaches the interval $(-1,-\alpha )$. In this limit we have $\phi _2= \pi$ and $\psi _1=\pi$, while all the other angles are zero. This means that at a point (*x*, *y*) near this part of the boundary of the liquid domain, we have2.22i.e., there is an alternating pattern involving two different types of lozenges, as is clearly visible in Fig. 2.

### Structure in the high temperature regime

In the high temperature regime $\frac{1}{9}< \alpha \le 1$, the equation $\Xi _{\alpha }(s;\xi ,\eta )=0$ for the saddle points can be written after squaring as

2.23 $$\begin{aligned} \left( s+ \sqrt{\alpha }\right) ^2 (s-z_+)(s-z_-) = \left( \eta (s+1)(s+\alpha ) - \xi s(s+ \tfrac{1+\alpha }{2}) \right) ^2. \end{aligned}$$

The following proposition (which should be compared with Proposition 2.6) shows that *s* defines a diffeomorphism from the liquid region $\mathcal {L}_\alpha$ to the subset $\mathcal {R}_{\alpha }^+$ of $\mathcal {R}_{\alpha }$ defined by

2.24 $$\begin{aligned} \mathcal {R}_{\alpha }^+ = \{(w,z) \in {\mathcal {R}}_{\alpha } \mid \mathop {\mathrm {Im}}z > 0 \}. \end{aligned}$$

#### Proposition 2.7

For each $\frac{1}{9}< \alpha \le 1$, the map $(\xi ,\eta ) \mapsto s(\xi ,\eta ;\alpha )$ is a diffeomorphism from ${\mathcal {L}}_\alpha$ onto ${\mathcal {R}}_{\alpha }^+$. Moreover, it maps the upper half ${\mathcal {L}}_\alpha ^{+}=\left\{ (\xi ,\eta ) \in {\mathcal {L}}_\alpha \mid \eta >\frac{\xi }{2} \right\}$ onto $\{(w,z) \in {\mathcal {R}}_{\alpha ,+} \mid \mathop {\mathrm {Im}}z>0\}$, and the lower half ${\mathcal {L}}_\alpha ^{-}=\left\{ (\xi ,\eta ) \in {\mathcal {L}}_\alpha \mid \eta <\frac{\xi }{2}\right\}$ onto $\{(w,z) \in {\mathcal {R}}_{\alpha ,-} \mid \mathop {\mathrm {Im}}z>0\}$.

Proposition 2.7 is proved in Sect. 3.

**Fig. 7:**
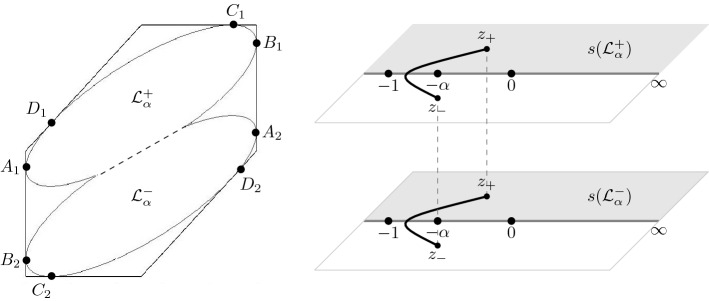
The liquid region (left) and the two sheets of the Riemann surface $\mathcal {R}_{\alpha }$ (right) in the high temperature regime. The diffeomorphism $(\xi ,\eta ) \mapsto s(\xi ,\eta ;\alpha )$ maps the boundary points $A_j$, $B_j$, $C_j$ and $D_j$ to $-\,1$, $-\,\alpha$, 0, and $\infty$, respectively

The boundary $\partial {\mathcal {L}}_\alpha$ of the liquid region is part of the zero set of the discriminant of the quadratic equation (2.23). Since the discriminant is invariant under the map $(\xi ,\eta ) \mapsto (-\xi ,-\eta )$, its zero set is symmetric with respect to the origin. Moreover, the zero set contains the line $\eta = \xi /2$, because (2.23) has a double zero at $s=-\sqrt{\alpha }$ when $\eta = \xi /2$. This line is however not part of the boundary of ${\mathcal {L}}_{\alpha }$.

The discriminant also vanishes at all points $(\xi ,\eta )$ which satisfy an algebraic equation of degree six. The real section of this algebraic curve is a curve inside the hexagon that touches the sides of the hexagon at the points (see Fig. 7)

$$\begin{aligned}&A_{1,2} = \pm \left( -1, -\frac{1}{2} + \frac{3(1-\sqrt{\alpha })}{4(1+\sqrt{\alpha })} \right) ,&B_{1,2} = \pm \left( 1, \frac{1}{2} + \frac{3(1-\sqrt{\alpha })}{4(1+\sqrt{\alpha })} \right) , \\&C_{1,2} = \pm \left( \frac{5}{4} - \frac{3\sqrt{\alpha }}{2(1+\alpha )}, 1 \right) ,&D_{1,2} = \pm \left( -\frac{5}{4} + \frac{3\sqrt{\alpha }}{2(1+\alpha )}, - \frac{1}{4} + \frac{3 \sqrt{\alpha }}{2(1+\alpha )} \right) . \end{aligned}$$

The liquid region is symmetric with respect to the line $\eta = \xi /2$. The cusp points are located at

$$\begin{aligned} E_{1,2}&= \pm (\xi _{cusp}, \eta _{cusp}), \end{aligned}$$

where $$\eta _{cusp} = \xi _{cusp}/2$$ and

$$\begin{aligned} \xi _{cusp} = \sqrt{\frac{5}{2} - \frac{3}{4} \left( \sqrt{\alpha } + \tfrac{1}{\sqrt{\alpha }}\right) } = \sqrt{1- \frac{3}{4} \left( \alpha ^{-1/4} - \alpha ^{1/4}\right) ^2}. \end{aligned}$$

We also have $\eta _{cusp} = \cos \frac{\theta _{\alpha }}{2}$. Note that $\xi _{cusp} = 0$ for $\alpha = 1/9$ and $\xi _{cusp} = 1$ for $\alpha = 1$.

At points on the subarc of the boundary $\partial \mathcal {L}_\alpha$ between $B_j$ and $C_j$ we have (2.19), between $C_j$ and $D_j$ we have (2.20), and between $D_j$ and $A_j$ we have (2.21). This is a consequence of Theorem 2.5 and it is the same as in the low temperature regime. Finally, we have the alternating probabilities (2.22) between $A_1$ and $B_2$, and between $A_2$ and $B_1$.

A notable difference compared with the low temperature regime is that the liquid region in the high temperature regime is connected. As a result, the frozen region with the two types of tiles (sometimes called semi-frozen region) becomes disconnected into two disjoint components.

For $\alpha = 1$, the Eq. (2.23) has a double root at $s=-1$ and two other roots that are the solutions of

$$\begin{aligned} s^2 + s+1 = (\eta (s+1) - \xi s)^2. \end{aligned}$$

The latter two roots coincide if $4 \xi ^2 - 4 \xi \eta + 4 \eta ^2 = 3$ and this is the equation for the ellipse that is tangent to all six sides of the hexagon. The semi-frozen region disappears for $\alpha =1$.

### Local process in the bulk

We chose to present Theorem 2.5 as our main result, but we stress that our method of proof allows us to compute much more complicated asymptotic behaviors (in this sense, our method of proof is the most important contribution of this paper). For instance, with a minor adaptation of the proof of Theorem 2.5 we compute the asymptotic behavior of local correlations in the bulk of the liquid region.

#### Theorem 2.8

Let $0 < \alpha \le 1$. For $j=1,2$, take

2.25 $$\begin{aligned} x_j&= N \xi _N +u_j, \nonumber \\ y_j&= N \eta _N+v_j, \end{aligned}$$

where $\xi _N$ and $\eta _N$ are such that

$$\begin{aligned} \lim _{N\rightarrow \infty }(\xi _N,\eta _N)=(\xi ,\eta )\in {\mathcal {L}}_{\alpha } \end{aligned}$$

and $N\xi _N$ and $N \eta _N$ are integers for every $N\in {\mathbb {N}}$. We will additionally assume, without loss of generality, that $N\xi _N$ is even. The variables $u_1,u_2,v_1$ and $v_2$ are integer valued local variables independent of *N*. Then we have the limit

2.26 $$\begin{aligned} \lim _{N \rightarrow \infty } K_N(x_1,y_1,x_2,y_2) = \frac{1}{2\pi i} \int _{\overline{s}}^s {(z+1)^{\lfloor \frac{u_1}{2}\rfloor -\lfloor \frac{u_2}{2} \rfloor }(z+\alpha )^{\lfloor \frac{u_1+1}{2}\rfloor -\lfloor \frac{u_2+1}{2}\rfloor } }\frac{ dz }{z^{v_1-v_2+1}} \end{aligned}$$

where $s = s(\xi ,\eta ;\alpha )$ and the integration path from $\overline{s}$ to *s* in (2.26) is in ${\mathbb {C}} {\setminus } (-\infty ,0]$ if $u_1 \le u_2$ and in ${\mathbb {C}} {\setminus } [0,\infty )$ if $u_1 >u_2$.

The proof of this theorem is given in Sect. 7.

If $u_1=u_2$ then the integral at the right-hand side of (2.26) can be computed explicitly to be the discrete sine kernel. For general $u_1$ and $u_2$ this is thus a kernel that is an extension of that discrete sine kernel. In fact, it falls into the class of extensions of the discrete sine kernel introduced in [10]. It is to note that the limiting kernel, and thus its associated point processes, depends on $\alpha$. The periodicity in the horizontal direction is thus preserved in the limit.

Theorem 2.8 gives the limiting correlation kernel for the point process of the paths. However, from the path picture one can compute the correlation functions for the different lozenges. For instance, the particle/hole duality tells us that the lozenges  form a determinantal point processes with $1- K_{N}$ as correlation kernel. Under the same assumptions of Theorem 2.8 (but possibly with more than two points) we thus have2.27where $\tilde{K}$ is the kernel at the right-hand side of (2.26).

### Some comments on further asymptotic results

We end this section by commenting on further possible results on the asymptotic behavior of the random tilings.

#### Remark 2.9

(*Frozen regions*). The complement of the liquid region ${\mathcal {L}}_\alpha$ inside the hexagon, is called the frozen region. By definition, in the frozen region there are no solutions of $\Xi _{\alpha }(z;\xi , \eta )=0$ in ${\mathbb {C}}^+$ and all solutions are real. By using a saddle point analysis similar to the one we give in the proof of Theorem 2.5, one can show that this implies exponential decay of the fluctuations. Thus, in the frozen regions the randomness disappears rapidly and the tiling converges to deterministic patterns. In the corners of the hexagon the patterns are simple in the sense that we only have one type of lozenge in each corner. For $\alpha <1$ there are also other frozen regions near the centers of the vertical sides. Also here the randomness decays rapidly, but there are two types of lozenges forming a stair case pattern (as we also see in the degenerate siuation $\alpha =0$ as shown in the left picture in Fig. 3). Frozen regions that have different types of lozenges have appeared in other models. Some examples are [11, 32] (after identifying Gelfand–Tsetlin patterns with lozenge tilings of the half plane). In fact, lozenge tilings of the infinite hexagon (or plane partitions) with an arbitrarily chosen back wall have been studied [17, 60, 61]. Part of this back wall can be a frozen region with more complicated patterns than the staircase pattern of the present paper.

#### Remark 2.10

(*Edge universality*). At the boundary of the liquid region (away from the points where the boundary touches the sides of the hexagon, and, in the high temperature regime, away from the cusp points) we expect Airy behavior. There is a vast amount of literature around this type of universality, and we only refer to [48] for an overview of results.

#### Remark 2.11

(*Turning points*). The turning points are the points where the boundary of the liquid region touches a side of the hexagon. Here we need to distinguish between the turning points that touch the hexagon at a vertical side from the other turning points. In both the low and high temperature regimes (assuming $\alpha < 1$) there are four such points. They separate two frozen regions: one that contains two different types of lozenges, while the other has only one type of lozenges. We expect the local processes there to be the same as the processes that were found in (with a similar weight) in [60]. At the turning points that are not at the vertical sides of the hexagon we expect the GUE minor process [49] to appear.

#### Remark 2.12

(*Cusp points*). In the high temperature limit, the boundary of the liquid region has cusp points. Such cusp points have appeared before in the context of random tilings. It is known that the local limit process near such a cusp point is the Pearcey process [4, 9, 65, 71].

We strongly believe that all the above universal behaviors can be verified using rather straightforward modifcations of the analysis that we present in this paper. More involved are the following remarks:

#### Remark 2.13

(*Tacnode*). At the critical value $\alpha = \frac{1}{9}$ there is a transition from the low to high temperature regimes. The liquid region becomes a union of two ellipses that are tangent at the origin, and the origin is a tacnode. The tacnode process was first characterized in [1] and alternative characterizations were given shortly afterwards in [30, 47]. See also [2, 39]. Preliminary computations indicate that the same tacnode process appears, but we will return to this in a forthcoming paper.

#### Remark 2.14

(*Height fluctuations*). Another interesting feature of random tilings are the fluctuation of the height function. It was found in [51] that the limiting height function can be described by the complex Burgers equation. In [51] it is also conjectured that the fluctuations are described by the Gaussian Free Field. There is by now a long list of random tiling models where this conjecture has been verified, and we only mention [12, 19–21, 32, 33, 67]. This turns out to be a very robust universality. Also in the model considered in this paper, we expect the Gaussian Free Field to appear, but with an interesting transition from the low to high temperature regimes. In the low temperature regime, the correlations between the different ellipses are expected to converge to zero exponentially and we expect to obtain two independent Gaussian Free Fields (in the appropriate coordinates), whilst we have only one Gaussian Free Field in the high temperature regime. It is natural to ask how these two fields merge to one in the transition from the low to high temperature regime. We plan to answer this question in a forthcoming paper.

### Overview of the rest of the paper

In the next section we first prove Propositions 2.3, 2.6 and 2.7.

The rest of the paper is devoted to the proof of Theorem 2.5. It is an asymptotic analysis of the double integral in (1.7) for $K_N(x,y,x,y)$ and for related double integrals that give the probabilities for each of the three lozenges. These double integrals are presented in Theorem 7.1 below.

The asymptotic analysis has two main parts. In the first part we analyze the orthogonal polynomials and their reproducing kernel $R_N(w,z)$ in the large *N* limit. The orthogonal polynomials are characterized by a RH problem that is essentially due to Fokas, Its and Kitaev [40]. This is recalled in Sect. 5.2. The reproducing kernel has a convenient formulation in terms of the solution of the RH problem, see Proposition 5.3. For the asymptotic analysis we use the Deift–Zhou steepest descent method for RH problems. A main ingredient for the analysis is the *g*-function, which in the present context is associated with an equilibrium measure on a contour in the complex plane.

This equilibrium measure is discussed in detail in Sect. 4. The transition at $\alpha = \frac{1}{9}$ is visible in the equilibrium measure since for $\frac{1}{9} < \alpha \le 1$ the equilibrium measure is supported on a circular arc in the complex plane, while for $0 < \alpha \le \frac{1}{9}$ it is supported on a full circle. We are able to give explicit formulas for the equilibrium measure, see Definition 4.2.

The steepest descent analysis of the RH problem is done in Sect. 5. We do not need strong asymptotics of the reproducing kernel $R_N$, it suffices to have a uniform bound on ${\mathcal {R}}_N(w,z) e^{N(g(w)-g(z))}$ (this is in Corollary 5.6) where ${\mathcal {R}}_N(w,z)$ is a function related to the reproducing kernel, and which is given by (5.8).

The second part of the asymptotic analysis is a saddle point analysis of the double integrals like the one in (1.7). The saddle points depend on the asymptotic location $(\xi ,\eta )$ in the hexagon. We focus on the lower left part of the liquid region which corresponds to $\eta \le \frac{\xi }{2} \le 0$. Then the saddle point $s = s(\xi ,\eta ;\alpha )$ is the zero of the derivative of a function $\Phi _{\alpha }$ that is introduced in Sect. 6.1. We want to move the contours in the double integrals to contours $\gamma _z$ and $\gamma _w$ passing through the saddles *s* and $\overline{s}$, and such that

$$\begin{aligned} \mathop {\mathrm {Re}}\Phi _{\alpha }(w)> \mathop {\mathrm {Re}}\Phi _{\alpha }(s) > \mathop {\mathrm {Re}}\Phi _{\alpha }(z) \end{aligned}$$

whenever $w \in \gamma _{w} {\setminus } \{s, \overline{s}\}$ and $z \in \gamma _z {\setminus } \{s, \overline{s}\}$. To be able to do the deformation we need an analysis of the critical level set $\mathop {\mathrm {Re}}\Phi _{\alpha }(z) = \mathop {\mathrm {Re}}\Phi _{\alpha }(s)$ of $\mathop {\mathrm {Re}}\Phi _{\alpha }$ passing through the saddle. This is done in Sect. 6.2.

The actual deformation and splitting of contours is done in Sect. 7. It turns out that the limiting probabilities in (2.14), (2.15), (2.16) come from residue contributions that arise from pole crossings during the deformations of contours. The remaining double contour integrals are then estimated and we only need they tend to zero as $N \rightarrow \infty$. The details of the deformations are different for the low and high temperature regimes.

## Proofs of Propositions 2.3, 2.6 and 2.7

In this section we prove Propositions 2.3, 2.6 and 2.7. We consider the low and high temperature regimes separately.

### The low temperature regime

Since the saddle point equation $\Xi _{\alpha }(s;\xi ,\eta )=0$ reduces to the two quadratic equations (2.17) in the low temperature regime $0< \alpha < \frac{1}{9}$, and also in the critical regime $\alpha = \frac{1}{9}$, Proposition  2.3 is straightforward to prove in this regime.

*Proof of Proposition* 2.3*for*$0< \alpha \le \frac{1}{9}$

Any solution to $\Xi _{\alpha }(s;\xi ,\eta )=0$ is a solution to one of the quadratic equations in (2.17). The discriminants for these quadratic equations are given in (2.18). If, and only if, one of the discriminants is negative, then the corresponding quadratic equation has a zero in ${\mathbb {C}}^+$. Since the discriminants cannot be simultaneously negative, the statement follows. $\square$

#### Proof of Proposition 2.6

It is clear from the discussion preceding Proposition 2.6 that ${\mathcal {L}}_\alpha = {\mathcal {L}}_{\alpha }^{+}\cup {\mathcal {L}}_{\alpha }^{-}$. It is therefore enough to show that the restrictions of $(\xi ,\eta ) \mapsto s(\xi ,\eta ;\alpha )$ to ${\mathcal {L}}_{\alpha }^{\pm }$ are diffeomorphisms onto $\mathbb {C}^+$.

We will show that for each *s* with $\mathop {\mathrm {Im}}s>0$, there are unique points $(\xi _+,\eta _+) \in {\mathcal {L}}_{\alpha }^{+}$ and $(\xi _-,\eta _-) \in {\mathcal {L}}_{\alpha }^{-}$ such that $s= s(\xi _+,\eta _+)= s(\xi _-,\eta _-)$. We rewrite (2.17) as

3.1 $$\begin{aligned} \left( - \frac{s}{2(s+1)} - \frac{s}{2(s+\alpha )} \right) \xi + \eta = \pm \frac{(s-z_+)(s-z_-)}{(s+1)(s+ \alpha )}. \end{aligned}$$

Since $\xi$ and $\eta$ are real, we obtain the following two real equations by taking the real and imaginary parts of (3.1):

3.2 $$\begin{aligned} \begin{pmatrix} \mathop {\mathrm {Re}}\left( - \frac{s}{2(s+1)} - \frac{s}{2(s+\alpha )} \right) &{} 1 \\ \mathop {\mathrm {Im}}\left( - \frac{s}{2(s+1)} - \frac{s}{2(s+\alpha )} \right) &{} 0 \end{pmatrix} \begin{pmatrix} \xi \\ \eta \end{pmatrix} = \pm \begin{pmatrix} \mathop {\mathrm {Re}}\frac{(s-z_+)(s-z_-)}{(s+1)(s+ \alpha )} \\ \mathop {\mathrm {Im}}\frac{(s-z_+)(s-z_-)}{(s+1)(s+ \alpha )} \end{pmatrix}. \end{aligned}$$

We readily see that

3.3 $$\begin{aligned} \mathop {\mathrm {Im}}\left( - \frac{s}{2(s+1)} - \frac{s}{2(s+\alpha )} \right) = \mathop {\mathrm {Im}}\left( -1 + \frac{1}{2(s+1)} + \frac{\alpha }{2(s+\alpha )} \right) < 0, \end{aligned}$$

for $s\in {\mathbb {C}}^+$. Hence the $2 \times 2$ matrix on the left-hand side of (3.2) is invertible whenever $\mathop {\mathrm {Im}}s > 0$. It follows that given $s \in {\mathbb {C}}^+$ we can recover $\xi _\pm$ and $\eta _\pm$ uniquely by

3.4 $$\begin{aligned} \begin{pmatrix} \xi \\ \eta \end{pmatrix} = \pm \begin{pmatrix} \mathop {\mathrm {Re}}\left( - \frac{s}{2(s+1)} - \frac{s}{2(s+\alpha )} \right) &{} 1 \\ \mathop {\mathrm {Im}}\left( - \frac{s}{2(s+1)} - \frac{s}{2(s+\alpha )} \right) &{} 0 \end{pmatrix}^{-1} \begin{pmatrix} \mathop {\mathrm {Re}}\frac{(s-z_+)(s-z_-)}{(s+1)(s+ \alpha )} \\ \mathop {\mathrm {Im}}\frac{(s-z_+)(s-z_-)}{(s+1)(s+ \alpha )} \end{pmatrix}. \end{aligned}$$

This proves that the restrictions of *s* to ${\mathcal {L}}_\alpha ^{\pm }$ are bijections onto ${\mathbb {C}}^+$. The differentiability is also clear, and thus we have proved the statement. $\quad \square$

### The high temperature regime

We now consider the high temperature regime and thus assume $\frac{1}{9} < \alpha \le 1$. We start by defining the polynomial $\Pi _{\alpha }$ by

3.5 $$\begin{aligned} \Pi _{\alpha }(s) = \left( s+ \sqrt{\alpha }\right) ^2 (s-z_+)(s-z_-) - \left( \eta (s+1)(s+\alpha )- \xi s(s+ \tfrac{1+\alpha }{2}) \right) ^2. \end{aligned}$$

By (2.23), the zero set of $\Pi _{\alpha }$ is the image of the zero set of $\Xi _{\alpha }$ under the natural projection $\mathcal {R}_{\alpha } \rightarrow \mathbb {C}$, $(w,z) \mapsto z$.

#### Lemma 3.1

Let $(\xi , \eta ) \in {\mathcal {H}}^{\mathrm {o}}$ (interior of the hexagon ${\mathcal {H}}$) and $\frac{1}{9}< \alpha <1$.

- The leading coefficient of $\Pi _{\alpha }$ is $1- (\eta -\xi )^2 > 0$.
- $$\Pi _{\alpha }(0) = \alpha ^2 (1-\eta ^2) > 0 $$.
- $$\Pi _{\alpha }(-\alpha ) = \frac{\alpha ^2(1-\alpha )^2}{4} (1- \xi ^2) > 0$$.
- $$\Pi _{\alpha }(-\sqrt{\alpha }) = - \alpha (1-\sqrt{\alpha })^4 (\frac{\xi }{2} - \eta )^2 \le 0$$.
- $$\Pi _{\alpha }(-1) = \frac{(1-\alpha )^2}{4} (1-\xi ^2) > 0$$.

#### Proof

These are all simple calculations based on (3.5). The inequalities hold since $-1< \xi < 1$, $-1< \eta < 1$ and $-1< \eta - \xi < 1$ for $(\xi , \eta ) \in {\mathcal {H}}^{\mathrm {o}}$. $\square$

#### Corollary 3.2

Let $(\xi , \eta ) \in {\mathcal {H}}^{\mathrm {o}}$ and $\frac{1}{9}< \alpha <1$. If $\eta = \xi /2$ then $\Pi _{\alpha }(s)$ has a double zero of at $s=-\sqrt{\alpha }$. If $\eta \ne \xi /2$ then $\Pi _{\alpha }(s)$ has at least one zero in $(-1,-\sqrt{\alpha })$ and at least one zero in $(-\sqrt{\alpha },-\alpha )$.

#### Proof

If $\eta \ne \xi /2$ then, by parts (c), (d), and (e) of Lemma 3.1, $\Pi _{\alpha }$ has a sign change, and therefore a zero, in each of the intervals $(-1,-\sqrt{\alpha })$ and $(-\sqrt{\alpha },-\alpha )$. For $\eta = \xi /2$, $\Pi _{\alpha }$ has a zero at $-\sqrt{\alpha }$ by part (d), and in fact

3.6 $$\begin{aligned} \Pi _{\alpha }(s) = (s+\sqrt{\alpha })^2 \left[ (s-z_+)(s-z_-) - \eta ^2 (s-\sqrt{\alpha })^2 \right] \quad \text {if } \eta = \xi /2, \end{aligned}$$

as can be checked from (3.5). Hence $s=-\sqrt{\alpha }$ is a double zero if $\eta = \xi /2$. $\square$

We now give the proof of Proposition 2.3 in the high temperature regime.

#### Proof of Proposition 2.3

for $\frac{1}{9} < \alpha \le 1$. From Corollary 3.2 it follows in particular that there are at least two zeros of $\Pi _{\alpha }$ in $(-1,-\alpha )$ in case $\alpha < 1$. The remaining two zeros can also be real (frozen phase), or be a pair of complex conjugate non-real zeros (liquid phase). There is at most one complex conjugate pair of non-real zeros, and thus at most one zero with strictly positive imaginary part. By continuity this last fact also holds for $\alpha = 1$. This proves Proposition 2.3 in the high temperature regime. $\square$

#### Proof of Proposition 2.7

The proof is similar to the proof of Proposition 2.6. If $s=s(\xi ,\eta ;\alpha )$ with $(\xi ,\eta )\in {\mathcal {L}}_\alpha$ then

$$\begin{aligned} \left( - \frac{s}{2(s+1)} - \frac{s}{2(s+\alpha )} \right) \xi + \eta = \pm s Q_{\alpha }(s)^{1/2}, \end{aligned}$$

see (2.5) and (2.23). As in the proof of Proposition 2.6, we obtain two real equations by considering the real and imaginary parts. It follows that given $s \in {\mathcal {R}}_{\alpha }^+$, where $\mathcal {R}_{\alpha }^+$ denotes the subset of $\mathcal {R}_{\alpha }$ defined in (2.24), we recover $\xi$ and $\eta$ from

3.7 $$\begin{aligned} \begin{pmatrix} \xi \\ \eta \end{pmatrix} = \begin{pmatrix} \mathop {\mathrm {Re}}\left( - \frac{s}{2(s+1)} - \frac{s}{2(s+\alpha )} \right) &{} 1 \\ \mathop {\mathrm {Im}}\left( - \frac{s}{2(s+1)} - \frac{s}{2(s+\alpha )} \right) &{} 0 \end{pmatrix}^{-1} \begin{pmatrix} \mathop {\mathrm {Re}}\left( s Q_{\alpha }(s)^{1/2} \right) \\ \mathop {\mathrm {Im}}\left( s Q_{\alpha }(s)^{1/2} \right) \end{pmatrix}, \end{aligned}$$

where the choice of square root in $Q_{\alpha }(s)^{1/2}$ is dictated by the location of *s* on the Riemann surface (different sign on different sheets).

This shows that $(\xi ,\eta ) \mapsto s(\xi ,\eta ;\alpha )$ is a bijection from ${\mathcal {L}}_{\alpha }$ to ${\mathcal {R}}_{\alpha }^+$. It is clearly also differentiable (but not analytic!) and therefore it is a diffeomorphism. It also extends continuously to the boundary of ${\mathcal {L}}_{\alpha }$ mapping for example $A_{1,2}$ to $-1$, $B_{1,2}$ to $-\alpha$, $C_{1,2}$ to 0, $D_{1,2}$ to $\infty$, and $E_{1,2}$ to $-\sqrt{\alpha }$, where the points with subscript 1 are mapped to the first sheet and points with subscript 2 to the second sheet, see also Fig. 7.

We finally prove that the line segment $\{ (\xi , \xi /2) \mid - \xi _{cusp}< \xi < \xi _{cusp} \}$ is mapped bijectively onto ${\mathcal {C}}^+ = {\mathcal {C}} \cap {\mathcal {R}}_{\alpha }^+$ where (0, 0) is mapped to the branch point $z_+$ and $\pm (\xi _{cusp}, \xi _{cusp}/2)$ is mapped to $z = -\sqrt{\alpha }$ with opposite *w* values $w = \pm 2 \alpha (1+\cos \theta _{\alpha })$.

For $\eta = \xi /2$, we see from (3.6) that $\Pi _{\alpha }(s)$ has a double zero at $-\sqrt{\alpha }$ while the two remaining zeros satisfy

$$\begin{aligned} (s-z_+)(s-z_-) - \eta ^2(s-\sqrt{\alpha })^2 = 0 \end{aligned}$$

which is also

$$\begin{aligned} (1-\eta ^2) (s^2 + \alpha ) + (-2 \cos \theta _{\alpha }+ 2 \eta ^2)\sqrt{\alpha } s = 0 \end{aligned}$$

since $$z_+z_-=\alpha $$ and $$z_+ + z_- = 2 \sqrt{\alpha } \cos \theta _{\alpha }$$.

Suppose $\eta \in [0, \eta _{cusp}]$. Since $\eta _{cusp} = \cos \frac{\theta _{\alpha }}{2}$, we can write $\eta = \cos \frac{\theta }{2}$ with $\theta _{\alpha } \le \theta \le \pi$. There is a unique $\psi \in [\theta _{\alpha }, \pi ]$ with

$$\begin{aligned} \sin \frac{\psi }{2} \sin \frac{\theta }{2} = \sin \frac{\theta _{\alpha }}{2} \end{aligned}$$

and with the aid of trigonometric identities one can show that $s = \sqrt{\alpha } e^{i \psi }$ is a zero of $\Pi _{\alpha }(s)$. If $\eta$ increases from 0 to $\eta _{cusp}$, then $\theta$ decreases from $\pi$ to $\theta _{\alpha }$, and $\psi$ increases from $\theta _{\alpha }$ to $\pi$. It follows that *s* moves along the circle with radius $\sqrt{\alpha }$ from $z_+$ to $-\sqrt{\alpha }$, that is, it moves along one side of the cut ${\mathcal {C}}$ on the Riemann surface. By symmetry, if $\eta$ decreases from 0 to $-\eta _{cusp}$ then the saddle moves along the same circle but on the other side of ${\mathcal {C}}$. $\square$

## Equilibrium Measure and *g*-Function

### Preliminaries

The orthogonality (1.2) does not depend on the specific choice of contour $\gamma$. By analyticity we can deform it to any other contour $\gamma _0$ that goes around 0 once in the positive direction. For the asymptotic analysis we need to select the ‘correct’ contour. The correct contour is typically (but not always...) the contour that attracts the zeros of the orthogonal polynomials as the degree tends to infinity. In (1.2) the orthogonality weight

$$\begin{aligned} e^{-NV(z)} = \frac{(z+1)^N(z+\alpha )^N}{z^{2N}} \end{aligned}$$

varies with *N*, where we put

4.1 $$\begin{aligned} V(z) = V_{\alpha }(z) = 2\log (z) - \log (z+1) - \log (z+ \alpha ). \end{aligned}$$

Such problems were studied in approximation theory where *V* is referred to as an external field [70]. Since the works of Stahl [69] and Gonchar-Rakhmanov [42] it is known that the zeros tend to a contour with a certain symmetry property for the logarithmic potential of its equilibrium measure. Such contours are now called *S*-contours. Later, Rakhmanov [68] made a systematic study of a max-min characterization of *S*-contours, and with Martínez-Finkelshtein [58] introduced the notion of a critical measure and identified the *S*-contours as trajectories of quadratic differentials. See [54, 59] for further developments and historical remarks.

For $\alpha = 1$ the external field (4.1) has only two logarithmic singularities and in such a case the orthogonal polynomials can be written in terms of classical Jacobi polynomials. Indeed, the *n*th degree polynomial $p_n$ is a multiple of the Jacobi polynomial

4.2 $$\begin{aligned} P_n^{(-2N,2N)}(2z+1) \end{aligned}$$

in case $\alpha = 1$. The Jacobi polynomial is non-standard, since one of the parameters is negative. The asymptotic zero distribution of Jacobi polynomials with varying non-standard parameters was studied in [53, 56, 57]. The case (4.2) is contained in [57], see also [31], and it is known that the zeros of (4.2) tend to an arc on the unit circle as $n, N \rightarrow \infty$ with $n/N \rightarrow 1$.

### Equilibrium measure

In order to successfully apply the RH steepest descent analysis to the RH problem 5.2, we need a contour $\gamma _0$ going around 0 and a probability measure $\mu _0$ on $\gamma _0$ with a corresponding *g*-function

4.3 $$\begin{aligned} g(z) = \int \log (z-s) d\mu _0(s) \end{aligned}$$

such that, for some constant $\ell \in {\mathbb {C}}$,

4.4 $$\begin{aligned} \mathop {\mathrm {Re}}\left[ g_+(z) + g_-(z) - V(z) + \ell \right]&{\left\{ \begin{array}{ll} = 0, &{} \text { for } z \in {{\,\mathrm{supp}\,}}(\mu _0), \\ \le 0, &{} \text { for } z \in \gamma _0 {\setminus } {{\,\mathrm{supp}\,}}(\mu _0), \end{array}\right. } \end{aligned}$$

4.5 $$\begin{aligned} \mathop {\mathrm {Im}}\left[ g_+(z) + g_-(z) - V(z) \right]&\begin{array}{l} \text { is constant on each connected } \\ \text { component of } {{\,\mathrm{supp}\,}}(\mu _0), \end{array} \end{aligned}$$

with *V* as in (4.1). We call a probability measure $\mu _0$ satisfying (4.3)–(4.5) an *equilibrium measure in the external fieldV*.

For a given $\gamma$ we consider the probability measure $\mu$ on $\gamma$ that minimizes the energy functional

$$\begin{aligned} \iint \log \frac{1}{|s-t|} d\mu (s) d\mu (t) + \mathop {\mathrm {Re}}\int V d\mu \end{aligned}$$

among all probability measures on $\gamma$. By classical results from logarithmic potential theory [70], there is a unique minimizer and it satisfies the conditions (4.4) on the real part of $g_+ + g_- - V$. In order to be an equilibrium measure for *V* (as we defined it) we also need the condition (4.5) on the imaginary part. This condition characterizes *S*-contours.

Indeed, by the Cauchy–Riemann equations the property (4.5) is equivalent to

$$\begin{aligned} \frac{ \partial }{\partial n_+} \left[ U^{\mu _0} + \frac{\mathop {\mathrm {Re}}V}{2} \right] = \frac{\partial }{\partial n_-} \left[ U^{\mu _0} + \frac{\mathop {\mathrm {Re}}V}{2} \right] \end{aligned}$$

on the support $\Sigma _0 = {{\,\mathrm{supp}\,}}(\mu _0)$, where

$$\begin{aligned} U^{\mu _0}(z) = \int \log \frac{1}{|z-s|} d\mu _0(s) \end{aligned}$$

and $\frac{\partial }{\partial n_{\pm }}$ denotes the normal derivatives on $\gamma$. This property is known as the *S*-property of $\Sigma _0$, and $\gamma _0$ is an *S*-contour.

We remark that the equilibrium measure is not necessarily unique. For example, if $V(z) = \log z$ then the normalized Lebesgue measure $d\mu = \frac{ds}{2\pi i s}$ on any circle centered at the origin is an equilibrium measure for *V*. The radius is arbitrary and the equilibrium measure is not unique. This is a more general phenomenon in case the support is a full closed contour.

### Construction of the equilibrium measure

From conditions (4.4)–(4.5) it follows that we are looking for $\mu _0$ such that $g_+ + g_- - V$ is piecewise constant on the support of $\mu _0$ and therefore

$$\begin{aligned} g_+' + g_-' - V' = 0 \quad \text { on } \Sigma _0 = {{\,\mathrm{supp}\,}}(\mu _0). \end{aligned}$$

This means that $(g' - \frac{1}{2} V')_+ = - (g' - \frac{1}{2} V')_-$ and therefore

4.6 $$\begin{aligned} Q(z) = \left[ \int \frac{d\mu _0(s)}{z-s} - \frac{V'(z)}{2} \right] ^2 \end{aligned}$$

is analytic across the support of $\mu _0$. Thus *Q* is an analytic function in the complex plane with singularities determined by the singularities of $V'$. We can furthermore recover $\mu _0$ from *Q*. Indeed with an appropriate branch of the square root,

$$\begin{aligned} \int \frac{d\mu _0(s)}{z-s} = \frac{V'(z)}{2} + Q(z)^{1/2} \end{aligned}$$

and then by the Sokhotski Plemelj formula

4.7 $$\begin{aligned} d\mu _0(s) = \frac{1}{\pi i} Q_-(s)^{1/2} ds. \end{aligned}$$

In our case of interest we have (4.1) and

4.8 $$\begin{aligned} V_{\alpha }'(z) = \frac{2}{z} - \frac{1}{z+1} - \frac{1}{z+\alpha } \end{aligned}$$

is rational with three simple poles. Therefore by (4.6) $Q = Q_{\alpha }$ is a rational function with double poles at $z=0$, $z=-1$, and $z=-\alpha$. We can determine $Q_{\alpha }$ explicitly, and it is given by the formulas in Definition 2.1, see also Sect. 4.6 below. We will prove that the associated measure (4.7) is indeed an equilibrium measure with external field $V_{\alpha }$.

#### Remark 4.1

We recall from Sect. 2.2 that

4.9 $$\begin{aligned} Q_{\alpha }(z)^{1/2} = \frac{(z-z_+)(z-z_-)}{z(z+1)(z+\alpha )}, \qquad \text {if } 0 < \alpha \le \frac{1}{9}, \end{aligned}$$

while for $\frac{1}{9} < \alpha \le 1$ the square root $Q_{\alpha }(z)^{1/2}$ was considered as a function on the first sheet of the Riemann surface ${\mathcal {R}}_{\alpha }$ shown in the right panel of Fig. 4. From now on it will be more convenient to change the branch cut of the Riemann surface from ${\mathcal {C}}$ to

4.10 $$\begin{aligned} \Sigma _0 = \{ \sqrt{\alpha } e^{i t} \mid -\theta _{\alpha } \le t \le \theta _{\alpha } \} \end{aligned}$$

where $\theta _{\alpha } = \arg z_+ = - \arg z_-$. We also modify the definition of $Q_{\alpha }(z)^{1/2}$ so that now

4.11 $$\begin{aligned} Q_{\alpha }(z)^{1/2} = \frac{(z+\sqrt{\alpha }) ((z-z_+)(z-z_-))^{1/2}}{z(z+1)(z+\alpha )}, \quad \text {if } \frac{1}{9} < \alpha \le 1, \end{aligned}$$

is defined and analytic for $z \in {\mathbb {C}} {\setminus } \Sigma _0$ with the square root such that $Q_{\alpha }(z)^{1/2} \sim \frac{1}{z}$ as $z \rightarrow \infty$. The circular arc (4.10) will be the support of the equilibrium measure $\mu _0$.

We let $\gamma _0$ denote the circle of radius $\sqrt{\alpha }$ centered at 0 oriented in the counterclockwise direction.

With (4.9) and (4.11), we define the measure $\mu _0$, the associated *g*-function, and the variational constant $\ell$ as follows.

#### Definition 4.2

(a) If $\frac{1}{9} \le \alpha \le 1$, then we define the measure $\mu _0$ by

4.12 $$\begin{aligned} d\mu _0(s)&= \frac{1}{\pi i} Q_{\alpha ,-}(s)^{1/2} ds \nonumber \\&= \frac{1}{\pi i} \frac{(s+\sqrt{\alpha }) \, ((s-z_+)(s-z_-))^{1/2}_-}{s(s+1)(s+\alpha )} \, ds, \qquad s \in \Sigma _0, \end{aligned}$$

where $\Sigma _0$ is given by (4.10) with counterclockwise orientation, and $Q_{\alpha ,-}(s)^{1/2}$ denotes the limit of $Q_{\alpha }(z)^{1/2}$ as $z \rightarrow s \in \Sigma _0$ with *z* in the exterior of the circle $\gamma _0$. Recall $z_{\pm }= z_{\pm }(\alpha )$ are given by (2.4).

The associated *g*-function is defined by

$$\begin{aligned} g(z) = \int _{\Sigma _0} \log (z-s) d\mu _0(s), \qquad z \in {\mathbb {C}}{\setminus } \left( (-\infty , -\sqrt{\alpha }] \cup \{\sqrt{\alpha } e^{i t} \mid -\pi \le t \le \theta _{\alpha } \} \right) , \end{aligned}$$

where for each $s \in \Sigma _0$, the branch of the logarithm $z \mapsto \log (z-s)$ is taken that is analytic in ${\mathbb {C}}{\setminus } ((-\infty , -\sqrt{\alpha }] \cup \{ \sqrt{\alpha } e^{i t} \mid -\pi \le t \le \arg s\}$ and behaves like $\log (z-s) \sim \log |z| + i \arg (z)$, $-\pi< \arg z < \pi$ as $z \rightarrow \infty$.

(b) If $0 < \alpha \le \frac{1}{9}$, then we define the measure $\mu _0$ by

4.13 $$\begin{aligned} d\mu _0(s)&= \frac{1}{\pi i} Q_{\alpha }(s)^{1/2} ds \nonumber \\&= \frac{1}{\pi i} \frac{(s-z_+)(s-z_-)}{s(s+1)(s+\alpha )} ds, \qquad s \in \Sigma _0, \end{aligned}$$

where $\Sigma _0 = \gamma _0 = {{\,\mathrm{supp}\,}}(\mu _0)$ is the full circle of radius $\sqrt{\alpha }$ oriented in the counterclockwise direction and $z_{\pm }= z_{\pm }(\alpha )$ are given by (2.6).

The associated *g*-function is defined by

$$\begin{aligned} g(z) = \int _{\Sigma _0} \log (z-s) d\mu _0(s), \qquad z \in {\mathbb {C}}{\setminus } \left( (-\infty , -\sqrt{\alpha }] \cup \Sigma _0\right) \end{aligned}$$

where $z \mapsto \log (z-s)$ is defined in the same way as in the high temperature regime.

(c) We define the variational constant $\ell \in {\mathbb {C}}$ by

4.14 $$\begin{aligned} \ell = {\left\{ \begin{array}{ll} -2g_{-}(\sqrt{\alpha }) + V_{\alpha }(\sqrt{\alpha }) - \pi i, &{} \text{ if } 0< \alpha \le \frac{1}{9} \\ - 2g(z_{+}) + V_{\alpha }(z_{+}), &{} \text{ if } \frac{1}{9} < \alpha \le 1 \end{array}\right. }. \end{aligned}$$

The definition (4.14) is such that equality holds in (4.4) at $z = z_{+} \in \Sigma _0$ for $\frac{1}{9}<\alpha \le 1$ and at $z = \sqrt{\alpha }\in \Sigma _{0}$ for $0<\alpha \le \frac{1}{9}$.

For the steepest descent analysis of the RH problem, it is convenient to introduce a function $\phi (z)$ which is a primitive function of $Q_\alpha (z)^{1/2}$ (with appropriate choices of the branch).

#### Definition 4.3

(a) If $\frac{1}{9} < \alpha \le 1$, then $\phi :{\mathbb {C}}{\setminus } ((-\infty ,0] \cup \{\sqrt{\alpha } e^{it} \mid -\pi \le t \le \theta _{\alpha } \}) \rightarrow {\mathbb {C}}$ is defined by

4.15 $$\begin{aligned} \phi (z) = \int _{z_{+}}^z Q_{\alpha }(s)^{1/2} ds, \end{aligned}$$

with $Q_{\alpha }^{1/2}$ given by (4.11), and the integration path from $z_{+}$ to *z* does not intersect $(-\infty ,0] \cup \{\sqrt{\alpha } e^{it} \mid -\pi \le t \le \theta _{\alpha } \}$.

(b) If $0< \alpha < \frac{1}{9}$, then $\phi :{\mathbb {C}}{\setminus } ((-\infty ,0] \cup \Sigma _0) \rightarrow {\mathbb {C}}$ is defined by

4.16 $$\begin{aligned} \phi (z) = {\left\{ \begin{array}{ll} \displaystyle - \frac{\pi i}{2} + \int _{\sqrt{\alpha }}^{z} Q_{\alpha }(s)^{1/2} ds, &{} \text {for } |z| > \sqrt{\alpha }, \\ \displaystyle \frac{\pi i}{2} -\int _{\sqrt{\alpha }}^{z} Q_{\alpha }(s)^{1/2} ds, &{} \text {for } |z| < \sqrt{\alpha }, \end{array}\right. } \end{aligned}$$

with $Q_{\alpha }^{1/2}$ given by (4.9), and the integration path from $\sqrt{\alpha }$ to *z* does not intersect $(-\infty ,0] \cup \Sigma _0$.

The formulas (4.12) and (4.13) define $\mu _0$ as a complex measure on $\Sigma _0$. The fact that it is a probability measure is part of the statement of the following proposition whose proof is given in Sect. 4.5.

#### Proposition 4.4

Let $0 < \alpha \le 1$ and let $\gamma _0$ be the circle of radius $\sqrt{\alpha }$ centered at 0 oriented positively. Then the measure $\mu _0$ defined in (4.12) and (4.13) is a probability measure on $\Sigma _0$ and is an equilibrium measure in the external field $V_{\alpha }$. The functions *g* and $\phi$ are analytic in their domains of definitions and are related by

4.17 $$\begin{aligned} \phi (z) = g(z) - \frac{V_\alpha (z)}{2} + \frac{\ell }{2} \end{aligned}$$

for all *z* in the domain of $\phi$. Moreover,

4.18 $$\begin{aligned} g_+(z) + g_-(z) - V_\alpha (z)&= - \ell , \quad \text { for } z \in \Sigma _0, \end{aligned}$$

4.19 $$\begin{aligned} g_+(z) - g_-(z) - 2\phi _+(z)&= 0, \qquad \text {for } z \in \Sigma _0. \end{aligned}$$

### The zero set of $\mathop {\mathrm {Re}}\phi$

To prepare for the proof of Proposition 4.4 we first present a lemma about the quadratic differential $Q_{\alpha }(z) dz^2$.

A smoothly parametrized curve $z=z(t)$, $t \in [a,b]$, is a *trajectory* of a quadratic differential $Q(z) dz^2$ if $Q(z(t)) z'(t)^2 < 0$ for every $t \in (a,b)$. It is an *orthogonal trajectory* if $Q(z(t)) z'(t)^2 > 0$ for every $t \in (a,b)$. A trajectory or an orthogonal trajectory is *critical* if it contains a zero or a pole of *Q*.

#### Lemma 4.5

- For every $\alpha \in (0,1]$, the curve $\Sigma _0$ is a trajectory of the quadratic differential $Q_{\alpha }(z) dz^2$. If $\alpha \ge \frac{1}{9}$, then it is a critical trajectory passing through the zeros $z_\pm (\alpha )$ of $Q_{\alpha }$.
- For every $\alpha \in (\frac{1}{9},1]$, the complementary arcs on the circle $|z| = \sqrt{\alpha }$, with parametrizations $z(t) = \sqrt{\alpha } e^{it}$, $t \in (\theta _{\alpha }, \pi )$ or $t \in (-\pi , -\theta _{\alpha })$ are critical orthogonal trajectories that connect $z_{\pm }(\alpha )$ with the double zero at $-\sqrt{\alpha }$.

#### Proof

Let $z= z(t) = \sqrt{\alpha } e^{i t}$, so that $z' = i z$. For $\alpha \ge \frac{1}{9}$, we write $z_{\pm } = \sqrt{\alpha } e^{\pm i \theta _{\alpha }}$ with $0 < \theta _{\alpha } \le \pi$, and then by (2.5)

4.20 $$\begin{aligned} Q_\alpha (z) (z')^2&= - \frac{(z+ \sqrt{\alpha })^2 (z-z_+)(z-z_-)}{(z+1)^2(z+\alpha )^2} \nonumber \\&= - \alpha ^2 \frac{(e^{it} + 1)^2 (e^{it}-e^{i\theta _{\alpha }})(e^{it}-e^{-i\theta _{\alpha }})}{(\sqrt{\alpha } e^{it} + 1)^2 (\sqrt{\alpha } e^{it} + \alpha )^2} \nonumber \\&= - 16 \alpha \frac{ \left( \cos \frac{t}{2} \right) ^2 \sin \left( \frac{\theta _{\alpha }-t}{2} \right) \sin \left( \frac{\theta _{\alpha }+t}{2} \right) }{(1+ \alpha + 2\sqrt{\alpha } \cos t)^2}. \end{aligned}$$

This expression is indeed $< 0$ for $-\theta _{\alpha }< t < \theta _{\alpha }$ and $>0$ for $\theta _{\alpha }< t < \pi$ and $-\pi< t < -\theta _{\alpha }$.

For $0< \alpha < \frac{1}{9}$, a similar computation using (2.7) and (2.6) gives

4.21 $$\begin{aligned} Q_\alpha (z) (z')^2&= - \frac{(z- z_+)^2 (z-z_-)^2}{(z+1)^2(z+\alpha )^2} = - \frac{(z^2 + \frac{1+3\alpha }{2} z + \alpha )^2}{(z+1)^2(z+\alpha )^2} = - \frac{ \left( \frac{1+3\alpha }{2} + 2 \sqrt{\alpha } \cos t\right) ^2}{(1+\alpha + 2 \sqrt{\alpha } \cos t)^2}. \end{aligned}$$

Since $0<\alpha < \frac{1}{9}$ we have $\frac{1+3 \alpha }{2} > 2 \sqrt{\alpha }$ and therefore the numerator is always $>0$. Thus $Q_\alpha (z) (z')^2 < 0$ for every $t \in [-\pi , \pi ]$. $\square$

**Fig. 8:**
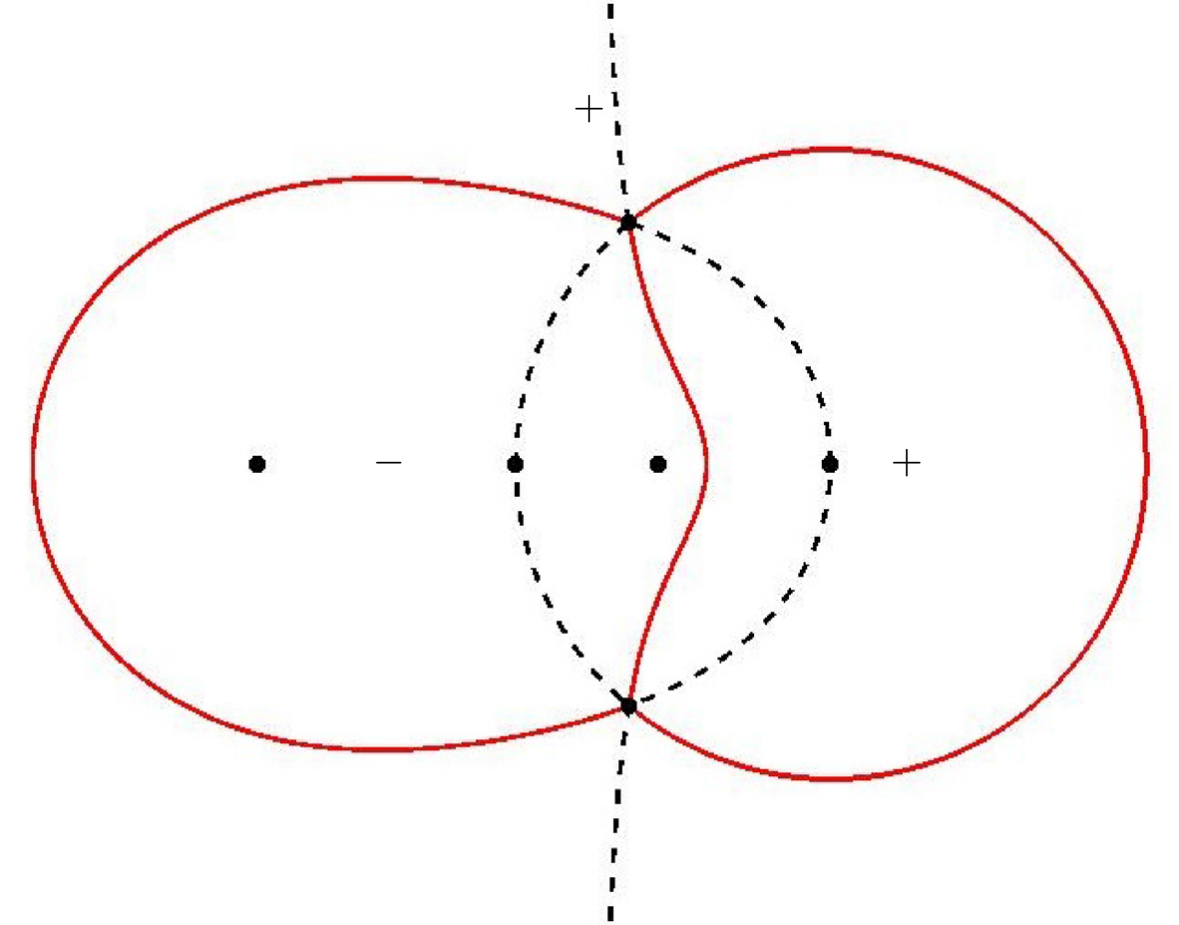
The critical trajectories (in full red lines) and the critical orthogonal trajectories (the dashed black lines) of $Q_{\alpha }$ for $\alpha = 0.3$. The dots are the zeros and poles of $Q_{\alpha }$: $z_{+}$, $z_{-}$, $-\sqrt{\alpha }$, and $-\,1$, $-\,\alpha$, 0. The critical trajectories are level lines $\mathop {\mathrm {Re}}\phi = 0$ and their complement consists of three regions where the sign of $\mathop {\mathrm {Re}}\phi$ is constant, as shown by $+$ and − in the figure

For $\alpha > \frac{1}{9}$ we recall that $z_{\pm }$ are simple zeros of $Q_{\alpha }$. From the local structure of trajectories of a quadratic differential there are three critical trajectories emanating from each of the points $z_{\pm }$. One of these is an arc on the circle $|z|= \sqrt{\alpha }$, as we have seen. The other critical trajectories also connect $z_+$ with $z_-$ and a representative situation is shown in Fig. 8.

The trajectories of the quadratic differential $Q_{\alpha }(z)dz^2$ are level lines of $\mathop {\mathrm {Re}}\phi$, since $\phi$ is a primitive function of $\pm Q_{\alpha }^{1/2}$ as follows from Definition 4.3. The orthogonal trajectories are level lines of $\mathop {\mathrm {Im}}\phi$.

Since $\sqrt{\alpha } \in \Sigma _0$ we in fact have that $\mathop {\mathrm {Re}}\phi = 0$ on $\Sigma _0$ as well as on the other critical trajectories (in the high temperature regime) that are shown in Fig. 8 for $\alpha = 0.3$. The three critical trajectories are boundaries of three regions in the complex plane on which $\mathop {\mathrm {Re}}\phi$ has a constant sign. Namely $\mathop {\mathrm {Re}}\phi < 0$ in the region containing $-1$, and $\mathop {\mathrm {Re}}\phi > 0$ in the region containing 0 and in the unbounded region.

To prove this we introduce

$$\begin{aligned} {\mathcal {N}}_\phi = \{ z \mid \mathop {\mathrm {Re}}\phi (z)=0\}. \end{aligned}$$

**Fig. 9:**
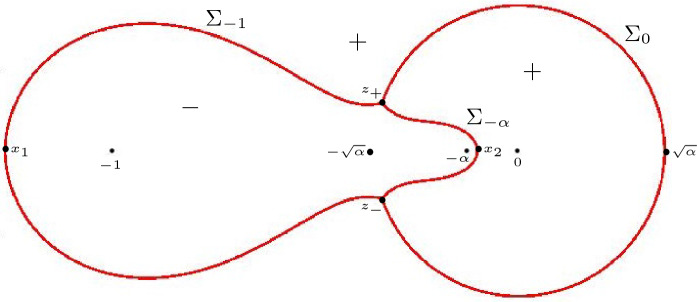
The set $\mathcal {N}_{\phi }=\{z \in \mathbb {C} : \mathop {\mathrm {Re}}\phi (z) = 0\} = \Sigma _{-1} \cup \Sigma _{-\alpha } \cup \Sigma _0$ is shown for $\alpha = \frac{1}{8}$. This set divides $\mathbb {C}$ into three regions, and the sign of $\mathop {\mathrm {Re}}\phi$ is shown in each of these regions

**Fig. 10:**
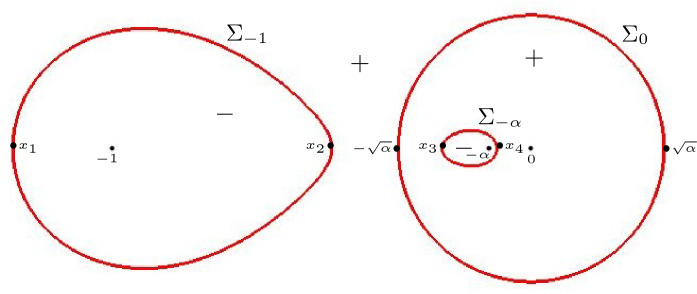
The set $\mathcal {N}_{\phi }=\{z \in \mathbb {C} \mid \mathop {\mathrm {Re}}\phi (z) = 0\} = \Sigma _{-1} \cup \Sigma _{-\alpha } \cup \Sigma _0$ is shown for $\alpha = \frac{1}{10}$. This set divides $\mathbb {C}$ into four regions, and the sign of $\mathop {\mathrm {Re}}\phi$ is shown in each of these regions

Then $\Sigma _0$ is contained in ${\mathcal {N}}_\phi$, but ${\mathcal {N}}_\phi$ also contains other parts, see Figs. 9 and 10 for representative figures in the high and low temperature regimes.

The first thing to observe is that $\mathop {\mathrm {Re}}\phi$ extends to a continuous function on ${\mathbb {C}}$ away from $-1$, $-\alpha$, and 0. Indeed, $Q_{\alpha }^{1/2}$ has simple poles at these three values, and therefore by integration as in definitions (4.15) and (4.16), we find that $\phi$ has logarithmic behavior. However, since the residues of $Q_{\alpha }^{1/2}$ are real, the real part of $\phi$ is single-valued. Thus $\mathop {\mathrm {Re}}\phi$ is continuous on ${\mathbb {C}} {\setminus } \{-1,-\alpha ,0\}$ and harmonic on ${\mathbb {C}} {\setminus } (\Sigma _0 \cup \{-1,-\alpha ,0\})$. We also note

4.22 $$\begin{aligned} \begin{aligned} \phi (z)&= - \log z + {\mathcal {O}}(1) \text { as } z \rightarrow 0,&\lim _{z \rightarrow 0} \mathop {\mathrm {Re}}\phi (z) = + \infty \\ \phi (z)&= \frac{1}{2} \log (z+\alpha ) + {\mathcal {O}}(1) \text { as } z \rightarrow -\alpha ,&\lim _{z \rightarrow -\alpha } \mathop {\mathrm {Re}}\phi (z) = - \infty \\ \phi (z)&= \frac{1}{2} \log (z+1) + {\mathcal {O}}(1) \text { as } z \rightarrow -1,&\lim _{z \rightarrow -1} \mathop {\mathrm {Re}}\phi (z) = - \infty \\ \phi (z)&= \log (z) + {\mathcal {O}}(1) \text { as } z \rightarrow \infty ,&\lim _{z \rightarrow \infty } \mathop {\mathrm {Re}}\phi (z) = + \infty . \end{aligned} \end{aligned}$$

In the high temperature regime the level set ${\mathcal {N}}_{\phi }$ consists of the critical trajectories of the quadratic differential $Q_{\alpha }(z) dz^2$ emanating from $z_+(\alpha )$.

#### Lemma 4.6

Let $\frac{1}{9} < \alpha \le 1$. The set ${\mathcal {N}}_\phi$ consists of three analytic arcs connecting $z_+$ and $z_-$ which we denote by $\Sigma _{-1}$, $\Sigma _{-\alpha }$ and $\Sigma _0$. The arc $\Sigma _{-1}$ intersects the real axis at $x_1 \in (-\infty ,-1)$ and $\Sigma _{-\alpha }$ intersects the real axis at $x_2 \in (-\alpha ,0)$. The arc $\Sigma _0$ is the support of the measure $\mu _0$ and is part of the circle $|z| = \sqrt{\alpha }$.

#### Proof

Because of the local behavior of trajectories of a quadratic differential at a simple zero, there are three trajectories emanating from $z_+$. One of these trajectories is $\Sigma _0$. The other two trajectories have to remain bounded and stay away from the poles $-1$, $-\alpha$, 0 by (4.22). They have to come to the real axis. Indeed, if not, they would have to form a close loop in the upper haf plane and, since $\mathop {\mathrm {Re}}\phi$ is harmonic inside this closed loop, we obtain a contradiction with the maximum/minimum principle for harmonic functions. Therefore, the trajectories come to the real axis and, by symmetry, they continue to the other simple zero $z_- = \overline{z_+}$. The three trajectories enclose two bounded domains and $\mathop {\mathrm {Re}}\phi = 0$ on the boundary of these domains. Again, note that $\mathop {\mathrm {Re}}\phi$ is harmonic in the interior, except at $-1$, $-\alpha$, 0, where it tends to $\pm \infty$, see (4.22). By the maximum/minimum principle of harmonic functions each of the domains should contain at least one of the singularities.

Again by (4.22) there are points $x_1 \in (-\infty ,-1)$ and $x_2 \in (-\alpha ,0)$ with $\mathop {\mathrm {Re}}\phi (x_1) = \mathop {\mathrm {Re}}\phi (x_2) = 0$. Also $\mathop {\mathrm {Re}}\phi (\sqrt{\alpha }) = 0$ and we claim that $x_1, x_2, \sqrt{\alpha }$ are the only points in ${\mathcal {N}}_{\phi } \cap {\mathbb {R}}$.

To see this we recall that $\phi ' = Q_{\alpha }^{1/2}$, with a branch cut along $\Sigma _0$ for the square root. From the formula (4.11) we then see that $\phi '$ changes sign in the five values $-1$, $-\sqrt{\alpha }$, $-\alpha$, 0, and $\sqrt{\alpha } \in \Sigma _0$. Thus $\phi ' > 0$ (and $\mathop {\mathrm {Re}}\phi$ is strictly increasing) on the intervals $(-1,-\sqrt{\alpha })$, $(-\alpha , 0)$, and $(\sqrt{\alpha }, \infty )$, while $\phi ' < 0$ (and $\mathop {\mathrm {Re}}\phi$ is stictly decreasing) on $(-\infty , -1)$, $(-\sqrt{\alpha },-\alpha )$, and $(0, \sqrt{\alpha })$. Since $\mathop {\mathrm {Re}}\phi (\sqrt{\alpha }) = 0$, we conclude that there are no other zeros of $\mathop {\mathrm {Re}}\phi$ in $[0,\infty )$. Also $x_1$ is the only zero in $(-\infty ,-1]$ and $x_2$ is the only zero of $\mathop {\mathrm {Re}}\phi$ in $[-\alpha ,0]$. On the remaining interval $(-1,-\alpha )$, we see that $\mathop {\mathrm {Re}}\phi$ assumes its maximum value at $-\sqrt{\alpha }$. At $-\sqrt{\alpha }$ we have by (4.17)

$$\begin{aligned} \mathop {\mathrm {Re}}\phi = \mathop {\mathrm {Re}}\left( g - \frac{V_{\alpha }}{2} + \frac{\ell }{2}\right) < 0 \end{aligned}$$

where the inequality holds because of the variational inequality (4.4) at $-\sqrt{\alpha } \in \gamma _0 {\setminus } \Sigma _0$, which in the high temperature regime is a strict inequality, see also (4.29). Therefore $\mathop {\mathrm {Re}}\phi$ has no zeros in $(-1,-\alpha )$, and we proved the claim that

$$\begin{aligned} {\mathcal {N}}_{\phi } \cap {\mathbb {R}} = \{x_1, x_2, \sqrt{\alpha } \}. \end{aligned}$$

We conclude that one critical trajectory comes to $x_1$ and another one to $x_2$. This defines the contours $\Sigma _{-1}$ and $\Sigma _{-\alpha }$.

It remains to prove there are no other parts in ${\mathcal {N}}_\phi$. Any potential other part of ${\mathcal {N}}_{\phi }$ cannot intersect the real axis, as we already saw. Then such a part would be a closed contour in the upper or lower half plane and we arrive, again, at a contradiction because of the maximum/minimum principle for harmonic functions. $\square$

The structure of ${\mathcal {N}}_{\phi }$ is different in the low temperature regime, see Fig. 10.

#### Lemma 4.7

Let $0< \alpha < \frac{1}{9}$. The set ${\mathcal {N}}_\phi$ is the disjoint union of three analytic closed curves which we denote by $\Sigma _{-1}$, $\Sigma _{-\alpha }$ and $\Sigma _0$. The closed curve $\Sigma _0$ is the circle of radius $\sqrt{\alpha }$ around 0, as before, and $\Sigma _{-1}$, $\Sigma _{-\alpha }$ are two closed curves lying in the exterior/interior of $\Sigma _0$ and going around $-1$ and $-\alpha$, respectively.

#### Proof

Because of (4.22) the level set ${\mathcal {N}}_\phi$ is bounded and stays away from the poles $-1$, $-\alpha$, and 0 of $Q_{\alpha }$. Since we already know from Lemma 4.5 that $\mathop {\mathrm {Re}}\phi (-\sqrt{\alpha }) = 0$, we infer from (4.16) that the zeros $z_{\pm }$ of $Q_{\alpha }$ are not on ${\mathcal {N}}_{\phi }$. Therefore ${\mathcal {N}}_{\phi }$ does not contain any critical trajectories and hence consists of a finite union of disjoint closed curves. Because of the maximum/minimum principle for harmonic functions each component of $\overline{\mathbb {C}} {\setminus } {\mathcal {N}}_\phi$ contains at least one of the singularities $-1$,$-\alpha$, 0, or $\infty$.

A closer inspection of $\mathop {\mathrm {Re}}\phi (z)$ for $z \in {\mathbb {R}}$ (also based on (4.9), (4.16) and (4.22) reveals that ${\mathcal {N}}_\phi$ has six intersection points with ${\mathbb {R}}$. Two of them are the points $\pm \sqrt{\alpha }$ that belong to $\Sigma _0$. Then we have one point in each of the intervals $(-\infty ,-1)$, $(-1,-\sqrt{\alpha })$, $(-\sqrt{\alpha }, -\alpha )$ and $(-\alpha ,0)$. This shows that there is a closed curve $\Sigma _{-\alpha }$ inside $\Sigma _0$ and a closed curve $\Sigma _{-1}$ outside $\Sigma _0$ as indicated in the statement. $\square$

### Proof of Proposition 4.4

We compute $\int \nolimits _{\Sigma _0} d\mu _0$ by means of a residue calculation. Let us first consider the case $\frac{1}{9} \le \alpha \le 1$. Then by (4.12) and the fact that $Q_{\alpha ,+}(s)^{1/2} = - Q_{\alpha ,-}(s)^{1/2}$ for $s \in \Sigma _0$, we have

4.23 $$\begin{aligned} \int _{\Sigma _0} d\mu _0 = \frac{1}{2\pi i} \oint _{C} Q_{\alpha }(s)^{1/2} ds \end{aligned}$$

where *C* is a closed contour going around $\Sigma _0$ once in the positive direction, and without enclosing any of the poles. Deforming the contour *C* to infinity, we pick up residue contributions at the poles. It is a straightforward calculation to show that

4.24 $$\begin{aligned} \mathop {{{\,\mathrm{Res}\,}}}\limits _{s=0} Q_{\alpha }(s)^{1/2} = -1, \quad \mathop {{{\,\mathrm{Res}\,}}}\limits _{s=-1} Q_{\alpha }(s)^{1/2} = \frac{1}{2}, \quad \mathop {{{\,\mathrm{Res}\,}}}\limits _{s=-\alpha } Q_{\alpha }(s)^{1/2} = \frac{1}{2}. \end{aligned}$$

The residues add up to zero, and since $Q_{\alpha }(s)^{1/2} = \frac{1}{s} + {\mathcal {O}}(s^{-2})$ as $s \rightarrow \infty$, we thus find from (4.23)

4.25 $$\begin{aligned} \int _{\Sigma _0} d\mu _0 = 1. \end{aligned}$$

Let $z(t) = \sqrt{\alpha } e^{i t}$, $-\theta _{\alpha }< t < \theta _{\alpha }$, be a parametrization of $\Sigma _0$. Then the mapping

4.26 $$\begin{aligned} t \mapsto \int _{z_-}^{z(t)} d\mu _0 = \frac{1}{\pi i} \int _{z_-}^{z(t)} Q_{\alpha ,-}(s)^{1/2} ds \end{aligned}$$

has as its derivative

$$\begin{aligned} \frac{1}{\pi i} Q_{\alpha ,-}(z(t))^{1/2} \cdot z'(t) \end{aligned}$$

which is real and non-zero for $t \in (-\theta _{\alpha }, \theta _{\alpha })$ since $Q_{\alpha }(z) (z')^2 < 0$ as $\Sigma _0$ is a trajectory of the quadratic differential by Lemma 4.5 (a).

Note also that the right-hand side of (4.26) vanishes for $t = - \theta _{\alpha }$ and equals 1 for $t= \theta _{\alpha }$ by (4.25). Therefore (4.26) is monotonically increasing from 0 to 1 as *t* goes from $-\theta _{\alpha }$ to $\theta _{\alpha }$, and this is enough to conclude that $\mu _0$ is a probability measure on $\Sigma _0$.

It now also follows (compare (4.15) and (4.26), and use $Q_{\alpha ,+}^{1/2} = - Q_{\alpha ,-}^{1/2}$ on $\Sigma _{0}$) that $\phi _-$ is purely imaginary along $\Sigma _0$ and we have

4.27 $$\begin{aligned} \phi _+(z) = - \phi _-(z), \qquad \text {for } z \in \Sigma _0. \end{aligned}$$

Next we calculate $g'(z) = \int \limits _{\Sigma _0} \frac{d\mu _0(s)}{z-s}$. We write $g'$ as a contour integral

$$\begin{aligned} g'(z) = \frac{1}{2\pi i} \oint _C \frac{Q_{\alpha }(s)^{1/2}}{z-s} ds, \qquad z \in {\mathbb {C}} {\setminus } \Sigma _0, \end{aligned}$$

with the same closed contour *C* as in (4.23), but we now also assume that *z* is in the exterior of *C*. We deform the contour to infinity where we now pick up a residue contribution from the pole at $s=z$ as well, which is $Q_{\alpha }(z)^{1/2}$. We use (4.24) to calculate the other residue contributions. There is no contribution from infinity and the result is that

4.28 $$\begin{aligned} g'(z)&= \frac{1}{z} - \frac{1}{2(z+1)} - \frac{1}{2(z+\alpha )} + Q_{\alpha }(z)^{1/2} \nonumber \\&= \frac{V_{\alpha }'(z)}{2} + \phi '(z), \qquad z \in {\mathbb {C}} {\setminus } \Sigma _0. \end{aligned}$$

Integrating (4.28) from $z_{+}$ to *z* along a path that does not intersect $(-\infty , 0] \cup \{\sqrt{\alpha } e^{it} \mid -\pi \le t \le \theta _{\alpha }\})$, we find

$$\begin{aligned} g(z) - g(z_{+}) = \frac{V_\alpha (z) - V_\alpha (z_{+})}{2} + \phi (z) - \phi (z_{+}), \end{aligned}$$

which proves (4.17) for $\alpha \in [\frac{1}{9},1]$ by the definition (4.14) of $\ell$ and the fact that $\phi (z_{+})=0$.

From (4.17) and (4.27) we obtain for $z \in \Sigma _0$,

$$\begin{aligned} g_+(z) + g_-(z) - V_{\alpha }(z) = \phi _+(z) + \phi _-(z) - \ell = -\ell , \end{aligned}$$

which proves (4.18). Also by (4.17) and (4.27)

$$\begin{aligned} g_+(z) - g_-(z) = \phi _+(z) - \phi _-(z) = 2 \phi _+(z) \end{aligned}$$

which is (4.19).

We have also shown that $\phi _-(z) \in i {\mathbb {R}}$ for $z \in \Sigma _0$, and similarly $\phi (z) \in i {\mathbb {R}}$ on the other critical trajectories that emanate from $z_+$ and $z_-$, see Fig. 8. Moreover, $\mathop {\mathrm {Im}}\phi$ is constant on orthogonal trajectories. We also saw that $\mathop {\mathrm {Im}}\phi _-(z)$ increases as *z* moves away from $z_-$ to $z_+$ along $\Sigma _0$. Then by the Cauchy-Riemann equations, we have $\mathop {\mathrm {Re}}\phi > 0$ in the domain on the minus side of $\Sigma _0$ and by continuity it holds in the outer domain bounded by the critical trajectories. Then $\mathop {\mathrm {Re}}\phi < 0$ if we cross the critical trajectory going around $-1$, and in particular $\mathop {\mathrm {Re}}\phi (z) < 0$ for *z* on the critical orthogonal trajectory from $z_+$ to $-\sqrt{\alpha }$. In view of (4.17), this gives

4.29 $$\begin{aligned} \mathop {\mathrm {Re}}\left[ 2g(z) - V_{\alpha }(z) + \ell \right] < 0, \end{aligned}$$

for *z* on this orthogonal trajectory, which is part of $\gamma _0 {\setminus } \Sigma _0$. This proves the inequality in (4.4). By symmetry the inequality also holds for *z* on the critical orthogonal trajectory from $z_-$ to $-\sqrt{\alpha }$. This completes the proof for the case $\alpha \ge \frac{1}{9}$.

The proof for $0< \alpha < \frac{1}{9}$ is simpler. In this case (4.9) is a rational function with partial fraction decomposition

$$\begin{aligned} Q_{\alpha }(s)^{1/2} = \frac{1}{s} + \frac{1}{2(s+1)} - \frac{1}{2(s+\alpha )}. \end{aligned}$$

The total integral of $\mu _0$ defined by (4.13) is

$$\begin{aligned} \int _{\Sigma _0} d\mu _0 = \frac{1}{\pi i} \oint _{\gamma _0} \left( \frac{1}{s} + \frac{1}{2(s+1)} - \frac{1}{2(s+\alpha )} \right) ds = 1 \end{aligned}$$

by a simple residue calculation with contributions only from the poles at $s=0$ and $s=-\alpha$. The total mass is 1 and as before it follows that $\mu _0$ is a probability measure.

We compute $g'(z)$ with another residue calculation

$$\begin{aligned} g'(z)&= \frac{1}{\pi i} \oint _{\gamma _0} \frac{1}{z-s} \left( \frac{1}{s} + \frac{1}{2(s+1)} - \frac{1}{2(s+\alpha )} \right) ds \\&= {\left\{ \begin{array}{ll} \frac{2}{z} - \frac{1}{z+\alpha }, &{} \text { if } |z| > \sqrt{\alpha }, \\ -\frac{1}{z+1}, &{} \text { if } |z| < \sqrt{\alpha }. \end{array}\right. } \end{aligned}$$

Recalling the definition (4.16) of $\phi (z)$ and the expression (4.8) for $V_\alpha '(z)$, we conclude

4.30 $$\begin{aligned} \phi '(z) = g'(z) - \frac{V_\alpha '(z)}{2}. \end{aligned}$$

Integrating (4.30) from $\sqrt{\alpha }$ to *z* along a path that does not intersect $(-\infty ,0] \cup \Sigma _0$, we find

4.31 $$\begin{aligned} \phi (z) = -\frac{\pi i}{2} + g(z) - \frac{V_\alpha (z)}{2} - g_-(\sqrt{\alpha }) + \frac{V_\alpha (\sqrt{\alpha })}{2}, \end{aligned}$$

if $|z| > \sqrt{\alpha }$. For $|z| < \sqrt{\alpha }$ we similarly find

$$\begin{aligned} \phi (z) = \frac{\pi i}{2} + g(z) - \frac{V_{\alpha }(z)}{2} - g_+(\sqrt{\alpha }) + \frac{V_{\alpha }(\sqrt{\alpha })}{2}. \end{aligned}$$

Then (4.31) also holds for $|z| < \sqrt{\alpha }$, since $g_+(\sqrt{\alpha }) = g_-(\sqrt{\alpha }) + \pi i$, as can be verified from the definition of the branch of $\log (z-s)$ that was used in the definition of *g*. Thus (4.17) holds for $0< \alpha < \frac{1}{9}$ in the low temperature regime because of the definition of the constant $\ell$. The identities (4.18) and (4.19) follow from (4.17) in the same way as in the case $\frac{1}{9} < \alpha \le 1$.

### Calculations leading to $Q_{\alpha }$

The reader may wonder how to obtain the expressions (2.5) and (2.7). One clue is that we need the residues (4.24). This translates into the three conditions (which are consistent with (4.6))

4.32 $$\begin{aligned} \begin{aligned} \lim _{z \rightarrow 0} z^2 Q_{\alpha }(z)&= 1, \\ \lim _{z \rightarrow -1} (z+1)^2 Q_{\alpha }(z)&= \frac{1}{4}, \\ \lim _{z \rightarrow -\alpha } (z+\alpha )^2 Q_{\alpha }(z)&= \frac{1}{4}. \end{aligned} \end{aligned}$$

It is also clear from (4.6) and (4.8) that

$$\begin{aligned} \lim _{z \rightarrow \infty } z^2 Q_{\alpha }(z) = 1. \end{aligned}$$

Then

$$\begin{aligned} Q_{\alpha }(z) = \frac{z^4 + A z^3 + B z^2 + C z + D}{z^2(z+1)^2(z+\alpha )^2} \end{aligned}$$

and the limits (4.32) give us three equations for the coefficients, namely

$$\begin{aligned} D = \alpha ^2, \quad C = \alpha A, \quad B = (\alpha +1)A - \frac{3}{4} \alpha ^2 - \frac{1}{2} \alpha - \frac{3}{4}. \end{aligned}$$

which leaves us with one parameter *A* only.

To proceed, we make the one-cut assumption which says that $Q_{\alpha }$ should have at least one multiple zero. It means that the discriminant of the numerator polynomial should be zero. The discriminant factors as

$$\begin{aligned} \alpha ^2(1-\alpha )^2 (A-\alpha -3)^2 (A-3\alpha -1)^2 \left( A^2- \frac{3}{2} (1+ \alpha ) A + \frac{9}{16}(1-\alpha )^2 \right) \end{aligned}$$

which leaves us with four possible choices for *A*, namely $A_1 = 3 +\alpha$, $A_2 = 3\alpha +1$, $A_3 = \frac{3}{4}(1-\sqrt{\alpha })^2$, and $A_4 = \frac{3}{4}(1+\sqrt{\alpha })^2$.

For $\alpha =1$ we should recover (2.9) which means that we have to take $A=A_4$ for $\alpha =1$, and then by continuity also for $\alpha$ between 1 and a critical value of $\alpha$. This leads to the formulas (2.5) and (2.4). The critical value is when $z_+(\alpha ) = z_-(\alpha )$, and this happens for $\alpha = 1/9$.

For $\alpha = \frac{1}{9}$, the two values $A_2$ and $A_4$ coincide, and for $\alpha < \frac{1}{9}$ we find that $A_2$ takes over. This leads to the formulas (2.7) and (2.6) with two double zeros of $Q_{\alpha }$.

## Orthogonal Polynomials and Riemann–Hilbert Problem

We will now prove the existence of the orthogonal polynomials and pose a RH problem for the reproducing kernel $R_N(w,z)$ that appears in the double contour integral in the kernel (1.7).

### Existence of the orthogonal polynomials

#### Proposition 5.1

Let $0 < \alpha \le 1$ and $N \in {\mathbb {N}}$. Then for every $n=0,1, \ldots , 2N$ there is a unique monic polynomial $p_n$ of degree *n* such that

5.1 $$\begin{aligned} \frac{1}{2\pi i} \oint _{\gamma } p_n(z) z^j \frac{(z+1)^N (z+\alpha )^N}{z^{2N}} dz = 0, \qquad j=0,1, \ldots , n-1. \end{aligned}$$

In addition, if $n \le 2N-1$, then

5.2 $$\begin{aligned} \kappa _n = \frac{1}{2\pi i} \oint _{\gamma } \left( p_n(z)\right) ^2 \frac{(z+1)^N (z+\alpha )^N}{z^{2N}} dz \ne 0. \end{aligned}$$

#### Proof

The orthogonality condition (5.1) is associated with the non-Hermitian bilinear form

$$\begin{aligned} \langle f, g \rangle = \frac{1}{2\pi i} \oint _{\gamma } f(z) g(z) \frac{(z+1)^N (z+\alpha )^N}{z^{2N}} dz \end{aligned}$$

defined for polynomials *f* and *g*. The polynomial $p_n$ exists and is unique if and only if the $n \times n$ matrix of moments

5.3 $$\begin{aligned} M_n = \begin{bmatrix} \langle z^j, z^k \rangle \end{bmatrix}_{j,k=0}^{n-1} \end{aligned}$$

is invertible. We use the Lindström–Gessel–Viennot (LGV) lemma to prove that this is the case for $n \le 2N$.

Consider the directed graph on ${\mathbb {Z}}^2$ with an edge from (*i*, *j*) to $(i',j')$ if and only if $i' = i + 1$ and $j' - j \in \{0,1\}$. The weights on the edges are

$$\begin{aligned} w((i,j), (i+1, j))&= {\left\{ \begin{array}{ll} \alpha &{} \text { if }i\text { is even}, \\ 1 &{} \text { if }i\text { is odd}, \end{array}\right. } \\ w((i,j), (i+1, j+1))&= 1. \end{aligned}$$

For two vertices $A, B \in \mathbb {Z}^2$ we define

$$\begin{aligned} w(A,B) = \sum _{P : A \rightarrow B } \prod _{e \in P} w(e), \end{aligned}$$

where the sum is over all directed paths *P* on the graph from vertex *A* to vertex *B*. If there are no such paths then $w(A,B) = 0$.

We assume $0 \le n \le 2N$ and we take vertices $A_j = (0, j)$ and $B_j = (2N, 2N-n+j)$ for $j=0, 1, \ldots , n-1$. The LGV lemma [41] states that $\det \left[ w(A_j, B_k) \right] _{j,k=0}^{n-1}$ is equal to the weighted sum of all non-intersecting path systems from $A_0, \ldots A_{n-1}$ to $B_0, \ldots , B_{n-1}$. It is easy to verify that there exist such non-intersecting path systems (due to the fact that $0 \le n \le 2N$). Each non-intersecting path system has a positive weight since $\alpha > 0$. Therefore $\det \left[ w(A_j, B_k) \right] _{j,k=0}^{n-1} > 0$, which, in particular, implies that

5.4 $$\begin{aligned} W_n = \left[ w(A_j, B_k) \right] _{j,k=0}^{n-1} \end{aligned}$$

is an invertible matrix.

To calculate $w(A_j,B_k)$ we observe that any path from $A_j$ to $B_k$ is of length 2*N* with $n-k+j$ horizontal edges. The weight of such a path is $\alpha ^l$ where *l* is the number of horizontal edges at an even level. We pick *l* out of the possible *N* even levels, and $n-k+j-l$ out of the possible *N* odd levels, and we see that there are $$ \left( {\begin{array}{c}N\\ l\end{array}}\right) \left( {\begin{array}{c}N\\ n-k+j-l\end{array}}\right) $$ paths from $A_j$ to $B_k$ with weight $\alpha ^l$. Summing over *l* yields

$$\begin{aligned} w(A_j,B_k) = \sum _{l=0}^{N} \left( {\begin{array}{c}N\\ l\end{array}}\right) \left( {\begin{array}{c}N\\ n-k+j-l\end{array}}\right) \alpha ^l. \end{aligned}$$

This sum over products of binomial coefficients is easily seen to be equal to the coefficient of $z^{2N-n+k-j}$ in the product $(z+1)^N (z+\alpha )^N$. Therefore, by Cauchy’s theorem

$$\begin{aligned} w(A_j,B_k)&= \frac{1}{2\pi i} \oint _{\gamma } \frac{(z+1)^N (z+\alpha )^N}{z^{2N-n+k-j+1}} dz \\&= \langle z^j, z^{n-k-1} \rangle . \end{aligned}$$

Comparing (5.3) and (5.4) we then see that $M_n$ is obtained from $W_n$ by reversing the order of the columns. Since $W_n$ is invertible, also $M_n$ is invertible, and it follows that $p_n$ uniquely exists.

To prove (5.2) let us assume that $\kappa _n = 0$. Then by orthogonality we have $\langle p_n, z^j \rangle = 0$ not only for $j=0,1, \ldots , n-1$ but also for $j=n$. It follows again by orthogonality of $p_{n+1}$ in case $n \le 2N-1$, that $\langle p_{n+1} + p_n, z^j \rangle = 0$ for every $j=0, 1, \ldots , n$. However, we established that $p_{n+1}$ is the only monic polynomial of degree $n+1$ with these properties (if $n\le 2N-1$). This contradiction shows that $\kappa _n \ne 0$. $\square$

### Riemann–Hilbert problem

It is well-known that the orthogonal polynomials and the associated Christoffel–Darboux kernel can be characterized by a RH problem.

#### Riemann–Hilbert Problem 5.2

Let $\gamma _0$ be the circle of radius $\sqrt{\alpha }$ around 0 with positive direction. Find a function $Y : \mathbb {C}{\setminus } \gamma _0 \rightarrow \mathbb {C}^{2\times 2}$ with the following properties:

- $$Y : \mathbb {C}{\setminus } \gamma _0 \rightarrow \mathbb {C}^{2\times 2}$$ is analytic.
- The limits of *Y*(*z*) as *z* approaches $\gamma _0$ from inside and outside exist, are continuous on $\gamma _0$ and are denoted by $Y_+$ and $Y_-$, respectively. Furthermore they are related by
  5.5 $$\begin{aligned} Y_{+}(z) = Y_{-}(z) \begin{pmatrix} 1 &{} \frac{(z+1)^{N}(z+\alpha )^{N}}{z^{2N}} \\ 0 &{} 1 \end{pmatrix} \qquad \text { for } z \in \gamma _0. \end{aligned}$$
- $$Y(z) = \left( I + {\mathcal {O}}(z^{-1})\right) \begin{pmatrix} z^{N} &{} 0 \\ 0 &{} z^{-N} \end{pmatrix}$$ as $$z \rightarrow \infty $$.

The RH problem 5.2 is due to Fokas, Its, and Kitaev [40]. Its solution contains the orthogonal polynomials of degrees *N* and $N-1$ that uniquely exist by Proposition 5.1,

5.6 $$\begin{aligned} Y(z) = \begin{pmatrix} p_N(z) &{}\quad \displaystyle \frac{1}{2\pi i} \oint _{\gamma _0} p_N(s) \frac{(s+1)^N(s+\alpha )^N}{s^{2N}} \frac{ds}{s-z} \\ -\kappa _{N-1}^{-1} p_{N-1}(z) &{}\quad \displaystyle -\frac{\kappa _{N-1}^{-1}}{2\pi i} \oint _{\gamma _0} p_{N-1}(s) \frac{(s+1)^N(s+\alpha )^N}{s^{2N}} \frac{ds}{s-z} \end{pmatrix}, \end{aligned}$$

for $$z \in {\mathbb {C}} {\setminus } \gamma _0$$.

#### Proposition 5.3

- The kernel $R_N$ is given in terms of the solution *Y* of the RH problem 5.2 by
  5.7 $$\begin{aligned} R_N(w,z) = \frac{1}{z-w} \begin{pmatrix} 0&1 \end{pmatrix} Y^{-1}(w) Y(z) \begin{pmatrix} 1 \\ 0 \end{pmatrix}. \end{aligned}$$
- Also for $$w, z \in {\mathbb {C}} {\setminus } \gamma _0$$,
  5.8 $$\begin{aligned} {\mathcal {R}}_N(w,z)&:= \begin{pmatrix} 1&0 \end{pmatrix} Y^{-1}(w) Y(z) \begin{pmatrix} 1 \\ 0 \end{pmatrix} \nonumber \\&= \frac{1}{2\pi i} \oint _{\gamma _0} R_N(s,z) \frac{(s+1)^N(s+\alpha )^N}{s^{2N}} \frac{s-z}{s-w} ds. \end{aligned}$$

#### Proof

The formula (5.7) is a reformulation of the Christoffel–Darboux formula (1.8), as can be readily checked from (5.6) together with the fact that $\det Y \equiv 1$. The formula (5.8) is obtained from (5.6) in a similar way. $\square$

### First transformation of the RH problem

The steepest descent analysis of the RH problem 5.2 for *Y* is fairly standard by now. It is modelled after the method developed by Deift et al. [28] for orthogonal polynomials on the real line. The extension to the complex plane is standard, once one has identified the correct contour $\gamma _0$ with the equilibrium measure $\mu _0$. In the high temperature regime we basically follow [28] including the construction of Airy parametrices for the local analysis at branch points $z_{\pm }$. The RH analysis in the low temperature regime is even simpler since we can separate contours and no local analysis is needed. The critical case $\alpha = 1/9$ is more difficult, but can be handled with the construction of a local parametrix built out of Lax pair solutions associated with the Hastings-McLeod solution of Painlevé II. This is similar to the construction in [23] for orthogonal polynomials on the real line in cases where the equilibrium density vanishes quadratically at an interior point of its support. We will not give any details for this case.

In terms of the function $V_\alpha$ defined in (4.1), the jump relation (5.5) for *Y* can be expressed as

$$\begin{aligned} Y_{+}(z) = Y_{-}(z) \begin{pmatrix} 1 &{}\quad e^{-NV_\alpha (z)} \\ 0 &{}\quad 1 \end{pmatrix} \qquad \text { for } z \in \gamma _0. \end{aligned}$$

The first transformation $Y \mapsto T$ uses the *g*-function from Definition 4.2 to normalize the RH problem at infinity. We define

5.9 $$\begin{aligned} T(z) = e^{\frac{N\ell }{2}\sigma _{3}}Y(z)e^{-Ng(z)\sigma _{3}}e^{-\frac{N\ell }{2}\sigma _{3}}, \qquad \sigma _3 {:}{=}\begin{pmatrix} 1 &{}\quad 0 \\ 0 &{}\quad -1 \end{pmatrix}. \end{aligned}$$

The jumps in the RH problem for *T* are conveniently expressed in terms of the function $\phi$ defined in (4.15) and (4.16). From the identities (4.17), (4.18), and (4.19) and the definition (5.9), we find the following RH problem.

#### Riemann–Hilbert Problem 5.4

*T* satisfies

- $$T : \mathbb {C}{\setminus } \gamma _0 \rightarrow \mathbb {C}^{2\times 2}$$ is analytic.
- *T* has boundary values on $\gamma _0$ that satisfy
  5.10 $$\begin{aligned} T_{+}(z)&= T_{-}(z) \begin{pmatrix} e^{-2N\phi _{+}(z)} &{} 1 \\ 0 &{} e^{-2N\phi _{-}(z)} \end{pmatrix},&\text { for } z \in \Sigma _0 \subset \gamma _0, \end{aligned}$$
  5.11 $$\begin{aligned} T_{+}(z)&= T_{-}(z) \begin{pmatrix} 1 &{} e^{2N\phi (z)} \\ 0 &{} 1 \end{pmatrix},&\text { for } z \in \gamma _0 {\setminus } \Sigma _0. \end{aligned}$$
- $$T(z) = I + {\mathcal {O}}(z^{-1})$$ as $$z \rightarrow \infty $$.

Note that *T* depends on *N*, even though this is not indicated in the notation. What is important for us, is that *T* and $T^{-1}$ remain bounded as $N \rightarrow \infty$, provided we stay away from the branch points $z_{\pm }(\alpha )$ (only in the high temperature regime). We summarize what we need from the RH analysis in the following proposition.

#### Proposition 5.5

- If $0 < \alpha \le \frac{1}{9}$, then both *T*(*z*) and $T(z)^{-1}$ are uniformly bounded for $z \in \mathbb {C}{\setminus } \gamma _{0}$ as $N \rightarrow \infty$.
- If $\frac{1}{9} < \alpha \le 1$, then $T(z) = {\mathcal {O}}(N^{1/6})$ and $T^{-1}(z) = {\mathcal {O}}(N^{1/6})$ as $N \rightarrow \infty$, uniformly for $z \in {\mathbb {C}} {\setminus } \gamma _0$. In addition, for every $\delta > 0$, we have that *T*(*z*) and $T^{-1}(z)$ are bounded as $N \rightarrow \infty$ uniformly for *z* in the domain
  5.12 $$\begin{aligned} \{ z \in {\mathbb {C}} \mid |z-z_+(\alpha )| \ge \delta , |z-z_-(\alpha )| \ge \delta \}. \end{aligned}$$

The proposition is a result of the steepest descent analysis that we will perform next for the two regimes separately.

Because of (5.9) and the formula (5.8) for ${\mathcal {R}}_N$, we have

5.13 $$\begin{aligned} {\mathcal {R}}_N(w,z) = \begin{pmatrix} 1&0 \end{pmatrix} T^{-1}(w) T(z) \begin{pmatrix} 1 \\ 0 \end{pmatrix} e^{N(g(z)-g(w))} \end{aligned}$$

and before turning to the proof of Proposition 5.5 we note the following consequence.

#### Corollary 5.6

- If $0 < \alpha \le \frac{1}{9}$ then ${\mathcal {R}}_N(w,z) e^{N(g(w) - g(z))}$ remains bounded as $N \rightarrow \infty$, uniformly for $w \in {\mathbb {C}} {\setminus } \gamma _0$ and $z \in {\mathbb {C}}{\setminus } \gamma _0$.
- If $\frac{1}{9} < \alpha \le 1$ then ${\mathcal {R}}_N(w,z) e^{N(g(w) - g(z))}$ remains bounded as $N \rightarrow \infty$, uniformly for $w \in {\mathbb {C}} {\setminus } \gamma _0$ and $z \in {\mathbb {C}}$, both in the domain (5.12) for some $\delta > 0$.
- If $\frac{1}{9} < \alpha \le 1$, then the analytic continuation of $w \mapsto {\mathcal {R}}_N(w,z) e^{N(g(w)-g(z))}$ from the disk $|w| < \sqrt{\alpha }$ across $\gamma _0 {\setminus } \Sigma _0$ into the domain bounded by $\Sigma _{-1}$ and $\gamma _0 {\setminus } \Sigma _0$ remains bounded as $N \rightarrow \infty$, again uniformly for *w* and *z* in the domain (5.12) for some $\delta > 0$.

#### Proof

Parts (a) and (b) are immediate from (5.13) and Proposition 5.5.

Because of (5.13) and the jump condition (5.11) for *T* along $\gamma _0 {\setminus } \Sigma _0$, the analytic continuation from part (c) is given by

$$\begin{aligned} \begin{pmatrix} 1&- e^{2N \phi (w)} \end{pmatrix} T^{-1}(w) T(z) \begin{pmatrix} 1 \\ 0 \end{pmatrix} \end{aligned}$$

Since $\mathop {\mathrm {Re}}\phi (w) < 0$ for *w* in the region under consideration in part (c), see for example Fig. 9, part (c) follows from Proposition 5.5 as well. $\square$

### Proof of Proposition 5.5 (a)

#### Proof

Suppose $0< \alpha < \frac{1}{9}$. Then we can find contours $\gamma _{+}$ and $\gamma _{-}$ lying in the interior and exterior of $\gamma _0 = \Sigma _0$, respectively, such that

**Fig. 11:**
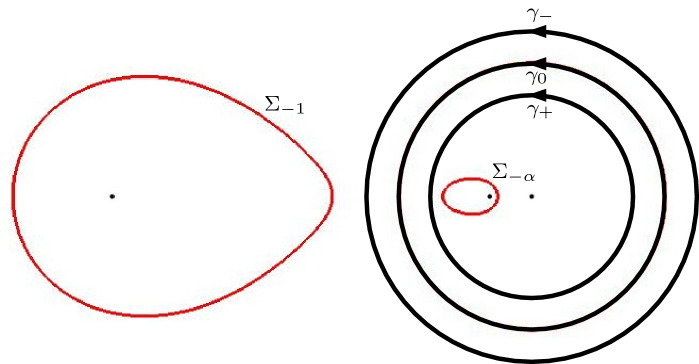
The jump contour $\gamma _0 \cup \gamma _+ \cup \gamma _-$ for the RH problem 5.7 for *S* (black), the curves $\Sigma _{-1}$ and $\Sigma _{-\alpha }$ (red), and the points $-\,1, -\,\alpha , 0$ (black dots) in the low temperature regime

5.14 $$\begin{aligned} \mathop {\mathrm {Re}}\phi (z)> \epsilon > 0 \qquad \text {for all } z \in \gamma _{+}\cup \gamma _{-} \end{aligned}$$

for some fixed $\epsilon > 0$, see Fig. 11.

We define

5.15 $$\begin{aligned} S(z) = T(z) \times {\left\{ \begin{array}{ll} \begin{pmatrix} 1 &{} 0 \\ -e^{-2N\phi (z)} &{} 1 \end{pmatrix}, &{} \text {for }z\text { between }\gamma _{0}\text { and }\gamma _+, \\ \begin{pmatrix} 1 &{} 0 \\ e^{-2N\phi (z)} &{} 1 \end{pmatrix}, &{} \text {for }z\text { between }\gamma _{0}\text { and }\gamma _-, \\ I, &{} \text {elsewhere}. \end{array}\right. } \end{aligned}$$

Then *S* satisfies the following RH problem. $\square$

#### Riemann–Hilbert Problem 5.7

 

- $$S : \mathbb {C}{\setminus } (\gamma _0 \cup \gamma _{+} \cup \gamma _{-}) \rightarrow \mathbb {C}^{2\times 2}$$ is analytic.
- *S* has boundary values on $\gamma _0$, $\gamma _+$ and $\gamma _-$ that satisfy
  $$\begin{aligned} S_{+}(z)&= S_{-}(z) \begin{pmatrix} 1 &{} 0 \\ e^{-2N\phi (z)} &{} 1 \end{pmatrix},&\text { for } z \in \gamma _{+} \cup \gamma _{-}, \\ S_{+}(z)&= S_{-}(z) \begin{pmatrix} 0 &{} 1 \\ -1 &{} 0 \end{pmatrix},&\text { for } z \in \gamma _{0}. \end{aligned}$$
- $$S(z) = I + {\mathcal {O}}(z^{-1})$$ as $$z \rightarrow \infty $$.

We remove the constant jump on $\gamma _0$ by defining

5.16 $$\begin{aligned} R(z) = S(z) \times {\left\{ \begin{array}{ll} \begin{pmatrix} 0 &{} -1 \\ 1 &{} 0 \end{pmatrix}, &{} \text { for }z\text { inside }\gamma _0, \\ I, &{} \text { for }z\text { outside }\gamma _0. \end{array}\right. } \end{aligned}$$

Of course *R* should not be confused with the reproducing kernel $R_N$, as these are totally different objects. The matrix valued function *R* satisfies the following RH problem.

#### Riemann–Hilbert Problem 5.8

- $$R: \mathbb {C}{\setminus } (\gamma _{+} \cup \gamma _{-}) \rightarrow \mathbb {C}^{2\times 2}$$ is analytic.
- *R* has boundary values on $\gamma _+$ and $\gamma _-$ that satisfy
  $$\begin{aligned} R_{+}(z)&= R_{-}(z) \begin{pmatrix} 1 &{} -e^{-2N\phi (z)} \\ 0 &{} 1 \end{pmatrix},&\text { for } z \in \gamma _{+}, \\ R_{+}(z)&= R_{-}(z) \begin{pmatrix} 1 &{} 0 \\ e^{-2N\phi (z)} &{} 1 \end{pmatrix},&\text { for } z \in \gamma _{-}. \end{aligned}$$
- $$R(z) = I + {\mathcal {O}}(z^{-1})$$ as $$z \rightarrow \infty $$.

Since $\mathop {\mathrm {Re}}\phi> \epsilon > 0$ for $z \in \gamma _+ \cup \gamma _-$ the jumps in the RH problem for *R* are exponentially close to the identity matrix as $N \rightarrow \infty$. By standard estimates on small norm RH problems [27], we find $R(z) = I + {\mathcal {O}}(e^{-\epsilon N})$ as $N \rightarrow \infty$, and in particular *R* and $R^{-1}$ are uniformly bounded as $N \rightarrow \infty$, uniformly on ${\mathbb {C}}$. Tracing back the transformations (5.16) and (5.15) it then also follows that *T* and $T^{-1}$ are uniformly bounded as $N \rightarrow \infty$, uniformly on ${\mathbb {C}}$, since $\mathop {\mathrm {Re}}\phi \ge 0$ in the annular region bounded by $\gamma _+$ and $\gamma _-$. This proves Proposition 5.5 for $\alpha < \frac{1}{9}$.

In case $\alpha = \frac{1}{9}$, we are not able to choose $\gamma _+$ and $\gamma _-$ such that (5.14) holds on the full contours. Instead we let $\gamma _+$ and $\gamma _-$ go to $\gamma _0$ at the critical point $-\sqrt{\alpha } = - \frac{1}{3}$, and we can do it in such a way $\mathop {\mathrm {Re}}\phi > 0$ on $(\gamma _+ \cup \gamma _-) {\setminus } \{-\frac{1}{3}\}$. Then we can proceed as in the case $\alpha < \frac{1}{9}$ described above, except that we have to build a local parametrix at $-\frac{1}{3}$. This is done with the help of a special parametrix [23] that we will not describe here. We only need to know that it is uniformly bounded as $N \rightarrow \infty$ and then Proposition 5.5 follows as before. $\square$

**Fig. 12:**
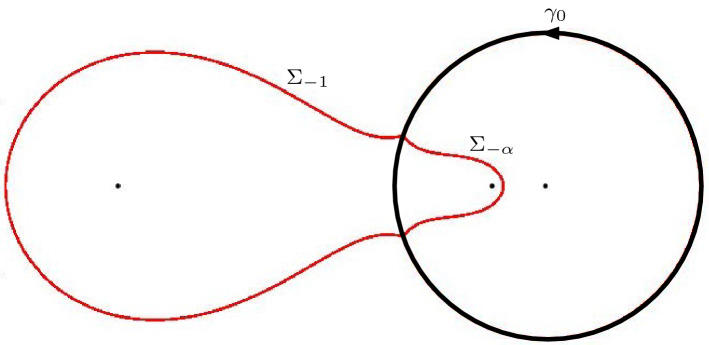
The jump contour $\gamma _0$ for the RH problem 5.2 for *Y* (black), the curves $\Sigma _{-1}$ and $\Sigma _{-\alpha }$ (red), and the points $-\,1, -\,\alpha , 0$ (black dots) in the high temperature regime

### Proof of Proposition 5.5 (b)

#### Proof

Suppose $\frac{1}{9} < \alpha \le 1$ and let *Y*(*z*) denote the solution of the RH problem 5.2 with jump contour $\gamma _0$. See Fig. 12 for $\gamma _0$ together with the contours $\Sigma _{-1}$ and $\Sigma _{-\alpha }$ that enclose the bounded domain where $\mathop {\mathrm {Re}}\phi < 0$ in the high temperature regime.

**Fig. 13:**
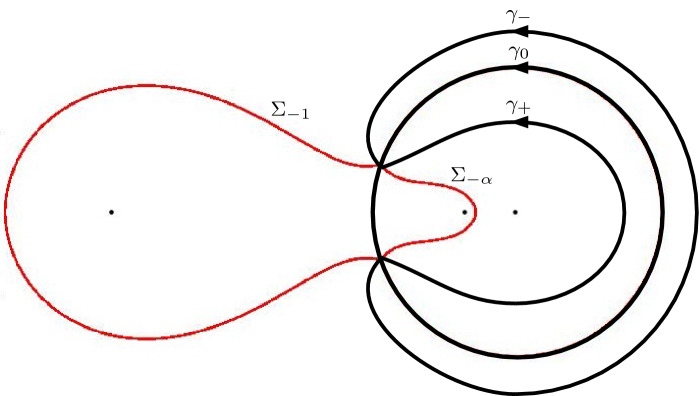
The jump contour $\gamma _0 \cup \gamma _+ \cup \gamma _-$ for the RH problem for *S* (black) and the curves $\Sigma _{-1}$ and $\Sigma _{-\alpha }$ (red) in the high temperature regime

The first transformation $Y \rightarrow T$ is given by (5.9) and *T* satisfies the RH problem 5.4. In the second transformation, we open up lenses $\gamma _{+}$ and $\gamma _{-}$ around $\Sigma _0 \subset \gamma _0$ as in Fig. 13 such that $\mathop {\mathrm {Re}}\phi > 0$ on $(\gamma _+ \cup \gamma _-) {\setminus } \{z_+(\alpha ), z_-(\alpha )\}$ and define *S* as (it is similar to (5.15))

5.17 $$\begin{aligned} S(z) = T(z) \times {\left\{ \begin{array}{ll} \begin{pmatrix} 1 &{} 0 \\ -e^{-2N\phi (z)} &{} 1 \end{pmatrix}, &{} \text {for }z\text { between }\Sigma _{0}\text { and }\gamma _+, \\ \begin{pmatrix} 1 &{} 0 \\ e^{-2N\phi (z)} &{} 1 \end{pmatrix}, &{} \text {for }z\text { between }\Sigma _{0}\text { and }\gamma _-, \\ I, &{} \text {elsewhere}. \end{array}\right. } \end{aligned}$$

Then *S* satisfies. $$\square $$

#### Riemann–Hilbert Problem 5.9

- $$S : \mathbb {C}{\setminus } (\gamma _0 \cup \gamma _{+} \cup \gamma _{-}) \rightarrow \mathbb {C}^{2\times 2}$$ is analytic.
- *S* has boundary values on $\gamma _0$, $\gamma _+$ and $\gamma _-$ that satisfy
  $$\begin{aligned} S_{+}(z)&= S_{-}(z) \begin{pmatrix} 1 &{} 0 \\ e^{-2N\phi (z)} &{} 1 \end{pmatrix},&\text { for } z \in \gamma _{+} \cup \gamma _{-}, \\ S_{+}(z)&= S_{-}(z) \begin{pmatrix} 0 &{} 1 \\ -1 &{} 0 \end{pmatrix},&\text { for } z \in \Sigma _{0}, \\ S_{+}(z)&= S_{-}(z) \begin{pmatrix} 1 &{} e^{2N \phi (z)} \\ 0 &{} 1 \end{pmatrix},&\text { for } z \in \gamma _{0} {\setminus } \Sigma _0. \end{aligned}$$
- $$S(z) = I + {\mathcal {O}}(z^{-1})$$ as $$z \rightarrow \infty $$.

The global parametrix $P^{(\infty )}$ is given by

5.18 $$\begin{aligned} P^{(\infty )}(z) = \begin{pmatrix} \frac{1}{2}(a(z)+a(z)^{-1}) &{} \frac{1}{2i}(a(z)-a(z)^{-1}) \\ -\frac{1}{2i}(a(z)-a(z)^{-1}) &{} \frac{1}{2}(a(z)+a(z)^{-1}) \end{pmatrix}, \end{aligned}$$

where $a(z) := \big (\frac{z-z_{+}}{z-z_{-}}\big )^{1/4}$ is defined with a branch cut along $\Sigma _0$ and in such a way that $a(z) \rightarrow 1$ as $z \rightarrow \infty$.

In small disks $\mathcal {D}_{z_{+}}$ and $\mathcal {D}_{z_{-}}$ around the endpoints of $\Sigma _0$ we construct local parametrices $P^{(z_+)}$ and $P^{(z_-)}$ with the aid of Airy functions. This construction is standard by now and we do not give details. The only thing that concerns us is that the local parametrices depend on *N* and they slightly grow with *N*, namely

5.19 $$\begin{aligned} P^{(z_{\pm })}(z) = {\mathcal {O}}(N^{\frac{1}{6}}), \quad P^{(z_{\pm })}(z)^{-1} = {\mathcal {O}}(N^{\frac{1}{6}}) \quad \text { as } N \rightarrow \infty , \end{aligned}$$

uniformly for $z \in {\mathcal {D}}_{z_{\pm }}$. The third and final transformation $S \mapsto R$ is defined by

5.20 $$\begin{aligned} R(z) = {\left\{ \begin{array}{ll} S(z) P^{(\infty )}(z)^{-1}, &{} \text { for } z \in \mathbb {C}{\setminus } (\mathcal {D}_{z_{+}} \cup \mathcal {D}_{z_{-}}), \\ S(z) P^{(z_{+})}(z)^{-1}, &{} \text { for } z \in \mathcal {D}_{z_{+}}, \\ S(z) P^{(z_{-})}(z)^{-1}, &{} \text { for } z \in \mathcal {D}_{z_{-}}. \end{array}\right. } \end{aligned}$$

Then *R* is defined and analytic in

$$\begin{aligned} {\mathbb {C}} {\setminus } \Big (\big ( (\gamma _0 \cup \gamma _{+} \cup \gamma _{-}){\setminus } (\mathcal {D}_{z_{+}} \cup \mathcal {D}_{z_{+}})\big ) \cup \partial \mathcal {D}_{z_+} \cup \partial {\mathcal {D}}_{z_-}\Big ) \end{aligned}$$

with jump matrices that are $I + {\mathcal {O}}(N^{-1})$ as $N \rightarrow \infty$. It follows that $R(z) = I + {\mathcal {O}}(N^{-1})$ uniformly for $z \in {\mathbb {C}}$, and in particular *R* and $R^{-1}$ remain bounded as $N \rightarrow \infty$. Observe that in the construction of the local parametrics, the disks $\mathcal {D}_{z_{\pm }}$ can be chosen arbitrarily small (but independent of *N*), and we choose them with radii smaller than $\delta$. Then following the transformations (5.17) and (5.20), and taking note of (5.19) we find that *T* and $T^{-1}$ are uniformly of order $N^{\frac{1}{6}}$ as $N \rightarrow \infty$. Outside the disks ${\mathcal {D}}_{z_\pm }$ the global parametrix (5.18) applies, which does not change with *N*, and then *T* and $T^{-1}$ remain uniformly bounded. Part (b) of Proposition 5.5 is now also proven. $\square$

## Phase Functions $\Phi _{\alpha }$ and $\Psi _{\alpha }$

### Definitions

In the last two sections we analyzed the RH problem with the *g*-function coming from the equilibrium measure as its main input. The outcome of this analysis is in Corollary 5.6 which states that ${\mathcal {R}}_N(w,z) e^{N(g(w)-g(z))}$ remains uniformly bounded in certains regions, and actually (very roughly)

6.1 $$\begin{aligned} {\mathcal {R}}_N(w,z) \sim e^{N(g(z)-g(w))} \end{aligned}$$

as $$N \rightarrow \infty $$.

We now turn to the asymptotic analysis of the double contour integrals coming from (1.7) and that give the probabilities for the three types of lozenges, see also Theorem 7.1 below.

After deforming contours and splitting up integrals, we are able to rewrite the integrals with an integrand containing

6.2 $$\begin{aligned} {\mathcal {R}}_N(w,z) \frac{F(z;x_{1},y_{1})}{F(w;x_{2},y_{2})} \end{aligned}$$

as the main *N*-dependent entry, where

6.3 $$\begin{aligned} F(z;x,y) = \frac{(z+1)^{\lfloor \frac{x}{2} \rfloor } (z+\alpha )^{\lfloor \frac{x+1}{2} \rfloor }}{z^y}, \end{aligned}$$

see Propositions 7.8 and 7.9. Recall that *x*, *y* will be varying with *N* as in (2.1). Then in view of (6.1), (6.3) we see that (6.2) behaves roughly like $e^{N (\Phi _{\alpha }(z) - \Phi _{\alpha }(w))}$ with a certain function $\Phi _{\alpha }$ that depends ons $(\xi ,\eta ) \in {\mathcal {H}}$, and that is defined next, along with a companion function $\Psi _{\alpha }$.

#### Definition 6.1

For $(\xi ,\eta ) \in {\mathcal {H}}$ we define

6.4 $$\begin{aligned} \Phi _{\alpha }(z)&= \Phi _{\alpha }(z;\xi ,\eta ) \nonumber \\&= g(z) + \frac{1+\xi }{2} \log \left( (z+1)(z+\alpha )\right) - (1+\eta ) \log z + \frac{\ell }{2} \nonumber \\&= \phi (z) + \frac{\xi }{2}\log \left( (z+1)(z+\alpha )\right) - \eta \log z, \end{aligned}$$

6.5 $$\begin{aligned} \Psi _{\alpha }(z)&= \Psi _{\alpha }(z;\xi ,\eta ) = -\Phi _{\alpha }(z;-\xi ,-\eta )\nonumber \\&= -\phi (z) + \frac{\xi }{2}\log \left( (z+1)(z+\alpha )\right) - \eta \log z. \end{aligned}$$

The equality leading to the third line in (6.4) follows from (4.17) and (4.1). Recall that $\phi ' = \pm Q_{\alpha }^{1/2}$ by Definition 4.3 and therefore by the definitions (6.4) and (6.5) we have that both $\Phi _{\alpha }'$ and $\Psi _{\alpha }'$ satisfy the algebraic equation (2.11) for $\Xi _{\alpha }$.

Thus $\Phi _{\alpha }'$ and $\Psi _{\alpha }'$ are two branches of the algebraic function $\Xi _{\alpha }$. Taking note of the different choice of branch cuts in the high temperature regime we can verify that

6.6 $$\begin{aligned} \Phi _{\alpha }'(z) = {\left\{ \begin{array}{ll} \Xi _{\alpha ,+}(z), &{} |z|> \sqrt{\alpha }, \\ \Xi _{\alpha ,-}(z), &{} |z|< \sqrt{\alpha }, \end{array}\right. }, \qquad \Psi _{\alpha }'(z) = {\left\{ \begin{array}{ll} \Xi _{\alpha ,-}(z), &{} |z| > \sqrt{\alpha }, \\ \Xi _{\alpha ,+}(z), &{} |z| < \sqrt{\alpha }, \end{array}\right. } \end{aligned}$$

in both regimes.

The two functions are defined and analytic in ${\mathbb {C}} {\setminus } ((-\infty ,0] \cup \Sigma _0)$ in case $0 < \alpha \le \frac{1}{9}$ and in ${\mathbb {C}} {\setminus } ((-\infty ,0] \cup \{ \sqrt{\alpha } e^{it} \mid -\pi \le t \le \theta _{\alpha }\}$ in case $\frac{1}{9} < \alpha \le 1$. The behavior at the singularities and at infinity can be seen from (4.22) and the definitions (6.4)–(6.5), namely for $(\xi ,\eta ) \in {\mathcal {H}}^o$,

6.7 $$\begin{aligned} \begin{aligned} \Phi _{\alpha }(z)&= -(1+\eta ) \log z + O(1) \text { as } z \rightarrow 0,&\lim _{z \rightarrow 0} \mathop {\mathrm {Re}}\Phi _{\alpha }(z)&= +\infty , \\ \Phi _{\alpha }(z)&= \frac{1}{2}(1+\xi ) \log (z+\alpha ) + O(1) \text { as } z \rightarrow -\alpha ,&\lim _{z \rightarrow -\alpha } \mathop {\mathrm {Re}}\Phi _{\alpha }(z)&= - \infty , \\ \Phi _{\alpha }(z)&= \frac{1}{2}(1+\xi ) \log (z+1) + O(1) \text { as } z \rightarrow -1,&\lim _{z \rightarrow - 1} \mathop {\mathrm {Re}}\Phi _{\alpha }(z)&= -\infty , \\ \Phi _{\alpha }(z)&= (1+\xi -\eta ) \log z + O(1) \text { as } z \rightarrow \infty ,&\lim _{z \rightarrow \infty } \mathop {\mathrm {Re}}\Phi _{\alpha }(z)&= +\infty \end{aligned} \end{aligned}$$

and similarly $\mathop {\mathrm {Re}}\Psi _{\alpha }(z) \rightarrow -\infty$ as $z \rightarrow 0$ or $z \rightarrow \infty$, and $\mathop {\mathrm {Re}}\Psi _{\alpha }(z) \rightarrow + \infty$ as $z \rightarrow -1$ or $z \rightarrow -\alpha$. For the limits it is important that $(\xi ,\eta ) \in {\mathcal {H}}^o$ so that $-1< \xi , \eta -\xi < 1$.

For each $(\xi , \eta ) \in \mathcal {L}_\alpha$, the saddle $s(\xi ,\eta ;\alpha )$ defined in Definition 2.4 is a zero of either $\Phi '_{\alpha }$ and $\Psi '_{\alpha }$.

#### Lemma 6.2

Let $(\xi ,\eta ) \in {\mathcal {L}}_{\alpha }$ and $s = s(\xi ,\eta ;\alpha )$. Then we have

- $\Phi _{\alpha }'(s) = 0$ and $|s| < \sqrt{\alpha }$ if and only if $\xi < 0$ and $\eta < \frac{\xi }{2}$,
- $\Phi _{\alpha }'(s)=0$ and $|s| > \sqrt{\alpha }$ if and only if $\xi < 0$ and $\eta > \frac{\xi }{2}$,
- $\Psi _{\alpha }'(s)=0$ and $|s| < \sqrt{\alpha }$ if and only if $\xi > 0$ and $\eta > \frac{\xi }{2}$,
- $\Psi _{\alpha }'(s)=0$ and $|s| >\sqrt{\alpha }$ if and only if $\xi > 0$ and $\eta < \frac{\xi }{2}$,
- $|s| = \sqrt{\alpha }$ if and only if $\xi = 0$ or $\eta = \frac{\xi }{2}$.

#### Proof

We use the explicit inverses for the map $(\xi ,\eta ) \mapsto s(\xi ,\eta ;\alpha )$ given in (3.4) and (3.7).

Let us consider the low temperature regime. From the formula (2.12) for $\Xi _{\alpha ,\pm }$ and (2.18) it follows that *s* is a zero of $\Xi _{\alpha ,\pm }$ if and only if $D_{\pm }<0$, and we note that the regions $D_{\pm }<0$ are contained in the regions $\eta > \frac{\xi }{2}$ and $\eta < \frac{\xi }{2}$, respectively. Using (3.3) and (3.4) we see that, in the low temperature regime, $\xi$ has the same sign as

6.8 $$\begin{aligned} {\mp } \mathop {\mathrm {Im}}\frac{(s-z_+)(s-z_-)}{(s+ \alpha )(s+1)}, \end{aligned}$$

with a ${\mp }$-sign if $s=s(\xi ,\eta ;\alpha )$ is a zero of $\Xi _{\alpha ,\pm }$. The imaginary part in (6.8) is positive if $|s|>\sqrt{\alpha }$, negative if $|s|< \sqrt{\alpha }$ and zero if $|s|= \sqrt{\alpha }$. Combining this with (6.6) the statements of the lemma follow in the low temperature regime.

For the high temperature regime, we use Proposition 2.7 and the proof is analogous to the proof in the low temperature regime, but now (6.8) is replaced by ${\mp } \mathop {\mathrm {Im}}s Q_\alpha (s)^{\frac{1}{2}}$, with the same choice of branch for the square root as in (3.7), i.e., $Q_\alpha (s)^{\frac{1}{2}}$ has a branch on ${\mathcal {C}}$. $\square$

### Critical level set of $\mathop {\mathrm {Re}}\Phi _{\alpha }$

In what follows we focus on the case (a) of Lemma 6.2, namely $(\xi ,\eta ) \in {\mathcal {L}}_{\alpha }$ with $\eta< \frac{\xi }{2} < 0$, and its extension $\eta = \frac{\xi }{2} < 0$, which is the lower left part of the liquid region. The corresponding saddle $s = s(\xi ,\eta ;\alpha )$ satisfies $\Phi '_{\alpha }(s)=0$ and $|s| < \sqrt{\alpha }$ if $\eta < \frac{\xi }{2}$. For $\eta = \frac{\xi }{2} < 0$ (which is only relevant in the high temperature regime) we have $|s| = \sqrt{\alpha }$ with $\theta _{\alpha }< \arg s < \pi$, and we still have $\Phi '_{\alpha }(s) = 0$.

We are interested in the level set of $\mathop {\mathrm {Re}}\Phi _{\alpha }$ that contains *s*,

6.9 $$\begin{aligned} {\mathcal {N}}_{\Phi } = \{ z \in {\mathbb {C}} \mid \mathop {\mathrm {Re}}\Phi _{\alpha }(z) = \mathop {\mathrm {Re}}\Phi _{\alpha }(s) \}. \end{aligned}$$

We emphasize that $\Phi _{\alpha }$ has a branch cut along $\Sigma _0$. However $\mathop {\mathrm {Re}}\Phi _{\alpha }$ is well-defined and continuous, also on $\Sigma _0$.

**Fig. 14:**
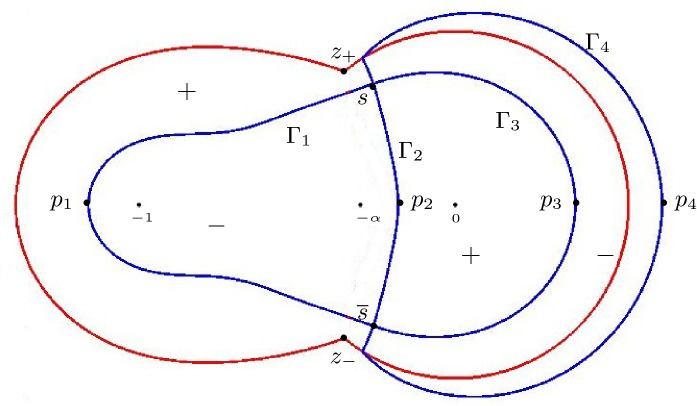
The level set $\mathcal {N}_{\Phi }$ (blue) in the high temperature regime (for $\alpha = 0.3$) in case $\Gamma _1$ intersects the real line at $p_1 < -1$. The $+$ and − signs indicate the sign of $\mathop {\mathrm {Re}}(\Phi _{\alpha } - \Phi _{\alpha }(s))$

**Fig. 15:**
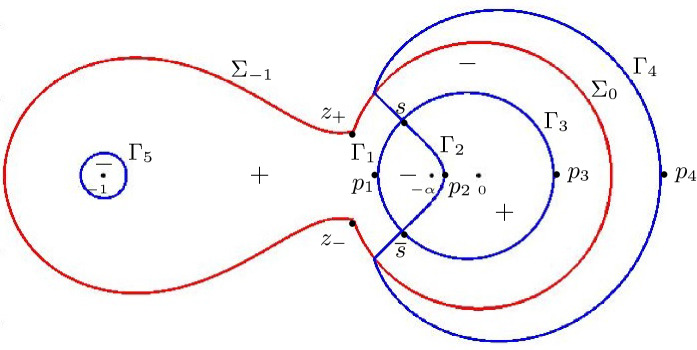
The level set $\mathcal {N}_{\Phi }$ (blue) and the contours $\Sigma _{-1}\cup \Sigma _0$ in the high temperature regime (here $\alpha = \frac{1}{8}$) in case $-1< p_1 < -\alpha$. The set $\mathcal {N}_{\Phi }$ divides the plane into five regions and the sign of $\mathop {\mathrm {Re}}(\Phi _{\alpha }-\Phi _{\alpha }(s))$ is indicated in each of these five regions by $+$ or −

**Fig. 16:**
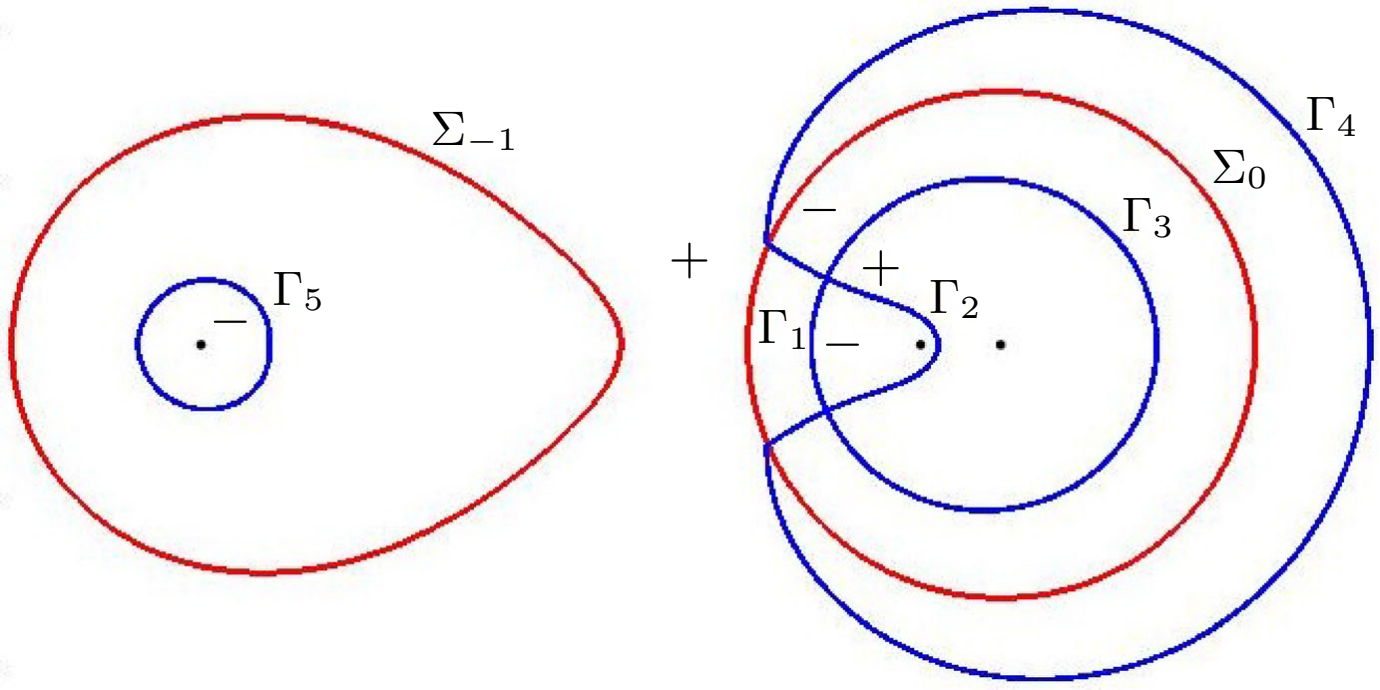
The level set $\mathcal {N}_{\Phi }$ (blue) and the contours $\Sigma _{-1}$ and $\Sigma _0$ in the low temperature regime (here $\alpha = \frac{1}{10}$). The set $\mathcal {N}_{\Phi _{\alpha }}$ divides the plane into five regions and the sign of $\mathop {\mathrm {Re}}(\Phi _{\alpha }-\Phi _{\alpha }(s))$ is indicated in each of these five regions by $+$ or −

Typical behaviors of ${\mathcal {N}}_{\Phi }$ are shown in Figs. 14, 15 and 16. The level set ${\mathcal {N}}_{\Phi }$ makes a cross locally at *s* since it is a simple saddle. Four curves emanate from *s* that are denoted by $\Gamma _1$, ..., $\Gamma _4$ in the figures.

It is important for us that three of these curves stay inside $\Sigma _0$ (in low temperature regime) or inside $\Sigma _0 \cup \Sigma _{-1}$ and connect *s* with $\overline{s}$. Only one of them (denoted by $\Gamma _4$ in the figures) meets with either $\Sigma _0$ or $\Sigma _0 \cup \Sigma _{-1}$.

To be able to prove this we need information on the behavior of the two functions $z \mapsto \log |z|$ and $z \mapsto \log \left| \frac{(z+1)(z+\alpha )}{z} \right|$ on $\Sigma _{-1} \cup \Sigma _{0}$. We start with a lemma.

#### Lemma 6.3

We have the following for $0 < \alpha \le 1$,

- $z^2 Q_{\alpha }(z) \in [0,\infty )$ if and only if $z \in \Sigma _0 \cup {\mathbb {R}} {\setminus } \{-1,-\alpha \}$.
- If $\alpha \le \frac{1}{9}$ then $\mathop {\mathrm {Im}}\left[ \frac{z^2-\alpha }{(z-z_+)(z-z_-)}\right] > 0$ for $z \in {\mathbb {C}}^+$.
- If $$\alpha > \frac{1}{9}$$ then
  $$\begin{aligned} \frac{(z-z_+)(z-z_-)}{(z- \sqrt{\alpha })^2} \in (0,\infty ) \end{aligned}$$
  if and only if $z \ne \sqrt{\alpha }$ and $z \in \left( \gamma _0 {\setminus } \Sigma _0 \right) \cup {\mathbb {R}}$.

#### Proof

(a) We consider the case $0< \alpha < 1$. Observe that $z^2 Q_{\alpha }(z)$ tends to 1 as $z \rightarrow \infty$, and there are no sign changes on the real line. Thus $z^2 Q_{\alpha }(z) \ge 0$ for real values of *z*, with double poles at $z=-1$ and $z=-\alpha$, and a local minimum at $z= \sqrt{\alpha }$. There is a minimum at $z=-\sqrt{\alpha }$ in case $\alpha \ge \frac{1}{9}$, and a local maximum at $z=-\sqrt{\alpha }$ in case $\alpha < \frac{1}{9}$. In the latter case there are local minima at $z= z_{\pm }$. It can be verified that

$$\begin{aligned} 0 \le \alpha Q_{\alpha }(-\sqrt{\alpha })< \alpha Q_{\alpha }(\sqrt{\alpha }) < 1. \end{aligned}$$

From an inspection of the graph, it follows that for any $x > \alpha Q_{\alpha }(\sqrt{\alpha })$, $x \ne 1$, there are four real solutions to the equation

6.10 $$\begin{aligned} z^2 Q_{\alpha }(z) = x. \end{aligned}$$

For $x = 1$ there are three real solutions and a solution at infinity, while for $\alpha Q_{\alpha }(-\sqrt{\alpha })< x < \alpha Q_{\alpha }(\sqrt{\alpha })$ there are two real solutions. If $\alpha \le \frac{1}{9}$, there are again four real solutions (counting multiplicities) for each $0 \le x \le \alpha Q_{\alpha }(-\sqrt{\alpha })$.

To summarize, (6.10) with $x \ge 0$ admits four solutions in ${\mathbb {R}} \cup \{\infty \}$ except in the following cases:

6.11 $$\begin{aligned} {\left\{ \begin{array}{ll} 0 \le x< \alpha Q_{\alpha }(\sqrt{\alpha }), &{} \text { and } \frac{1}{9}<\alpha< 1, \\ \alpha Q_{\alpha }(-\sqrt{\alpha })< x< \alpha Q_{\alpha }(\sqrt{\alpha }), &{} \text { and } 0<\alpha \le \frac{1}{9}. \end{array}\right. } \end{aligned}$$

and in the cases (6.11) there are only two real solutions.

On the other hand, the calculations (4.20) and (4.21) in the proof of Lemma 4.5 tell us that $z^2 Q_{\alpha }(z)$ is also real and positive for $z \in \Sigma _0$. For $\frac{1}{9}\le \alpha <1$, the function decreases from $\alpha Q_{\alpha }(\sqrt{\alpha })$ to 0 if *z* moves over $\Sigma _0$ from $\sqrt{\alpha }$ to either $z_+$ or $z_-$. Similarly, for $0 < \alpha \le \frac{1}{9}$, the function decreases from $\alpha Q_{\alpha }(\sqrt{\alpha })$ to $\alpha Q_{\alpha }(-\sqrt{\alpha })$ if *z* moves over $\Sigma _0$ from $\sqrt{\alpha }$ to $-\sqrt{\alpha }$ in either the lower or upper half plane. It means that the equation (6.10) has two additional solutions on $\Sigma _0$ precisely for the cases specified in (6.11).

Since (6.10) is a polynomial equation of degree four (if we multiply it through by the denominator) if $x \ne 1$ and of degree three if $x =1$, there are four solutions for every *x*, where we include the solution $\infty$ in case $x=1$. For $x \ge 0$ we found four solutions in $\Sigma _0 \cup \left( {\mathbb {R}} {\setminus } \{-1,-\alpha \} \right) \cup \{\infty \}$, and thus there are no other solutions in the complex plane. This proves part (a) for $0<\alpha <1$. The proof for $\alpha = 1$ is similar and easier, and we omit it.

(b) For $0< \alpha < \frac{1}{9}$ we have inequalities $z_-< - \sqrt{\alpha }< z_+ < \sqrt{\alpha }$ between the zeros and the poles and therefore

6.12 $$\begin{aligned} \frac{(z-z_+)(z-z_-)}{z^2-\alpha } = 1 + \frac{A}{z+\sqrt{\alpha }} + \frac{B}{z-\sqrt{\alpha }} \end{aligned}$$

with $A, B > 0$. Then $\mathop {\mathrm {Im}}\frac{(z-z_+)(z-z_-)}{z^2-\alpha } < 0$ for $\mathop {\mathrm {Im}}z >0$. In case $\alpha = \frac{1}{9}$ we have (6.12) with $A = 0$ and $B>0$ and again $\mathop {\mathrm {Im}}\frac{(z-z_+)(z-z_-)}{z^2-\alpha } < 0$ for $\mathop {\mathrm {Im}}z >0$. This gives (b).

(c) If $z = \sqrt{\alpha } e^{it}$ then (where we recall $z_{\pm } = \sqrt{\alpha } e^{i \theta _{\alpha }}$)

6.13 $$\begin{aligned} \frac{(z-z_+)(z-z_-)}{(z-\sqrt{\alpha })^2}&= \frac{(e^{it}- e^{i\theta _{\alpha }})(e^{it}-e^{-i\theta _{\alpha }})}{(e^{it}-1)^2} \nonumber \\&= \frac{\cos \theta _{\alpha } - \cos t}{1-\cos t}, \end{aligned}$$

which is in $(0,\frac{1+\cos \theta _{\alpha }}{2}]$ for $\theta _{\alpha } < |t| \le \pi$. The rational function in the left-hand side of (6.13) is also real and positive for real *z*, $z \ne \sqrt{\alpha }$, and admits a minimum at $z = -\sqrt{\alpha }$. Then, with an argument similar to the one we used to prove part (a), we check that these are the only *z* for which (6.13) is in $(0,\infty )$. This proves part (c). $\square$

#### Lemma 6.4

If *z* moves along $(\Sigma _{-1} \cup \Sigma _0) \cap {\mathbb {C}}^+$ from left to right, then

- $z \rightarrow \log |z|$ is strictly decreasing on $\Sigma _{-1} \cap {\mathbb {C}}^+$ and constant on $\Sigma _0 \cap {\mathbb {C}}^+$,
- $z \rightarrow \log \left| \frac{(z+1)(z+\alpha )}{z}\right|$ is stricly increasing.

#### Proof

(a) It is clear that $\log |z|$ is constant on the circle $\Sigma _0$.

Let $z=z(t)$, $t \in [0,1]$, be a smooth parametrization of $\Sigma _{-1} \cap \overline{{\mathbb {C}}^+}$ such that $z(0) = x_1$ and $z(1) = x_2$ (in the low temperature case) or $z(1) = z_+(\alpha )$ (in the high temperature case). Since $\Sigma _{-1}$ is a trajectory of the quadratic differential, $z'(t) Q_{\alpha }(z(t))^{1/2}$ is purely imaginary, and with our choice of square root, and parametrization of $\Sigma _{-1}$, we have

6.14 $$\begin{aligned} z'(t) Q_{\alpha }(z(t))^{1/2} = - i \psi (t), \qquad \text {with } \psi (t) > 0. \end{aligned}$$

Then with $$z = z(t)$$, $$0< t < 1$$,

6.15 $$\begin{aligned} \frac{d}{dt} \log |z(t)|&= \frac{d}{dt} \mathop {\mathrm {Re}}\log (z(t)) = \mathop {\mathrm {Re}}\left[ \frac{z'(t)}{z(t)} \right] \nonumber \\&= \mathop {\mathrm {Re}}\left[ \frac{- i\psi (t)}{z Q_{\alpha }(z)^{1/2}} \right] = \psi (t) \mathop {\mathrm {Im}}\left[ \frac{1}{z Q_{\alpha }(z)^{1/2}} \right] . \end{aligned}$$

By part (a) of Lemma 6.3, $z Q_{\alpha }(z)^{1/2} \not \in {\mathbb {R}}$ for $z \in {\mathbb {C}}^+ {\setminus } \Sigma _0$, and by our choice of square root we have $\mathop {\mathrm {Im}}\left[ z Q_{\alpha }(z)^{1/2} \right] > 0$ for $z \in {\mathbb {C}}^+ {\setminus } \Sigma _0$ (this can be seen from example from an expansion of $z Q_{\alpha }(z)^{1/2}$ as $z \rightarrow i \infty$), and in particular for $z \in \Sigma _{-1} \cap {\mathbb {C}}^+$. Then $\mathop {\mathrm {Im}}\left[ \frac{1}{z Q_{\alpha }(z)^{1/2}} \right] < 0$, and we find from (6.15) with $\psi (t) > 0$ that $\frac{d}{dt} \log |z(t)| < 0$. This proves part (a).

(b) Let *z*(*t*), $t \in [0,1]$ be a smooth parametrization of $\Sigma _{-1} \cap {\mathbb {C}}^+$ as in the proof of part (a). Let $\psi (t) > 0$ be as in (6.14). Then with $z=z(t)$,

6.16 $$\begin{aligned} \frac{d}{dt}\log \left| \frac{(z(t)+1)(z(t)+\alpha )}{z(t)}\right|&=\mathop {\mathrm {Re}}\left[ \left( \frac{1}{z+1} + \frac{1}{z+ \alpha }-\frac{1}{z} \right) z'(t) \right] \nonumber \\&= \psi (t) \mathop {\mathrm {Im}}\left[ \left( \frac{z^2-\alpha }{z(z+1)(z+ \alpha )}\right) \frac{1}{Q_{\alpha }(z)^{1/2}} \right] . \end{aligned}$$

If $$0 < \alpha \le \frac{1}{9}$$, then

$$\begin{aligned} \left( \frac{z^2-\alpha }{z(z+1)(z+ \alpha )}\right) \frac{1}{Q_{\alpha }(z)^{1/2}} = \frac{z^2-\alpha }{(z-z_+)(z-z_-)} \end{aligned}$$

and this has positive imaginary part for $z \in \Sigma _{-1} \cap {\mathbb {C}}^+$ by part (b) of Lemma 6.3.

If $$\frac{1}{9} < \alpha \le 1$$ then

$$\begin{aligned} \left( \frac{z^2-\alpha }{z(z+1)(z+ \alpha )}\right) \frac{1}{Q_{\alpha }(z)^{1/2}} = \frac{z- \sqrt{\alpha }}{((z-z_+)(z-z_-))^{1/2}}. \end{aligned}$$

By part (c) of Lemma 6.3, this cannot be real for $z \in {\mathbb {C}}^+ {\setminus } \{\sqrt{\alpha } e^{it} \mid \theta _{\alpha } \le |t| \le \pi \}$, since otherwise its square would be $>0$ and that would contradict the statement of the lemma. It follows that the sign of its imaginary part is piecewise constant on ${\mathbb {C}}^+ {\setminus } \gamma _{0}$ (recall that $Q_{\alpha }(z)^{1/2}$ is discontinuous along $\Sigma _{0}$). It is in fact $> 0$ on the outer component, and this includes $(\Sigma _{-1} {\setminus } \{z_+\}) \cap {\mathbb {C}}^+$.

Thus in both cases we find that (6.16) is positive for $0< t < 1$, and therefore $z \mapsto \log \left| \frac{(z+1)(z+\alpha )}{z}\right|$ increases along $\Sigma _{-1} \cap {\mathbb {C}}^+$ as claimed in part (b).

The increase along $\Sigma _0 \cap {\mathbb {C}}^+$ is immediate, since both $z \mapsto |z+1|$ and $z \mapsto |z+\alpha |$ are strictly increasing if *z* moves along the circle $\Sigma _0$ from $-\sqrt{\alpha }$ to $\sqrt{\alpha }$, while $z \mapsto |z|$ is constant. $\square$

#### Corollary 6.5

Suppose $\eta \le \frac{\xi }{2} < 0$. Then $z \mapsto \mathop {\mathrm {Re}}\Phi _{\alpha }(z)$ is strictly decreasing as *z* traverses $(\Sigma _{-1} \cup \Sigma _0) \cap {\mathbb {C}}^+$ from left to right.

#### Proof

Indeed, from the definition (6.4) and the fact that $\mathop {\mathrm {Re}}\phi = 0$ on $\Sigma _{-1}$ and $\Sigma _0$, we obtain for $z \in \Sigma _{-1} \cup \Sigma _0$,

6.17 $$\begin{aligned} \mathop {\mathrm {Re}}\Phi _{\alpha }(z)&= \frac{\xi }{2} \log |(z+1)(z+\alpha )| - \eta \log |z| \nonumber \\&= \frac{\xi }{2} \log \left| \frac{(z+1)(z+\alpha )}{z} \right| + \left( \frac{\xi }{2} - \eta \right) \log |z|, \end{aligned}$$

and by Lemma 6.4 the sum at the right-hand-side of (6.17) is strictly decreasing since $\xi < 0$ and $\frac{\xi }{2} - \eta \ge 0$. $\square$

Due to Corollary 6.5, we see that the level set (6.9) has at most one point of intersection with $(\Sigma _{-1} \cup \Sigma _0) \cap {\mathbb {C}}^+$, because $\mathop {\mathrm {Re}}\Phi _{\alpha }$ is strictly decreasing there. Therefore at least three of the $\Gamma _j$’s, say $\Gamma _1, \Gamma _2, \Gamma _3$, do not intersect $(\Sigma _{-1} \cup \Sigma _0) \cap {\mathbb {C}}^+$, which means that they have to go to the real line inside the domain enclosed by $\Sigma _{-1} \cup \Sigma _0$ (or inside the disk bounded by $\Sigma _0$ in the low temperature regime), and then by symmetry end at $\overline{s}$ inside that domain. Taking $p_j \in \Gamma _j \cap {\mathbb {R}}$ for $j=1,2,3$, we choose the ordering of the $\Gamma _j$’s such that $p_1< p_2 < p_3$.

The contours $\Gamma _1$, $\Gamma _2$, $\Gamma _3$ enclose two bounded domains for which $\mathop {\mathrm {Re}}\Phi _{\alpha }$ is constant on the boundaries and harmonic inside, except at the singularities $-1$, $-\alpha$, 0, where $\mathop {\mathrm {Re}}\Phi _{\alpha }$ is unbounded by (6.7). By the maximum principle for harmonic functions, each of the two domains has to contain at least one of the singularities. Also $\mathop {\mathrm {Re}}(\Phi _{\alpha } -\Phi _{\alpha }(s))$ has opposite signs on the two bounded domains. Then again by (6.7) one domain contains 0 and the other domain contains $-\alpha$, and possibly also $-1$, since at both these points $\mathop {\mathrm {Re}}\Phi _{\alpha }$ tends to $-\infty$. Thus

$$\begin{aligned} p_1< -\alpha< p_2< 0< p_3 < \sqrt{\alpha }. \end{aligned}$$

If $\Gamma _4$ would remain inside $\Sigma _{-1} \cup \Sigma _0$ as well, then it would also go to the real line, say at a point $p_4$, and continue to $\overline{s}$ inside this domain. If $p_3< p_4 < \sqrt{\alpha }$ then $\Gamma _4$ and $\Gamma _3$ would enclose a domain with $\mathop {\mathrm {Re}}\Phi _{\alpha }$ is constant on the boundary, and harmonic inside, and we would have a contradiction with the maximum principle. If $p_4 < p_1$ then $\Gamma _4$ and $\Gamma _1$ enclose a bounded domain within and we find a contradiction in the same way.

Thus $\Gamma _4$ comes to $(\Sigma _{-1} \cup \Sigma _0) \cap {\mathbb {C}}^+$ and continues into the outer domain of ${\mathbb {C}} {\setminus } {\mathcal {N}}_{\phi }$. It cannot go to infinity because of (6.7) and so it has to go to the real line at a point $p_4$ and by symmetry it continues in the lower half plane where it crosses $\Sigma _{-1} \cup \Sigma _0$ again and ends at $\overline{s}$.

As $\mathop {\mathrm {Re}}\Phi$ decreases along $(\Sigma _{-1}\cup \Sigma _0) \cap {\mathbb {C}}^+$ from left to right, we find $\mathop {\mathrm {Re}}\Phi _{\alpha }(\sqrt{\alpha }) < \mathop {\mathrm {Re}}\Phi _{\alpha }(s)$. Since $\mathop {\mathrm {Re}}\Phi _{\alpha }(z) \rightarrow +\infty$ as $z \rightarrow \infty$, the level set ${\mathcal {N}}_{\Phi }$ intersects the real line at a point $> \sqrt{\alpha }$. This can only be at $p_4$. Thus $\Gamma _4$ and $\Gamma _3$ enclose a domain where $\mathop {\mathrm {Re}}\Phi _{\alpha } < \mathop {\mathrm {Re}}\Phi _{\alpha }(s)$ and that contains (part of) $\Sigma _0$ where $\Phi _{\alpha }$ has its branch cut, and where $\mathop {\mathrm {Re}}\Phi _{\alpha }$ is not harmonic. Hence there is no contradiction with the maximum principle.

To summarize, we have a situation as in Fig. 14 in case $p_1 < -1$, or as in Fig. 15 if $-1< p_1 < -\alpha$. In the latter case, there is also a separate part $\Gamma _5$ of ${\mathcal {N}}_{\Phi }$ that goes around $-1$.

Figures 14 and 15 are for the high temperature regime. In the low temperature regime we have that $\Sigma _0$ is the full circle of radius $\sqrt{\alpha }$. Then in the above discussion we can replace $\Sigma _{-1} \cup \Sigma _0$ by $\Sigma _0$. It follows that $\Gamma _1$, $\Gamma _2$, $\Gamma _3$ stay inside the disk of radius $\sqrt{\alpha }$, and so $\Gamma _1$ does not go around $-1$. There is always a part $\Gamma _5$ going around $-1$ in the low temperature regime, as shown in Fig. 16.

**Fig. 17:**
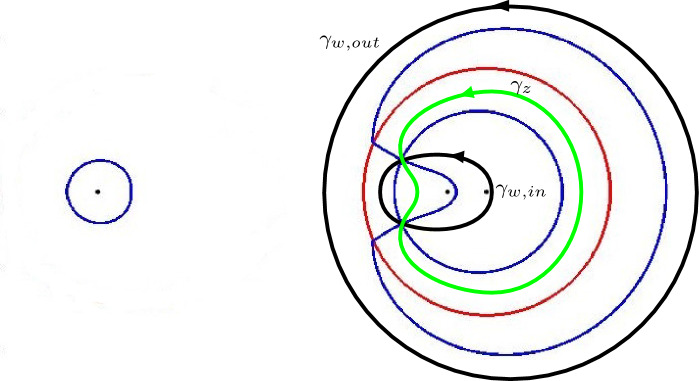
Contours $\gamma _z$ (green), $\gamma _{w,out}$ (black), and $\gamma _{w,in}$ (black) in the low temperature regime. The contours satisfy the conditions of Corollary 6.6(a) and Proposition 7.8.

**Fig. 18:**
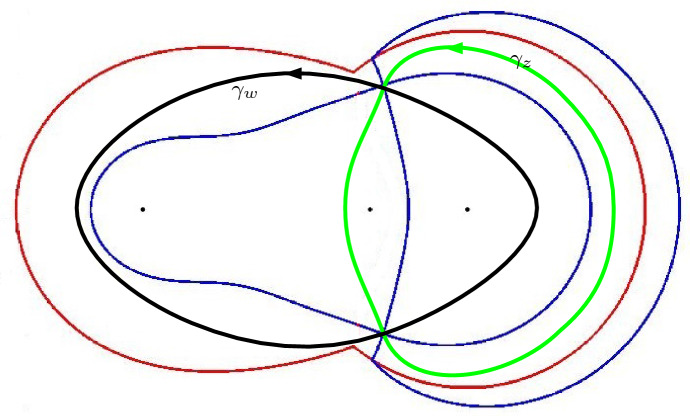
The contours $\gamma _z$ (green) and $\gamma _w$ (black) in the high temperature regime. The contours satisfy the conditions of Corollary 6.6 (b) and Proposition 7.9

It is now clear that we can find contours as described next. See also Figs. 17 and 18 below.

#### Corollary 6.6

Let $$(\xi ,\eta ) \in {\mathcal {L}}_{\alpha }$$ with $$\eta \le \frac{\xi }{2} < 0$$.

- In the low temperature regime there are closed contours $\gamma _z$ and $\gamma _{w,in}$, $\gamma _{w,out}$ such that
  $\gamma _{w,out}$ lies outside the circle $\gamma _0$, does not go around $-1$, and is such that
    $$\begin{aligned} \mathop {\mathrm {Re}}\Phi _{\alpha }(w) > \mathop {\mathrm {Re}}\Phi _{\alpha }(s), \qquad w \in \gamma _{w,out}. \end{aligned}$$
  $\gamma _{w,in}$ lies inside the circle $\gamma _0$, goes around $-\alpha$, and it passes through *s* and $\overline{s}$ in such a way that
    $$\begin{aligned} \mathop {\mathrm {Re}}\Phi _{\alpha }(w) > \mathop {\mathrm {Re}}\Phi _{\alpha }(s), \qquad w \in \gamma _{w,in} {\setminus } \{s, \overline{s}\}. \end{aligned}$$
  $\gamma _z$ lies inside the circle $\gamma _0$, goes around 0, and it passes through *s* and $\overline{s}$ in such a way that
    $$\begin{aligned} \mathop {\mathrm {Re}}\Phi _{\alpha }(z) < \mathop {\mathrm {Re}}\Phi _{\alpha }(s), \qquad z \in \gamma _{z} {\setminus } \{s, \overline{s}\}. \end{aligned}$$
- In the high temperature regime there exist contours $\gamma _z$ and $\gamma _w$ such that
  $\gamma _{w}$ lies in the domain bounded by $\Sigma _0 \cup \Sigma _{-1}$, it goes around $-1$, and it passes through *s* and $\overline{s}$ in such a way that
    $$\begin{aligned} \mathop {\mathrm {Re}}\Phi _{\alpha }(w) > \mathop {\mathrm {Re}}\Phi _{\alpha }(s), \qquad w \in \gamma _{w} {\setminus } \{s, \overline{s}\}, \end{aligned}$$
  $\gamma _z$ lies inside the circle $\gamma _0$, goes around 0, and it passes through *s* and $\overline{s}$ in such a way that
    $$\begin{aligned} \mathop {\mathrm {Re}}\Phi _{\alpha }(z) < \mathop {\mathrm {Re}}\Phi _{\alpha }(s), \qquad z \in \gamma _{z} {\setminus } \{s, \overline{s}\}. \end{aligned}$$

In the low temperature regime we will also use $\gamma _w = \gamma _{w,in} \cup \gamma _{w,out}$.

## Analysis of Double Contour Integrals

### Lozenge probabilities

In the final part of the analysis we are going to deform contours in the double contour integral to the ones from Corollary 6.6, which leads to the proof of Theorem 2.5. We start by expressing the probabilities for the three types of lozenges as double contour integrals.

We use *F*(*z*; *x*, *y*) as in (6.3) and for a function $(w,z) \mapsto H(w,z)$,

7.1 $$\begin{aligned} {\mathcal {I}}_N(x,y;H) =&\frac{1}{(2\pi i)^2} \oint _{\gamma _0} \oint _{\gamma _0} R_N(w,z) \frac{(w+1)^N(w+\alpha )^N}{w^{2N}} \frac{F(z;x,y)}{F(w;x,y)} H(w,z) dw dz \end{aligned}$$

We will use (7.1) only for functions $(w,z) \mapsto H(w,z)$ that are products of a rational function in *w* and a rational function in *z*, both with poles at $-1$, $-\alpha$, and 0 only. In addition, the integrand in (7.1) will have singularities for $w=0$ and $z=0$ only, and the contour $\gamma _0$ can be deformed to an arbitrary closed contour around 0, and we can take different contours for the two integrals.

#### Theorem 7.1

The following statements hold:7.27.37.4with ${\mathcal {I}}_N(x,y;H)$ as in (7.1), and

7.5 $$\begin{aligned} \begin{aligned} H_{1,even}(w,z)&= \frac{w}{z(w+\alpha )}, \quad&H_{1,odd}(w,z)&= \frac{w}{z(w+1)}, \\ H_{2,even}(w,z)&= \frac{\alpha }{z(w+\alpha )},&H_{2,odd}(w,z)&= \frac{1}{z(w+1)}, \\ H_3(w,z)&= \frac{1}{z}. \end{aligned} \end{aligned}$$

The formula (7.4) is immediate from the formula (1.7) for the correlation kernel, since $K_N(x,y,x,y)$ is the probability to have a path at $(x,y + \tfrac{1}{2})$ which is the same as the probability to have either a type I or type II lozenge at the location (*x*, *y*). Hence $1- K_N(x,y,x,y)$ is the probability to have a type III lozenge at location (*x*, *y*) which is (7.1) with $H(w,z) = H_3(w,z) = \frac{1}{z}$. The point of Theorem 7.1 is that there exist similar double contour integrals for the other two probabilities.

The proof of Theorem 7.1 relies on two lemmas. We start by defining the height function $h:\{0,\ldots ,2N\}\times {\mathbb {Z}} \rightarrow {\mathbb {N}}$ in terms of the paths $\pi _j:\{0,1,\ldots ,2N\} \rightarrow \mathbb {Z}+\tfrac{1}{2}$, for $j=1,\ldots ,2N$, by

$$\begin{aligned} h(x,y)= \#\{ j \mid \pi _j(x)< y \}. \end{aligned}$$

The graph of *h* is a stepped surface and the paths can be thought of as level curves of this random surface. We can recover the tiling from the height function by using simple identities which relate the positions of the different lozenges to differences of the height function.

#### Lemma 7.2

The following identities hold:

#### Proof

The proof is straightforward. $\square$

The next step is a double integral formula for the expectation value of the height function.

#### Lemma 7.3

For $$(x, y) \in \{0,1,\ldots , 2N\} \times {\mathbb {Z}}$$,

$$\begin{aligned} {\mathbb {E}}[h(x,y)]= & {} \sum _{k < y} K_N(x,k,x,k) = \frac{1}{(2\pi i)^2} \oint _{\tilde{\gamma }}\oint _\gamma R_N(w,z)\\&\quad \frac{(w+1)^N(w+\alpha )^N }{w^{2N}} \frac{F(z;x,y)}{F(w;x,y)} \frac{dw dz}{w-z}. \end{aligned}$$

where $\tilde{\gamma }$ is deformation of $\gamma$ such that $|z|<|w|$ whenever $z \in \tilde{\gamma }$ and $w \in \gamma$.

#### Proof

By the determinantal structure of the correlations (see Proposition 1.1) we have

$$\begin{aligned} {\mathbb {E}}[h(x,y)]= \sum _{k < y} K_N(x,k,x,k). \end{aligned}$$

After inserting the expression (1.7) for the kernel, bringing the sum inside the integrals, and evaluating the geometric series $\displaystyle \frac{1}{z}\sum \nolimits _{k < y} \frac{w^k}{z^k} = \frac{w^y}{z^y} \frac{1}{w-z}$ for $|z|<|w|$, we obtain the statement. $\square$

Now we are ready for the proof of Theorem 7.1.

#### Proof of Theorem 7.1

Lemma 7.2 implies thatWe insert the double contour integral formula of Lemma 7.3 and combine the two integrals by subtracting the two integrands. Since

$$\begin{aligned} \left( \frac{F(z;x,y+1)}{F(w;x,y+1)} - \frac{F(z;x+1,y+1)}{F(w;x+1,y+1)} \right) \frac{1}{w-z} = \frac{F(z;x,y)}{F(w;x,y)} \times {\left\{ \begin{array}{ll} \frac{w}{z(w+\alpha )}, &{} \text { if }x\text { is even},\\ \frac{w}{z(w+1)}, &{} \text { if }x\text { is odd}, \end{array}\right. } \end{aligned}$$

which we can check from (6.3) separately for *x* even or odd, the formula (7.2) follows. Note also that the pole at $z=w$ disappeared when we took the difference, and therefore $\tilde{\gamma }$ can be moved back to $\gamma$ in (7.2).

The proof of (7.3) is similar, and (7.4) is immediate from the structure of the determinantal point process, as already noted after the statement of Theorem 7.1. $\square$

### Symmetries

We use symmetries in the double integral (7.1) to be able to restrict attention to the lower left part of the hexagon.

#### Proposition 7.4

The double integral (7.1) has symmetries under the mappings $(x,y) \mapsto (2N-x,2N-y)$ and $(x,y) \rightarrow (x,N+x-y)$ as follows.

- We have
  7.6 $$\begin{aligned} {\mathcal {I}}_N(2N-x,2N-y; H)&= {\mathcal {I}}_N(x,y; \widehat{H}), \end{aligned}$$
  with
  7.7 $$\begin{aligned} \widehat{H}(w,z) = H(z,w) \times {\left\{ \begin{array}{ll} 1, &{} \text { if }x\text { is even}, \\ \frac{w+\alpha }{w+1} \frac{z+1}{z+\alpha }, &{} \text { if }x\text { is odd}. \end{array}\right. } \end{aligned}$$
- We have
  7.8 $$\begin{aligned} {\mathcal {I}}_N(x,N+x-y; H)&= {\mathcal {I}}_N(x,y; \widetilde{H}) \end{aligned}$$
  with
  7.9 $$\begin{aligned} \widetilde{H}(w,z) = \frac{\alpha }{wz} H\left( \frac{\alpha }{w},\frac{\alpha }{z}\right) \times {\left\{ \begin{array}{ll} 1, &{} \text { if }x\text { is even}, \\ \frac{w+\alpha }{w+1} \frac{z+1}{z+\alpha }, &{} \text { if }x\text { is odd}. \end{array}\right. } \end{aligned}$$

#### Proof

(a) From (6.3) we deduce

$$\begin{aligned} F(z;2N-x,2N-y) = \frac{(z+1)^N (z+\alpha )^N}{z^{2N}} F(z;x,y)^{-1} \times {\left\{ \begin{array}{ll} 1 &{} \text { if }x\text { is even}, \\ \frac{z+\alpha }{z+1} &{} \text { if }x\text { is odd}. \end{array}\right. } \end{aligned}$$

We insert this in the double integral (7.1) with $(2N-x,2N-y)$ instead of (*x*, *y*), and we interchange variables $(w,z) \mapsto (z,w)$. Since $R_N(w,z)$ is a symmetric expression in the two variables, the identity (7.6) with $\widehat{H}$ given by (7.7) follows.

(b) We now apply the change of variables $w \mapsto \frac{\alpha }{w}$, $z \mapsto \frac{\alpha }{z}$ to the integral (7.1) with $(x,N+x-y)$ instead of (*x*, *y*). Then $R_N(w,z)$ transforms as in (7.11) which we will prove in a separate lemma below. The other factors in the integrand of (7.1) transform as

$$\begin{aligned} \frac{(w+1)^N (w+\alpha )^N}{w^{2N}}&\mapsto \alpha ^{-N} (w+1)^N (w+\alpha )^N \\ H(w,z) dwdz&\mapsto H \left( \frac{\alpha }{w}, \frac{\alpha }{z} \right) \frac{\alpha ^2}{w^2 z^2} dwdz \\ F(z; x, N+x-y)&\mapsto \alpha ^{-N - \lfloor \frac{x}{2} \rfloor + y} z^N F(z;x,y) \times {\left\{ \begin{array}{ll} 1, &{} \text {if }x\text { is even} \\ \frac{z+1}{z+\alpha }, &{} \text {if }x\text { is odd} \end{array}\right. } \end{aligned}$$

and similarly for $F(w;x,N+x-y)$. Combining all the factors we arrive at (7.8) with $\widetilde{H}$ as in (7.9). Finally, each transformation reverses the orientation of the respective contour. We change the orientation of each contour back to the original one at the cost of a minus sign and since we do to this two times the minus signs cancel against each other. $\quad \square$

In the proof of part (b) of Proposition 7.4 we needed an identity for $R_N$ that we prove in a separate lemma. It is related to a symmetry in the Riemann–Hilbert problem 5.2.

#### Lemma 7.5

- Let $\gamma = \gamma _{0}$ be the circle centered at 0 of radius $\sqrt{\alpha }$. Then the following symmetry holds
  7.10 $$\begin{aligned} Y(z) = \begin{pmatrix} \alpha ^{\frac{N}{2}} &{} 0 \\ 0 &{} -\alpha ^{-\frac{N}{2}} \end{pmatrix} Y(0)^{-1} Y\left( \frac{\alpha }{z}\right) \begin{pmatrix} z^{N} \alpha ^{-\frac{N}{2}} &{} 0 \\ 0 &{} - z^{-N} \alpha ^{\frac{N}{2}} \end{pmatrix}. \end{aligned}$$
- The Christoffel–Darboux kernel $R_N$ satisfies
  7.11 $$\begin{aligned} R_{N}\left( \frac{\alpha }{w},\frac{\alpha }{z}\right) = \frac{\alpha ^{N-1}}{w^{N-1} z^{N-1}} R_{N}(w,z), \qquad w,z \in {\mathbb {C}} {\setminus } \{0\}. \end{aligned}$$

#### Proof

Part (a) follows since the right-hand side of (7.10) satisfies the conditions of the RH problem 5.2, as can be check by straightforward calculations, and the uniqueness of the solution of the RH problem.

Part (b) follows after inserting (7.10) into (5.7), again with simple calculations. $\square$

There are corresponding symmetries for the location of the saddle point.

#### Proposition 7.6

Let $(\xi ,\eta ) \in {\mathcal {L}}_{\alpha }$. Then also $(-\xi ,-\eta ) \in {\mathcal {L}}_{\alpha }$, $(\xi ,\xi -\eta ) \in {\mathcal {L}}_{\alpha }$ and

7.12 $$\begin{aligned} s(-\xi ,-\eta ;\alpha )&= s(\xi ,\eta ;\alpha ) \end{aligned}$$

7.13 $$\begin{aligned} s(\xi ,\xi -\eta ;\alpha )&= \alpha \left( \overline{s(\xi ,\eta ;\alpha )} \right) ^{-1} \end{aligned}$$

#### Proof

From (6.5), we have

$$\begin{aligned} \Psi _{\alpha }(z; \xi ,\eta ) = - \Phi _{\alpha }(z;-\xi ,-\eta ) \end{aligned}$$

and this implies (7.12).

It can be readily verified from (2.5) and (2.7) that $\frac{\alpha ^2}{z^4} Q_{\alpha } \left( \frac{\alpha }{z} \right) = Q_{\alpha } (z)$. Noting that $\phi '(z) = \pm Q_{\alpha }(z)^{1/2}$ by (4.15) and (4.16) and keeping track of the signs of the square roots, we obtain from this

$$\begin{aligned} - \frac{\alpha }{z^2} \phi '\left( \frac{\alpha }{z} \right) = \phi '(z) \end{aligned}$$

Also, a straightforward computation shows that

$$\begin{aligned} - \frac{\alpha }{z^2} \left[ \frac{\xi }{2} \left( \frac{1}{z+1} + \frac{1}{z+\alpha }\right) - \frac{\eta }{z} \right] _{z \mapsto \frac{\alpha }{z}} = \frac{\xi }{2} \left( \frac{1}{z+1} + \frac{1}{z+\alpha }\right) - \frac{\xi -\eta }{z}. \end{aligned}$$

From (6.4) and (6.5) and the last two equalities, we then find

$$\begin{aligned} -\frac{\alpha }{z^2} \Phi _{\alpha }'\left( \frac{\alpha }{z}; \xi ,\eta \right) = \Phi _{\alpha }'(z; \xi , \xi -\eta ) \end{aligned}$$

and similarly for $\Psi _{\alpha }$. This gives (7.13), since by definition $s(\xi ,\xi -\eta ;\alpha )$ is the saddle that is in the upper half plane, and therefore the complex conjugation appears in (7.13). $\quad \square$

### Preliminaries to the asymptotic analysis

Theorem 2.5 will follow from Theorem 7.1 and the following result.

#### Proposition 7.7

Let $0 < \alpha \le 1$. Suppose $x, y \in {\mathbb {N}}$ vary with *N* such that (2.1) holds with $(\xi ,\eta ) \in {\mathcal {L}}_{\alpha }$. Let $(w,z) \mapsto H(w,z)$ satisfy the conditions stated after the definition (7.1). Then ${\mathcal {I}}_N(x,y; H)$ from (7.1) has the limit

7.14 $$\begin{aligned} \lim _{N \rightarrow \infty } {\mathcal {I}}_N(x,y;H) = \frac{1}{2\pi i} \int _{\overline{s}}^s H(z,z) dz \end{aligned}$$

where $s = s(\xi ,\eta ;\alpha )$ and the integration path from $\overline{s}$ to *s* in (7.14) is in ${\mathbb {C}} {\setminus } (-\infty ,0]$.

The integrals (7.14) are easy to calculate if *H* is one of the functions from (7.5). For $H = H_{1,even}$, we obtain for example

$$\begin{aligned} \frac{1}{2\pi i} \int _{\overline{s}}^s H_{1,even}(z,z) dz&= \frac{1}{2\pi i} \int _{\overline{s}}^s \frac{dz}{z+\alpha } = \frac{1}{2\pi i} \left[ \log (s+\alpha ) - \log (\overline{s} + \alpha ) \right] \\&= \frac{1}{\pi } \arg (s+\alpha ). \end{aligned}$$

Clearly, $\arg (s+\alpha )$ is equal to the angle $\psi _1$ in the triangle $T_{\alpha }$ of Fig. 5. Thus (2.14) with *x* even follows from (7.2) and Proposition 7.7. The other limits in Theorem 2.5 follow in a similar fashion. Therefore we have reduced the proof of Theorem 2.5 to the proof of Proposition 7.7.

The symmetries from Proposition 7.4 allow us to restrict our attention to $(\xi , \eta ) \in {\mathcal {L}}_{\alpha }$ with $\eta \le \frac{\xi }{2} \le 0$.

Indeed, suppose that we can prove Proposition 7.7 for certain $(\xi ,\eta ) \in {\mathcal {L}}_{\alpha }$. Let (*x*, *y*) vary with *N* such that (2.1) hold but with limits $(\xi ,\xi -\eta ) \in {\mathcal {L}}_{\alpha }$. Suppose *H* satisfies the conditions of Proposition 7.7. Then by (7.8)

$$\begin{aligned} \lim _{N \rightarrow \infty } {\mathcal {I}}_N(x,y; H)&= \lim _{N \rightarrow \infty } {\mathcal {I}}_N(x,N+x-y; \widetilde{H}) \\&= \frac{1}{2\pi i} \int _{\overline{s}}^s \widetilde{H}(z,z) dz, \qquad s = s(\xi ,\eta ;\alpha ), \end{aligned}$$

since also $\widetilde{H}$ satisfies the conditions of Proposition 7.7, and by assumption Proposition 7.7 holds for $(\xi ,\eta )$. Using (7.9) and after changing variables $\frac{\alpha }{z} \mapsto z$, we find

$$\begin{aligned} \lim _{N \rightarrow \infty } {\mathcal {I}}_N(x,y; H)&= \frac{1}{2\pi i} \int _{\overline{s}}^s \frac{\alpha }{z^2} H\left( \frac{\alpha }{z}, \frac{\alpha }{z} \right) dz \\&= \frac{1}{2\pi i} \int _{\alpha s^{-1}} ^{\alpha (\overline{s})^{-1} } H(z,z) dz, \qquad s = s(\xi ,\eta ;\alpha ). \end{aligned}$$

We finally use (7.13) and we find (7.14) with $s = s(\xi ,\xi -\eta ;\alpha )$. Thus Proposition 7.7 holds for $(\xi ,\xi -\eta )$ if it holds for $(\xi ,\eta )$.

Similarly, but now using (7.6)–(7.7) and (7.12), we find that Proposition 7.7 holds for $(-\xi ,-\eta )$ if it holds for $(\xi ,\eta )$, and by combining the two arguments, it also holds for $(-\xi , -\xi +\eta )$.

Thus in order to prove Proposition 7.7 it suffices to do it for $(\xi ,\eta ) \in {\mathcal {L}}_{\alpha }$ with $\eta \le \frac{\xi }{2} \le 0$. We focus on the case $\eta \le \frac{\xi }{2} < 0$ and give full arguments there. The case $\xi = 0$ is special since it means that the saddle $s(\xi ,\eta ;\alpha )$ is on the branch cut $\Sigma _0$. It can be handled as a limiting case with the help of additional contour deformations.

### Contour deformations

#### Contour deformation in the low temperature regime

We start the analysis of the double integral (7.1) with a contour deformation. There are several ways to deform the contours, and the ones we are going to present will be useful for the lower left part of the liquid region, that is for $(\xi ,\eta ) \in {\mathcal {L}}_{\alpha }$ with $\eta \le \xi /2 < 0$ as in Corollary 6.6. The deformations will be different for the low and high temperature regimes.

##### Proposition 7.8

Let $0 < \alpha \le \frac{1}{9}$ and $(\xi ,\eta ) \in {\mathcal {L}}_{\alpha }$ with $\eta< \frac{\xi }{2} <0$. Let $\gamma _z$, $\gamma _{w,in}$ and $\gamma _{w,out}$ be closed contours as in Corollary 6.6 (a), (see also Fig. 17). Then (7.1) is equal to

7.15 $$\begin{aligned} {\mathcal {I}}_N(x,y;H)= & {} \frac{1}{2\pi i} \int _{\overline{s}}^s H(z,z) dz + \frac{1}{(2\pi i)^2} \oint _{\gamma _z} dz \oint _{\gamma _{w,in}} \frac{dw}{w-z} {\mathcal {R}}_{N}(w,z) \frac{F(z; x,y)}{F(w;x,y)} H(w,z) \nonumber \\&- \frac{1}{(2\pi i)^2} \oint _{\gamma _z} dz \oint _{\gamma _{w,out}} \frac{dw}{w-z} {\mathcal {R}}_{N}(w,z) \frac{F(z; x,y)}{F(w;x,y)} H(w,z) \end{aligned}$$

where ${\mathcal {R}}_N$ is given by (5.8) and *F* is given by (6.3).

##### Proof

In (7.1) we use $\gamma _z$ for the integral with respect to the *z* variable, and $\gamma _0$ (initially) for the *w* variable. By the conditions in Corollary 6.6 (a), the contour $\gamma _z$ lies inside $\gamma _0$.

By Sokhotskii-Plemelj formula and (5.8) we have for $w \in \gamma _0$,

$$\begin{aligned} R_N(w,z) \frac{(w+1)^N (w+\alpha )^N}{w^{2N}} (w-z) = {\mathcal {R}}_{N,+}(w,z) - {\mathcal {R}}_{N,-}(w,z) \end{aligned}$$

where the ± boundary values are with respect to the *w* variable. This we substitute into the double integral (7.1) to obtain the difference of two double integrals,

$$\begin{aligned}&\frac{1}{(2\pi i)^2} \oint _{\gamma _z} dz \oint _{\gamma _0} \frac{dw}{w-z} {\mathcal {R}}_{N,+}(w,z) \frac{F(z;x,y)}{F(w;x,y)} H(w,z) \\&\quad - \frac{1}{(2\pi i)^2} \oint _{\gamma _z} dz \oint _{\gamma _0} \frac{dw}{w-z} {\mathcal {R}}_{N,-}(w,z) \frac{F(z;x,y)}{F(w;x,y)} H(w,z). \end{aligned}$$

We deform $\gamma _0$ inwards to $\gamma _{w,in}$ in the first double integral and outwards to $\gamma _{w,out}$ in the second double integral. (Recall that $+$-side refers to the interior of $\gamma _0$ and −-side to its exterior.)

We do not encounter any singularites of the integrand if we do the deformation into the exterior domain, since by assumption $\gamma _{w,out}$ does not go around $-1$. Thus by Cauchy’s theorem we obtain the last term in (7.15).

In the deformation of the first integral we pick up residue contributions for those $z \in \gamma _z$ that are in the exterior of $\gamma _{w,in}$. This is due to the pole at $w=z$ that we encounter when deforming $\gamma _0$ into $\gamma _{w,in}$. Since ${\mathcal {R}}_N(z,z) = 1$, the contribution of the poles leads to the first term in (7.15). The remaining double integral is the second term in (7.15). $\square$

#### Contour deformation in the high temperature regime

In the second proposition (relevant for the high temperature case) we modify the definition (5.8). We use a large circle $\gamma _{\rho }$ centered at the origin of radius $\rho > 10$ and define

7.16 $$\begin{aligned} \widetilde{{\mathcal {R}}}_{N}(w,z) = \frac{1}{2\pi i} \oint _{\gamma _{\rho }} R_N(s,z) \frac{(s+1)^N(s+\alpha )^N}{s^{2N}} \frac{s-z}{s-w} ds. \end{aligned}$$

Note that (7.16) coincides with (5.8) for *w* inside $\gamma _0$, and it is the analytic continuation (in the *w* variable) of (5.8) with $|w|<\alpha$ to the disk $|w| < \rho$. Because of (5.13) and the jump (5.11) of *T*, we have

7.17 $$\begin{aligned} \widetilde{{\mathcal {R}}}_N(w,z) = {\left\{ \begin{array}{ll} \begin{pmatrix} 1 &{} 0 \end{pmatrix} T^{-1}(w) T(z) \begin{pmatrix} 1 \\ 0 \end{pmatrix} e^{N(g(z)-g(w))}, &{} \quad |w|< \sqrt{\alpha }, \\ \begin{pmatrix} 1 &{} - e^{2N \phi (z)} \end{pmatrix} T^{-1}(w) T(z) \begin{pmatrix} 1 \\ 0 \end{pmatrix} e^{N(g(z)-g(w))},&\quad \sqrt{\alpha }< |w| < \rho , \end{array}\right. } \end{aligned}$$

##### Proposition 7.9

Let $\frac{1}{9}< \alpha < 1$ and $(\xi ,\eta ) \in {\mathcal {L}}_{\alpha }$ with $\eta \le \frac{\xi }{2} <0$. Suppose $\gamma _z$ and $\gamma _{w}$ are closed contours as in Corollary 6.6 (b), (see also Fig. 18). Let (*x*, *y*) be coordinates inside the hexagon. Then the double contour integral (7.1) is equal to

7.18 $$\begin{aligned} {\mathcal {I}}_N(x,y;H) = \frac{1}{2\pi i} \int _{\overline{s}}^s H(z,z) dz + \frac{1}{(2\pi i)^2} \oint _{\gamma _z} dz \oint _{\gamma _{w}} \frac{dw}{w-z} \widetilde{{\mathcal {R}}}_{N}(w,z) \frac{F(z; x,y)}{F(w;x,y)} H(w,z), \end{aligned}$$

where $\widetilde{{\mathcal {R}}}_N$ is given by (7.16) and *F* is given by (6.3).

##### Proof

As in the proof of Proposition 7.8 we have (but now we use (7.16))

$$\begin{aligned} R_N(w,z) \frac{(w+1)^N (w+\alpha )^N}{w^{2N}} (w-z) = \widetilde{{\mathcal {R}}}_{N,+}(w,z) - \widetilde{{\mathcal {R}}}_{N,-}(w,z) \end{aligned}$$

with $w \in \gamma _\rho$, and the ± boundary values are for $w \in \gamma _\rho$.

We choose $\gamma _\rho$ for the contour in the *w* integral in (7.1) and $\gamma _z$ for the *z* integral. Then the double contour integral is a difference of two double integrals

7.19 $$\begin{aligned}&\frac{1}{(2\pi i)^2} \oint _{\gamma _z} dz \oint _{\gamma _\rho } \frac{dw}{w-z} \widetilde{{\mathcal {R}}}_{N,+}(w,z) \frac{F(z;x,y)}{F(w;x,y)} H(w,z) \nonumber \\&\quad - \frac{1}{(2\pi i)^2} \oint _{\gamma _z} dz \oint _{\gamma _\rho } \frac{dw}{w-z} \widetilde{{\mathcal {R}}}_{N,-}(w,z) \frac{F(z;x,y)}{F(w;x,y)} H(w,z) \end{aligned}$$

with $$\gamma _z$$ inside $$\gamma _\rho $$.

The integrand in the second double integral has no singularities for $|w| > \rho$, since the poles are at $w=z$, $w=-1$, $w=-\alpha$, and they are all inside. For $|w| > \rho$ we have $\widetilde{{\mathcal {R}}}_N(w,z) = \mathcal {R}(w,z)$. From the asymptotic behavior in the RH problem 5.2 for *Y* we get

$$\begin{aligned} \begin{pmatrix} 1&0 \end{pmatrix} Y^{-1}(w) = \begin{pmatrix} 1&0 \end{pmatrix} \begin{pmatrix} w^{-N} &{} 0 \\ 0 &{} w^N \end{pmatrix} \left( I + {\mathcal {O}}(w^{-1})\right) = {\mathcal {O}}\left( w^{-N}\right) \end{aligned}$$

as $w \rightarrow \infty$, and thus by (5.8)

$$\begin{aligned} \widetilde{{\mathcal {R}}}_N(w,z) = {\mathcal {O}}\left( w^{-N}\right) \quad \text { as } w \rightarrow \infty . \end{aligned}$$

Also by the definition of *F*, see (6.3), we have $\left( F(w;x_2,y_2) \right) ^{-1} = {\mathcal {O}}(w^{y_2 - x_2})$ as $w \rightarrow \infty$. By combining with (7.5), we see that the full integrand in (7.19) is therefore $O\left( w^{-N+y_2-x_2 -1}\right)$ as $w \rightarrow \infty$. Since (*x*, *y*) is a point inside the hexagon, we have inequalities $-N< y_2 - x_2 < N$. Thus, since we are dealing with integers, the integrand is $O(w^{-2})$ as $w \rightarrow \infty$. Therefore the second double integral in (7.19) vanishes identically.

In the first double integral we deform $\gamma _\rho$ to $\gamma _w$ as in the statement of the proposition. We pick up a residue contribution at the pole $w=z$ for those $z \in \gamma _z$ that lie in the exterior of $\gamma _w$. This gives the first term in (7.18). The remaining double integral is the second term in (7.18). $\square$

### Proof of Proposition 7.7

We are now ready for the proof of Proposition 7.7 which, as already noted leads to the proof of Theorem 2.5. We also noted that it suffices to prove the proposition for $(\xi , \eta ) \in {\mathcal {L}}_{\alpha }$ with $\eta \le \frac{\xi }{2} \le 0$.

We first assume $\xi < 0$ and later deal with the modifications that are necessary for $\xi = 0$.

We write $x = x_N = (1 + \xi _N) N$, $y = y_N = (1+\eta _N) N$, and we are in the situation where

$$\begin{aligned} (\xi _N, \eta _N) \rightarrow (\xi ,\eta ) \in {\mathcal {L}}_{\alpha } \end{aligned}$$

with $\eta \le \frac{\xi }{2} < 0$. For *N* large enough, we then also have $(\xi _N, \eta _N) \in {\mathcal {L}}_{\alpha }$ with $\frac{\xi _N}{2} < 0$. We may also assume that $\eta _N \le \frac{\xi _N}{2} < 0$, because of symmetries as in Proposition 7.4 (b) and Proposition 7.6.

Then also $\Phi _N(z) := \Phi _{\alpha }(z;\xi _N,\eta _N)$ and the saddle $s_N := s(\xi _N,\eta _N;\alpha )$ vary with *N*, but in a controlled way. As $N \rightarrow \infty$ they tend to their limiting values $\Phi _{\alpha }(z;\xi ,\eta )$ and $s := s(\xi ,\eta ;\alpha )$.

In particular

7.20 $$\begin{aligned} \frac{1}{2\pi i} \int _{\overline{s}_N}^{s_N} H(z,z) dz \rightarrow \frac{1}{2\pi i} \int _{\overline{s}}^{s} H(z,z) dz \end{aligned}$$

as $$N \rightarrow \infty $$.

#### Low temperature regime with $\eta< \frac{\xi }{2} < 0$

Let $\gamma _{z}^{(N)}$ and $\gamma _{w,in}^{(N)}$, $\gamma _{w,out}^{(N)}$ be contours as in Corollary 6.6 (a) and Proposition 7.8 but corresponding to the parameters $(\xi _N,\eta _N)$ and $s = s_N$. Then by (7.15)

7.21 $$\begin{aligned} {\mathcal {I}}_N(x_N,y_N;H)= & {} \frac{1}{2\pi i} \int _{\overline{s}_N}^{s_N} H(z,z) dz + \frac{1}{(2\pi i)^2} \oint _{\gamma _z^{(N)}} dz\nonumber \\&\quad \oint _{\gamma _{w,in}^{(N)}} \frac{dw}{w-z} {\mathcal {R}}_N(w,z) \frac{F(z;x_N,y_N)}{F(w;x_N,y_N)} H(w,z) \nonumber \\&- \frac{1}{(2\pi i)^2} \oint _{\gamma _z^{(N)}} dz \oint _{\gamma _{w,out}^{(N)}} \frac{dw}{w-z} {\mathcal {R}}_N(w,z) \frac{F(z;x_N,y_N)}{F(w;x_N,y_N)} H(w,z)\nonumber \\ \end{aligned}$$

and in view of (7.20) it is enough to show that the two double integrals in (7.21) tend to 0 as $N \rightarrow \infty$.

By Corollary 5.6 (a) there exists a constant $C_1 > 0$ such that

7.22 $$\begin{aligned} \left| {\mathcal {R}}_N(w,z) \right| \le C_1 \left| e^{N(g(z) - g(w))} \right| . \end{aligned}$$

Also by definitions (6.4) and (6.3)

$$\begin{aligned} e^{N g(z)} F(z;x_N,y_N) e^{N \frac{\ell }{2}} = e^{N \Phi _N(z)} \times {\left\{ \begin{array}{ll} 1, &{} \text { if }x_N\text { is even}, \\ \left( \frac{z+\alpha }{z+1} \right) ^{1/2}, &{} \text { if }x_N\text { is odd}. \end{array}\right. } \end{aligned}$$

The contours stay away from $-\alpha$ and $-1$, therefore the extra factor in case $x_N$ is odd remains bounded and bounded away from 0. Combining this with (7.22) we obtain for some constant $C_2>0$,

7.23 $$\begin{aligned} \left| {\mathcal {R}}_N(w,z) \frac{F(z;x_N,y_N)}{F(w;x_N,y_N)} \right| \le C_2 \left| e^{N(\Phi _N(z)- \Phi _N(w))} \right| , \end{aligned}$$

for $$w \in \gamma _w^{(N)} := \gamma _{w,out}^{(N)} \cup \gamma _{w,in}^{(N)}$$, and $$z \in \gamma _z^{(N)}$$.

By Corollary 6.6 (a) the contours are in regions where $\mathop {\mathrm {Re}}\Phi _N(z)< \mathop {\mathrm {Re}}\Phi _N(s_N) < \mathop {\mathrm {Re}}\Phi _N(w)$, except for $\{ w, z \} \subset \{s_N, \overline{s}_N\}$, when there is equality. We can actually estimate (since the saddles are simple, and locally near the saddles we can follow steepest/ascent paths)

7.24 $$\begin{aligned} \begin{aligned} \mathop {\mathrm {Re}}\left( \Phi _N(w) - \Phi _N(s_N) \right) \ge C_3 |w-s_N|^2,&\quad \text { for } w \in \gamma _w^{(N)} \cap {\mathbb {C}}^+, \\ \mathop {\mathrm {Re}}\left( \Phi _N(z) - \Phi _N(s_N) \right) \le - C_3 |z-s_N|^2,&\quad \text { for } z \in \gamma _z^{(N)} \cap {\mathbb {C}}^+, \end{aligned} \end{aligned}$$

with a constant $C_3 > 0$ that is independent of *N*. By symmetry of the contours in the real axis, there are similar estimates for *w* and *z* in the lower half plane. Then it follows from (7.23) that the second double integral in (7.21) is exponentially small as $N \rightarrow \infty$ since $\gamma _{w,out}^{(N)}$ stays away from the saddle $s_N$.

The first double integral in (7.21) is not exponentially small, since the contours intersect at the saddles $s_N$ and $\overline{s}_N$. The dominant contribution comes from both *w* and *z* close to the saddle points. For a small enough $\delta > 0$, we may assume that $\gamma _{w,in}^{(N)} \cap D_{\delta }(s_N)$ and $\gamma _z^{(N)} \cap D_{\delta }(s_N)$ are straight line segments that meet at right angles. Then there are parametrizations with $-\delta< x < \delta$ and $-\delta< y < \delta$ such that $|z-s_N| = |x|$, $|w-s_N| = |y|$ and $|w-z| = \sqrt{x^2+y^2}$ for *z*, *w* on the contours in the $\delta$-neighborhood of $s_N$.

From estimates (7.23) and (7.24) we then easily get for some $C_4 > 0$,

$$\begin{aligned}&\left| \frac{1}{(2\pi i)^2} \oint _{\gamma _z^{(N)} \cap D_{\delta }(s_N)} dz \oint _{\gamma _{w,in}^{(N)} \cap D_{\delta }(s_N)} \frac{dw}{w-z} {\mathcal {R}}_N(w,z) \frac{F(z;x_N,y_N)}{F(w;x_N,y_N)} H(w,z) \right| \\&\quad \le C_4 \iint _{|x|^{2}+|y|^{2}\le \delta ^{2}} e^{-2C_3 N (x^2+y^2)} \frac{dxdy}{\sqrt{x^2 + y^2}} = 2\pi C_4 \int _0^{\delta } e^{-2C_3 N r^2} dr \end{aligned}$$

which tends to zero as $N \rightarrow \infty$. The same estimates hold for *w* and *z* near $\overline{s}_N$, or for *w* near $s_N$ and *z* near $\overline{s}_N$ or vice versa, and it follows that the first double integral in (7.21) tends to zero as $N \rightarrow \infty$.

Thus both double integrals tend to zero as $N \rightarrow \infty$. Because of (7.20) we then conclude that (7.14) holds.

#### High temperature regime with $\eta \le \frac{\xi }{2} < 0$

The proof in the high temperature regime is similar. We again use *N* dependent contours $\gamma _w^{(N)}$ and $\gamma _z^{(N)}$ satisfying the conditions of Corolarry 6.6 (b) and Proposition 7.9. Due to (7.18) and (7.20) we have to show that

7.25 $$\begin{aligned} \frac{1}{(2\pi i)^2} \oint _{\gamma _z^{(N)}} dz \oint _{\gamma _w^{(N)}} \frac{dw}{w-z} \widetilde{{\mathcal {R}}}_N(w,z) \frac{F(z;x_N,y_N)}{F(w;x_N,y_N)} H(w,z) \end{aligned}$$

tends to 0 as $N \rightarrow \infty$.

We recall that $w \mapsto \widetilde{{\mathcal {R}}}_N(w,z)$ is the analytic continuation of $w \mapsto {\mathcal {R}}_N(w,z)$ from the disk $|w| < \sqrt{\alpha }$ into the large disk $|w| < \rho$. It then follows from Corollary 5.6 (b) and (c) that

7.26 $$\begin{aligned} \widetilde{{\mathcal {R}}}_N(w,z) \le C_1 \left| e^{N(g(z) - g(w))} \right| \end{aligned}$$

whenever *w* is in the domain bounded by $\Sigma _0 \cup \Sigma _{-1}$ and $z \in {\mathbb {C}}$ with *w*, *z* bounded away from the branch points $z_{\pm }$. This is the estimate that is analogous to (7.22) in the low temperature regime.

By Corollary 6.6 (b) the contour $\gamma _w^{(N)}$ is inside $\Sigma _0 \cup \Sigma _{-1}$, and we can apply (7.26) in the estimation of (7.25). The rest of the proof is the same as in the low temperature regime with $\xi < 0$.

#### Case $\xi =0$ and $\eta < 0$

**Fig. 19:**
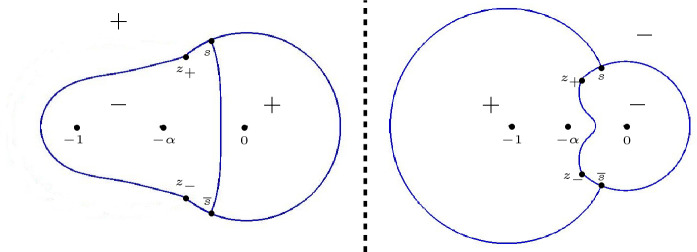
The sets $\mathcal {N}_{\Phi }$ (left) and $\mathcal {N}_{\Psi }$ (right) in the high temperature regime for $\xi = 0$ and $\eta < 0$. The signs of $\mathop {\mathrm {Re}}(\Phi _{\alpha } - \Phi _{\alpha }(s))$ (left) and $\mathop {\mathrm {Re}}(\Psi _{\alpha } - \Psi _{\alpha }(s))$ (right) are indicated with ±

For $\xi = 0$, the saddle is on the branch cut $\Sigma _0$ for the functions $\phi$ and $\Phi _{\alpha }$. We need additional deformation of contours to handle this case. For definiteness we focus on the high temperature regime, but the low temperature regime can be done similarly.

Note that $\Phi _{\alpha }(z) = \phi (z) - \eta \log z$ since $\xi = 0$, see (6.4). Since $\mathop {\mathrm {Re}}\phi (z) = 0$ for $z \in \Sigma _{0}$, and since $s \in \Sigma _{0}$, we have $\mathop {\mathrm {Re}}\Phi _{\alpha }(s) = - \eta \log \sqrt{\alpha }$, and furthermore the set $\mathcal {N}_{\Phi }$ (defined in (6.9)) is such that

$$\begin{aligned} \Sigma _{0} \subset \mathcal {N}_{\Phi }, \end{aligned}$$

see Fig. 19, left. To deal with this case we also need information about the set $\mathcal {N}_{\Psi } = \{z \in \mathbb {C} | \mathop {\mathrm {Re}}\Psi _{\alpha }(z) = \Psi _{\alpha }(s)\}$, see Fig. 19, right. For $\xi = 0$, we also have $\Sigma _{0} \subset \mathcal {N}_{\Psi }$.

**Fig. 20:**
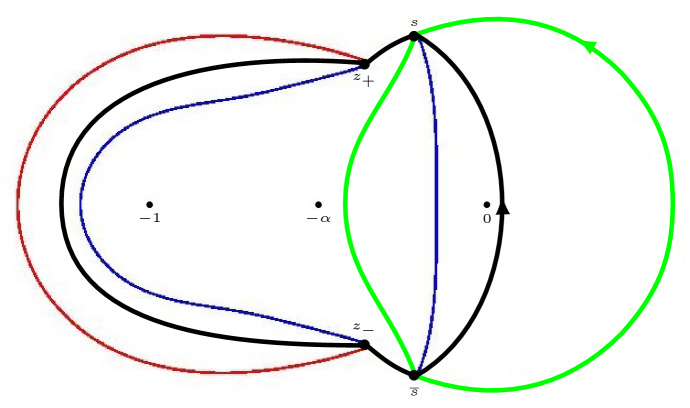
The contours $\gamma _{z}$ (green) and $\gamma _{w}$ (black) for $\xi = 0$ and $\eta < 0$ in the high temperature regime. They are drawn on top of $\mathcal {N}_{\Phi } \cup \Gamma _{-1}$

We treat the case $(0,\eta ) \in {\mathcal {L}}_{\alpha }$ with $\eta < 0$ as a limit of the case $(\xi ,\eta )$ with $\eta< \frac{\xi }{2} < 0$ that we considered before. In this limit the contours from Corollary 6.6 (b) can be chosen in such a way that they tend to contours $\gamma _z$ and $\gamma _w$ that partly overlap with $\Sigma _0$, such that the following hold (see Fig. 20 together with Fig. 19, left)

- $\gamma _w$ contains the subarcs
  $$\begin{aligned} \gamma _w \cap \Sigma _0 : \quad |w| = \sqrt{\alpha }, \, \arg s \le |\arg w| \le \arg z_+(\alpha ) \end{aligned}$$
  of $\Sigma _0$ and lies otherwise inside the (open) domain bounded by $\Sigma _0 \cup \Sigma _{-1}$, it goes around $-1$, and
  7.27 $$\begin{aligned} \begin{aligned} \mathop {\mathrm {Re}}\Phi _{\alpha }(w) > \mathop {\mathrm {Re}}\Phi _{\alpha }(s),&\quad w \in \gamma _w {\setminus } \Sigma _0, \\ \mathop {\mathrm {Re}}\Phi _{\alpha ,+}(w) = \mathop {\mathrm {Re}}\Phi _{\alpha }(s),&\quad w \in \gamma _w \cap \Sigma _0, \end{aligned} \end{aligned}$$
- $\gamma _z$ contains the subarc
  $$\begin{aligned} \gamma _z \cap \Sigma _0 : \quad |z| = \sqrt{\alpha }, \, -\arg s \le \arg z \le \arg s \end{aligned}$$
  of $\Sigma _0$ and lies otherwise inside the domain bounded by $\Sigma _0 \cup \Sigma _{-1}$, it goes around 0, and
  7.28 $$\begin{aligned} \begin{aligned} \mathop {\mathrm {Re}}\Phi _{\alpha }(z) < \mathop {\mathrm {Re}}\Phi _{\alpha }(s),&\quad z \in \gamma _z {\setminus } \Sigma _0, \\ \mathop {\mathrm {Re}}\Phi _{\alpha ,+}(z) = \mathop {\mathrm {Re}}\Phi _{\alpha }(s),&\quad z \in \gamma _z \cap \Sigma _0. \end{aligned} \end{aligned}$$

We want to estimate the double integral in (7.18) with $$x = x_N = (1+o(1)) N $$ and $$y = y_N = (1+\eta +o(1)) N$$ as $$N \rightarrow \infty $$. To avoid the use of *N* dependent contours as in the proofs above (which can be handled but would obscure the exposition) we assume $$x_N = N + O(1)$$ and $$y_N = (1 + \eta ) N + O(1)$$ as $$N \rightarrow \infty $$. Then by combining (6.3), (6.4) with (7.17) we find that $$\widetilde{R}_N(w,z) \frac{F(z;x_N,y_N)}{F(w;x_N,y_N)}$$ (which is the main part of the integrand in (7.18)) is equal to

7.29 $$\begin{aligned} e^{N (\Phi _{\alpha }(z) - \Phi _{\alpha }(w))} \times {\left\{ \begin{array}{ll} \begin{pmatrix} 1 &{} 0 \end{pmatrix} T^{-1}(w) T(z) \begin{pmatrix} 1 \\ 0 \end{pmatrix}, &{} \quad w \in \gamma _w, |w| < \sqrt{\alpha }, \\ \begin{pmatrix} 1 &{} -e^{2N \phi (w)} \end{pmatrix} T^{-1}(w) T(z) \begin{pmatrix} 1 \\ 0 \end{pmatrix},&\quad w \in \gamma _w, |w| > \sqrt{\alpha } \end{array}\right. } \end{aligned}$$

times a factor that remains bounded as $N \rightarrow \infty$. In (7.29) we take $+$ boundary values for $\Phi _{\alpha }$ and *T* whenever *w* and/or *z* are on $\Sigma _0$.

Because of (7.27) and (7.28) we see that (7.29) becomes exponentially small as $N \rightarrow \infty$ unless $w \in \gamma _w \cap \Sigma _0$ and $z \in \gamma _z \cap \Sigma _0$. Here we also use that $\mathop {\mathrm {Re}}\phi (w) < 0$ for $w \in \gamma _w$, $|w| > \sqrt{\alpha }$, and that *T* and $T^{-1}$ remain bounded as $N \rightarrow \infty$ if we stay away from the branch points, see Proposition 5.5 (b).

On $\gamma _z \cap \Sigma _0$ we use the identity

7.30 $$\begin{aligned} T_+(z) \begin{pmatrix} 1 \\ 0 \end{pmatrix} = e^{-2N \phi _+(z)} T_+(z) \begin{pmatrix} 0 \\ 1 \end{pmatrix} - T_-(z) \begin{pmatrix} 0 \\ 1 \end{pmatrix}, \quad z \in \Sigma _0, \end{aligned}$$

which follows from the jump (5.10) of *T* across $\Sigma _0$. Using (7.30) in (7.29) we split the integral over $\gamma _z \cap \Sigma _0$ into a sum of two integrals, and deform both integrals away from $\Sigma _0$.

The integral with the first term of the right-hand side of (7.30) is deformed to the interior, that is to a contour from $\overline{s}$ to *s* lying inside the disk $|z| = \sqrt{\alpha }$. The dominant part of the integrand is $e^{N(\Phi _{\alpha }(z) - 2 \phi (z))}$ and $\mathop {\mathrm {Re}}\Phi _{\alpha }(z) > \mathop {\mathrm {Re}}\Phi _{\alpha }(s)$ and $\mathop {\mathrm {Re}}\phi (z) > 0$ for *z* on the deformed contour. Fortunately, $\mathop {\mathrm {Re}}(\Phi _{\alpha }(z) - 2 \phi (z)) < \mathop {\mathrm {Re}}\Phi _{\alpha }(s)$, and this can be seen as follows. By (6.4) and (6.5) we have $\Phi _{\alpha } - 2 \phi = \Psi _{\alpha }$. Since $\xi = 0$ we also find from (6.4) and (6.5) that $\Phi _{\alpha } + \Psi _{\alpha } = - 2\eta \log z$. Thus indeed

$$\begin{aligned} \mathop {\mathrm {Re}}\Psi _{\alpha }(z) = - \mathop {\mathrm {Re}}\Phi _{\alpha }(z) - 2 \eta \log |z|< - \mathop {\mathrm {Re}}\Phi _{\alpha }(s) - 2 \eta \log |z| < \mathop {\mathrm {Re}}\Phi _{\alpha }(s) = -\eta \log \sqrt{\alpha } \end{aligned}$$

for *z* on the deformed contour, since $\mathop {\mathrm {Re}}\Phi _{\alpha }(z) > \mathop {\mathrm {Re}}\Phi _{\alpha }(s)$ || $|z|< \sqrt{\alpha } < 1$ there. We also use $\eta < 0$. Thus the deformed integral coming from the first term of (7.30) becomes small as $N \rightarrow \infty$.

The integral with the second term is moved outwards, again to a contour from $\overline{s}$ to *s* but now lying in $|z| > \sqrt{\alpha }$. Since $\Phi _{\alpha ,+} = \Psi _{\alpha ,-}$ the deformed integral has the exponentially varying factor $e^{N \Psi _{\alpha }}$. The contour can be taken such that $\mathop {\mathrm {Re}}\Psi _{\alpha }(z) < 0$ on the contour (see Fig. 19, right), and again the contribution becomes small as $N \rightarrow \infty$.

The integral (in the *w*-variable) over $\gamma _w \cap \Sigma _0$ can be dealt with analytic continuation only. We note that by (5.10)

$$\begin{aligned} \begin{pmatrix} 1&0 \end{pmatrix} T_{+}^{-1}(w) = \begin{pmatrix} e^{-2N \phi _-(w)}&-1 \end{pmatrix} T_{-1}^{-1}(w) \end{aligned}$$

which remains bounded if we analytically continue it to the exterior of $\Sigma _0$. We deform $\gamma _w \cap \Sigma _0$ to a contour from *s* to $z_+(\alpha )$ lying in the exterior of $\gamma _0$ together with its mirror image in the real, which is a contour from $z_-(\alpha )$ to $\overline{s}$. Since $\Phi _{\alpha ,+}(w) = \Psi _{\alpha ,-}(w)$ on $\Sigma _0$, the main term in the analytic continuation of (7.29) across $\gamma _w \cap \Sigma _0$ becomes $e^{-N \Psi _{\alpha }(w)}$. We are able to deform contours such that $\mathop {\mathrm {Re}}\Psi _{\alpha }(w) > 0$ on the deformed contour (from Fig. 19, right), where we also take note of the local behavior near the saddle points *s* and $\overline{s}$. The result is that the integral over the deformed contour becomes small as $N \rightarrow \infty$.

What remains are local contributions near the saddles *s* and $\overline{s}$ and also near the branch points $z_{\pm }(\alpha )$, since we cannot move $\gamma _w$ away from the branch points. The contributions from the saddles can be estimated as was done in detail for the low temperature regime with $\eta< \frac{\xi }{2} < 0$. The contributions from the branch points are estimated similarly, but we have to note that $T^{-1}(w) = {\mathcal {O}}(N^{1/6})$ for *w* close to the branch points, see Proposition 5.5 (b). This slight increase however still leads to a decay in the estimate and the conclusion is that all contributions vanish as $N \rightarrow \infty$.

#### Case $\xi = \eta =0$

For $\xi = \eta =0$ we are at the center of the hexagon. The center belongs to the liquid region only in the high temperature regime, and so this is what we assume. For $\xi = \eta =0$ the saddle coalesces with the branch point and the analysis requires additional deformation of contours. Note that by (6.4) we have

$$\begin{aligned} \Phi _{\alpha }(z) = \phi (z) \qquad \text { for } \xi = \eta = 0, \end{aligned}$$

and $$\mathop {\mathrm {Re}}\Phi _{\alpha }(s) = 0$$ where $$s = s(0,0;\alpha ) = z_+(\alpha )$$.

We approach this case as a limit of $(\xi , \eta ) \in {\mathcal {L}}_{\alpha }$ with $\eta \le \frac{\xi }{2} < 0$. In this limit the contours from Corollary 6.6 (b) tend to contours $\gamma _w$ and $\gamma _z$ that we may take as follows

- $\gamma _w$ contains $\Sigma _{-1}$ and its analytic continuation (which is a critical orthogonal trajectory, see Fig. 8) such that
  $$\begin{aligned} \mathop {\mathrm {Re}}\Phi _{\alpha }(w) > 0,&\quad w \in \gamma _w {\setminus } \Sigma _{-1}. \\ \mathop {\mathrm {Re}}\Phi _{\alpha }(w) = 0,&\quad w \in \Sigma _{-1}. \end{aligned}$$
- $$\gamma _z = \gamma _0$$ and
  $$\begin{aligned} \mathop {\mathrm {Re}}\Phi _{\alpha }(z) < 0,&\quad z \in \gamma _z {\setminus } \Sigma _{0}. \\ \mathop {\mathrm {Re}}\Phi _{\alpha }(z) = 0,&\quad z \in \Sigma _0. \end{aligned}$$

The integrand of the double integral in (7.18) behaves like (7.29) as $N \rightarrow \infty$. With the above choice of contours the integrand is exponentially small unless $w \in \Sigma _{-1}$ and $z \in \Sigma _0$. The case $z \in \Sigma _0$ is handled using the identity (7.30) that we also used in the case $\xi = 0$, $\eta < 0$. It allows us to split the integral into two integrals, deform one of them outwards and the other one inwards, and both deformed integrals have exponentially decaying integrands.

For $w \in \Sigma _{-1}$ we use the second line of (7.29) which tells us that the main *w*-dependent part is

$$\begin{aligned} e^{-N \Phi _{\alpha }(w)} \begin{pmatrix} 1&- e^{2N \phi (w)} \end{pmatrix} T^{-1}(w) \end{aligned}$$

which naturally splits into a sum (recall also $\Phi _{\alpha } = \phi$)

$$\begin{aligned} e^{-N \phi (w)} \begin{pmatrix} 1&0 \end{pmatrix} T^{-1}(w) - e^{N \phi (w)} \begin{pmatrix} 0&1 \end{pmatrix} T^{-1}(w) \end{aligned}$$

and a corresponding splitting and deformation of the *w*-integral. Namely the integral with the first term is deformed from $\Sigma _{-1}$ to a contour from $z_+(\alpha )$ to $z_-(\alpha )$ lying outside $\Sigma _{-1}$ (where $\mathop {\mathrm {Re}}\phi > 0$) and the integral with the second term is deformed inwards (where $\mathop {\mathrm {Re}}\phi < 0$).

Then there is exponentially decay on the deformed contours as $N \rightarrow \infty$, except for *w* and *z* near the branch points $z_{\pm }(\alpha )$. *T* and $T^{-1}$ have moderate growth there, both of ${\mathcal {O}}(N^{1/6})$. They combine to give an increase in $T^{-1}(w) T(z)$ of ${\mathcal {O}}(N^{1/3})$. Local estimates still lead to a decay in the integrals, as required.

This completes the proof of Proposition 7.7 in all cases.

### Proof of Theorem 2.8

#### Proof

With the coordinates in (2.25) (and the fact that $N \xi _N$ is assumed to be even) we can rewrite the kernel $K_{N}$ in (1.7) as

7.31 $$\begin{aligned} K_N(x_1,y_1,x_2,y_2)= -\frac{\chi _{u_1>v_2}}{2 \pi i} \oint _\gamma H_K(z,z;u_1,v_1,u_2,v_2) dz + {\mathcal {I}}_N(N \xi _N,N \eta _N; H_{K}) \end{aligned}$$

where ${\mathcal {I}}_N$ is as in (7.1) with

$$\begin{aligned} H_K(w,z;u_1,v_1,u_2,v_2)= \frac{(z+1)^{\lfloor \frac{u_1}{2}\rfloor }(z+\alpha )^{\lfloor \frac{u_1+1}{2}\rfloor }}{(w+1)^{\lfloor \frac{u_2}{2} \rfloor }(w+\alpha )^{\lfloor \frac{u_2+1}{2}\rfloor }} \frac{w^{v_2}}{z^{v_1+1}}. \end{aligned}$$

The first integral in (7.31) is independent of *N*. The asymptotic behavior of ${\mathcal {I}}_N(N \xi _N,N \eta _N; H_{K})$ as $N\rightarrow \infty$ is already computed in Proposition 7.7. The first integral and the limit from Proposition 7.7 can be combined naturally into one single integral, which is the right-hand side of (2.26). This finishes the proof. $\square$

## Proof of Proposition 1.1

### Proof of Proposition 1.1

This is a special case of Theorem 4.7 in [34]. To identify the formula in [34] with (1.7), we first of all note that $p=1$ and $K_N$ is a scalar kernel. We have to identify $(m,x,m',y)$ and (*N*, *M*, *L*) in [34] with $(x_1,y_1,x_2,y_2)$ and (*N*, *N*, 2*N*) in the setting of our paper.

Furthermore, for $0 \le i < j \le 2N$, the notation $A_{i,j}(z)$ in [34] stands for $A_{i,j}(z) = \prod \nolimits _{m=i}^{j-1} a_m(z)$ where $a_m(z) = z+\alpha$ if *m* is even, and $a_m(z) = z+1$ if *m* is odd. This gives

$$\begin{aligned} A_{x_2, x_1}(z) = (z+1)^{\lfloor \frac{x_1}{2} \rfloor -\lfloor \frac{x_2}{2} \rfloor } (z+\alpha )^{\lfloor \frac{x_1+1}{2} \rfloor -\lfloor \frac{x_2+1}{2}\rfloor } \end{aligned}$$

which appears in the single integral in (1.7), and similarly

$$\begin{aligned} A_{x_2,2N}(w)&= (w+1)^{N -\lfloor \frac{x_2}{2} \rfloor } (w+\alpha )^{N - \lfloor \frac{x_2+1}{2}\rfloor } \\ A_{0,x_1}(z)&= (z+1)^{\lfloor \frac{x_1}{2} \rfloor } (z+\alpha )^{\lfloor \frac{x_1+1}{2} \rfloor } \end{aligned}$$

which is part of the double integral in (1.7).

Finally, according to [34, Theorem 4.7], $R_N$ is the reproducing kernel for polynomials of degree $\le N-1$ with weight $\displaystyle \frac{A_{0,L}(z)}{z^{M+N}} = \frac{(z+1)^N(z+\alpha )^N}{z^{2N}}$ on $\gamma$, as $M=N$ and $L=2N$. It means that $R_N(w,z)$ is a bivariate polynomial of degree $\le N-1$ in both variables that is uniquely characterized by the property that

A.1 $$\begin{aligned} \frac{1}{2 \pi i} \oint _{\gamma } R_N(w,z) \frac{(z+1)^N(z+\alpha )^N}{z^{2N}} q(z) dz = q(w) \end{aligned}$$

for every polynomial *q* of degree $\le N-1$, see Lemma 4.6 in [34]. Since all orthogonal polynomials $p_n$ of degrees $n \le 2N$ exist (we prove this in Proposition 5.1), the sum in (1.8) is well-defined, and by orthogonality using (1.9) it defines a kernel with the required reproducing property (A.1).

The expression in the second line of (1.8) is known as the Christoffel–Darboux formula, and it continues to hold for non-Hermitian orthogonality on a contour, with the same proof as for usual orthogonal polynomials on the real line. $\square$
